# Development of a Risk Predictive Model for Evaluating Immune Infiltration Status in Invasive Thyroid Carcinoma

**DOI:** 10.1155/2022/5803077

**Published:** 2022-06-03

**Authors:** Runming Pang, Chunxin Qin

**Affiliations:** Department of Thyroid Breast Surgery, Weihai Municipal Hospital, Cheeloo College of Medicine, Shandong University, Weihai 264299, China

## Abstract

**Aims:**

This study aimed to reveal the molecular characteristics and potential biomarker of immune-activated and immunosuppressive invasive thyroid carcinoma.

**Methods:**

Expression and clinical data for invasive thyroid carcinoma were obtained from the TCGA database. Tumor samples were divided into immune-activated or immunosuppressive groups based on the immune enrichment score calculated by ssGSEA. Differentially expressed genes (DEGs) between tumor vs. normal groups or between immune-activated vs. immunosuppressive groups were screened, followed by functional enrichment. Immune infiltration was evaluated using the ESTIMATE, CIBERSORTx, and EPIC algorithms, respectively. A random forest algorithm and Lasso cox analysis were used to identify gene signatures for risk model construction.

**Results:**

Totally 1171 DEGs were screened between tumor vs. normal groups, and multiple tumorigenesis-associated pathways were significantly activated in invasive thyroid carcinoma. Compared to immune-activated samples, immunosuppressive samples showed higher tumor purity, lower immune/stromal scores, and lower expression of immune markers, as well as lower infiltration abundance of CD4+ T cells and CD8+ T cells. A risk model based on a 12-immune signature (CCR7, CD1B, CD86, CSF2RB, HCK, HLA-DQA1, LTA, LTB, LYZ, NOD2, TNFRSF9, and TNFSF11) was developed to evaluate the immune infiltration status (AUC = 0.998; AUC of 0.958 and 0.979 in the two external validation datasets), which showed a higher clinical benefit and high accuracy. Immune-activated samples presented lower IC50 value for bortezomib, MG.132, staurosporine, and AZD8055, indicating sensitivity to these drugs.

**Conclusion:**

A 12-gene-based immune signature was developed to predict the immune infiltration status for invasive thyroid carcinoma patients and then to identify the subsets of invasive thyroid carcinoma patients who might benefit from immunotherapy.

## 1. Introduction

Thyroid carcinoma is the most common malignant tumor of the endocrine system, originating in the thyroid follicular epithelium or parafollicular epithelium [1]. Global Cancer Statistics indicate that there are an estimated 586,202 new cases of thyroid carcinoma in 2020, accounting for 3.0% of the global cancer burden [2]. In China, thyroid carcinoma ranks the fourth most common type of cancer in females and the ninth in males, accounting for 38% of global new cases of thyroid carcinoma [3]. Thyroid carcinoma can be divided into four histological types based on their tumor origin and differentiation, including papillary, follicular, medullary, and anaplastic thyroid carcinoma [4]. Of which, papillary thyroid carcinoma (PTC) is the most frequent type, accounting for 85% to 90% of thyroid carcinoma. Due to relatively inert clinical biological behavior, the prognosis of PTC is generally excellent, with a 10-year-survival rate of over 90% [5]. However, about 5%–34% patients with differentiated thyroid carcinoma present extrathyroidal extension, which negatively affects the prognosis [6–8]. Extrathyroidal lesions may implicate crucial structures in the central neck, and gross residual lesions postoperatively may lead to recurrence [9]. Hence, it is necessary to propose effective approaches to improve the prognosis of invasive thyroid carcinoma.

Reportedly, a risk predictive model contributes to make corresponding decisions for treatment and management of patients with thyroid carcinoma by dividing patients into different risk groups [10, 11]. Several risk models have been established for thyroid carcinoma [12–14]. For example, Shen et al. established a six-genotype genetic molecular (involves mutations in RAS, BRAF V600E, and TERT promoter) prognostic system for PTC, which helps to identify the subsets of PTC patients with the highest risk of invasion for personalized and precise treatment [15]. Wang et al. constructed a risk predictive model based on the N^6^-methyladenosine-related signature to predict the disease-free survival for PTC patients [14]. However, few studies focus on immune-related genes signature in the risk prediction ofinvasive thyroid carcinoma.

Tumor cells escaping immune surveillance and destruction present a hallmark of tumors. Immune cells in the tumor microenvironment affect the development and progression of tumors by secreting soluble cytokines to mediate the proliferation of tumor cells [16]. Immunotherapy has shown survival benefits by unleashing the immune system and activating cytotoxic lymphocytes to kill cancer cells [17]. However, only part of patients benefit from immunotherapy. Therefore, this study proposed a risk model based on immune-related signature to evaluate the immune activation and immunosuppression status for invasive thyroid carcinoma patients. This will help to identify the subsets of invasive thyroid carcinoma patients who might benefit from immunotherapy or not, so as to get personalized and precise treatment.

## 2. Material and Methods

### 2.1. Data Acquisition and Preprocessing

Data of genes expression, clinical phenotype, mutation, and survival for thyroid carcinoma in The Cancer Genome Atlas (TCGA) database were downloaded from UCSC Xena (https://xenabrowser.net/datapages/). From which, invasive thyroid carcinoma samples (extrathyroid carcinoma present extension status ≥ T3) were selected for analysis. Totally 176 samples with both expression data and clinical phenotype were included, containing 154 tumor samples and 22 normal samples. Two microarray datasets, GSE35570, and GSE65074 were downloaded from the Gene Expression Omnibus (GEO) database. These two microarray datasets were used to validate the risk-score model.

### 2.2. Differential Expression Analysis

Principal component analysis (PCA) dimension reduction was performed for the expression data. Then, differential expression analysis was performed using the Limma package in R to screen the differentially expressed genes (DEGs) between tumor vs. normal groups or between immune activation vs. immunosuppression groups, respectively. The DEGs were screened with the cut-off value of |logFC| > 2 and adjusted *P* < 0.01.

### 2.3. Functional Enrichment Analysis

Enrichment analysis for gene ontology (GO) annotation terms and KEGG pathways was conducted using the clusterProfiler package [18] in R to uncover the involved functions of DEGs. The significantly enriched GO terms and pathways were selected with *P* < 0.05. Gene set enrichment analysis (GSEA) was also performed using the clusterProfiler package in R to reveal the differences in terms of pathways between groups.

### 2.4. Immune-Activated and Immunosuppressive Tumors

Based on the 29 immune-related gene sets (Table S1), single-sample GSEA (ssGSEA) was conducted using the GSVA package [19] to calculate the enrichment score for each tumor sample. Then, the 154 tumor samples were assigned into immune-activated and immunosuppressive groups using sparse hierarchical clustering. After PCA dimension reduction, the DEGs between immune activation vs. immunosuppression groups were screened with the cut-off value of |logFC| > 1.5 and adjusted *P* < 0.01.

### 2.5. Evaluation of Immune Infiltration

The stromal-score, immune-score, and tumor-purity of each tumor sample were evaluated using the ESTIMATE package in R. Composition and infiltration abundance of immune cells of each tumor sample were evaluated using CIBERSORTx and EPIC methods provided in TIMER 2.0 database [20].

### 2.6. Feature Genes Screening Using Machine Learning

Immune-related genes (IRGs, Table S2) were obtained from the ImmPort (https://immport.niaid.nih.gov) database [21]. Then, IRGs were merged with DEGs that screened between immune activation vs. immunosuppression groups, and the overlapped genes were considered as DEIRGs. DEIRGs were then analyzed by a random forest algorithm provided in the Boruta package [22] to screen feature genes. The feature genes were then uploaded to the STRING database to reveal the interactions among their encoded proteins.

### 2.7. Construction of Risk-Score Model

The optimal gene signature was further identified from the feature genes using the Lasso cox analysis provided in the glmSparseNet package. The gene signature identified by Lasso cox analysis was used to construct a risk-score model. A receiver operator characteristic (ROC) curve was plotted using the pROC package [23] to evaluate the predictive performance of the risk-score model. Decision curve analysis (DCA) and the clinical impact curve were plotted using the DecisionCurve package (version 1.3) to evaluate the clinical benefit. The GSE35570 and GSE65074 datasets were used as external datasets for validation. The invasive tumor samples in these two datasets were divided into immune-activated and immunosuppressive groups based on the methods mentioned above. Based on the identified gene signature, a risk-score model was established based on these two datasets, followed by evaluating the predictive performance using the ROC curve, DCA, and clinical impact curve.

### 2.8. Mutation Analysis and Drug Sensitivity Analysis

In order to investigate the possible causes of differential expression for signature genes, mutation analysis was conducted using the maftools package [24] based on the somatic SNP data in the TCGA database. Based on the Genomics of Drug Sensitivity in Cancer (GDSC) database, the drug sensitivity of each sample was evaluated by calculating the IC50 value using the OncoPredict package (version 0.2) to investigate the degree of patient response to drugs.

### 2.9. Statistical Analysis

The differences in stromal-score, immune-score, tumor-purity, immune markers (including T cell stimulators, T cell inhibitors, and histocompatibility complexes) expression, gene mutation, and drug sensitivity between immune-activated and immunosuppressive groups were compared using the *T*-test. *P* < 0.05 shows the statistical significance.

## 3. Results

### 3.1. Genes Differentially Expressed in Invasive Thyroid Carcinoma Compared to Normal Control

**Figure 1:**
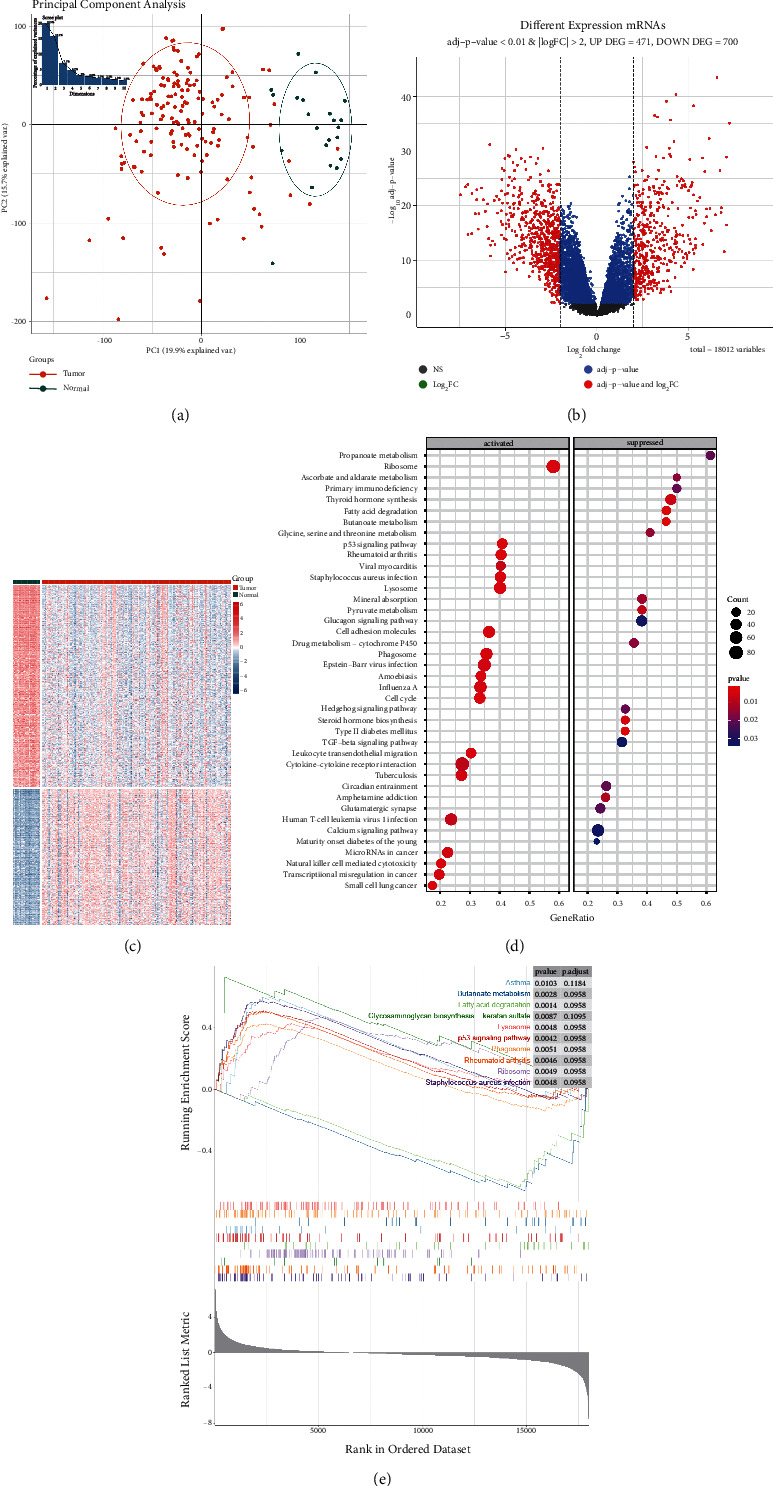
Differential expression analysis between invasive thyroid carcinoma and normal control. (a) PCA score plot shows the samples difference between two groups; (b) volcano plot shows the numbers of DEGs; (c) heatmap shows the expression pattern of DEGs between the two groups; (d) bubble diagram shows the top 20 activated and suppressed pathways in GSEA analysis; (e) the top 10 pathways in GSEA analysis ranked by NES value.

In order to investigate the differences between invasive thyroid carcinoma and normal control at the molecular level, we screened the DEGs between these two groups first. PCA indicated that samples from the same group were clustered together, indicating that gene expression in these two groups was significantly distinguished (Figure 1(a)). Totally 1171 DEGs were screened, of which 471 genes were upregulated while 700 genes were downregulated in invasive thyroid carcinoma compared to normal control (Figure 1(b)). The bidirectional clustering heatmap confirmed that these DEGs could clearly distinguish the samples into two groups (Figure 1(c)). Enrichment analysis was performed to investigate the involved functions of these DEGs. The results showed that DEGs were enriched in GO annotation terms, including cell junction assembly, synapse assembly, collagen-containing extracellular matrix, extracellular matrix structural constituent, and glycosaminoglycan binding (Figures S1A-C), and in KEGG pathways, such as neuroactive ligand-receptor interaction, ECM-receptor interaction, and cell adhesion molecules (Figure S1D). The DEGs might contribute to tumor invasion by these biological functions and pathways.

GSEA analysis revealed significant differences in terms of pathways between tumor and normal groups. As shown in Figures 1(d) and 1(e), various tumorigenesis-associated pathways were significantly activated in invasive thyroid carcinoma samples, including p53 signaling pathway, cell adhesion molecules, cytokine-cytokine receptor interaction, microRNAs in cancer, and transcriptional misregulation in cancer. Multiple metabolism pathways, such as propanoate metabolism, butanoate metabolism, and thyroid hormone synthesis, were markedly inhibited in invasive thyroid carcinoma samples.

### 3.2. Immune-Activated and Immunosuppressive Invasive Thyroid Carcinoma

**Figure 2:**
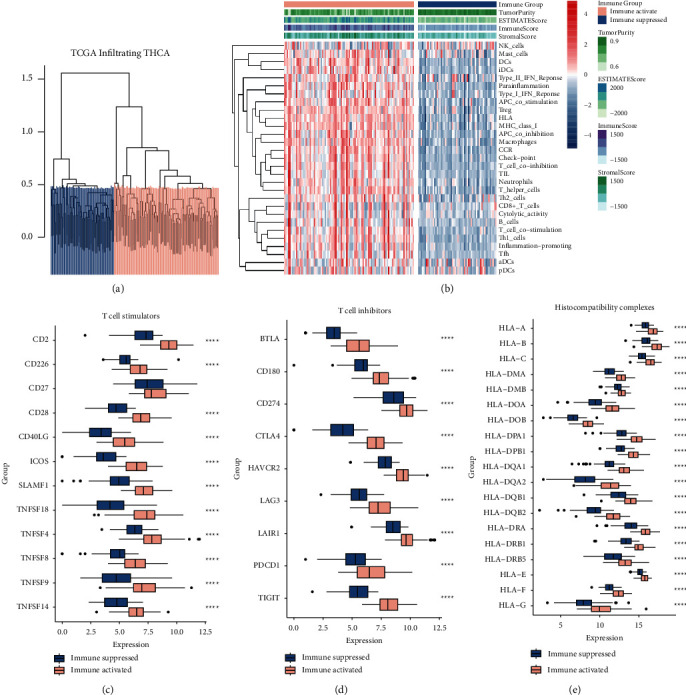
Immune-activated and immunosuppressive invasive thyroid carcinoma. (a) Sparse hierarchical clustering for all tumor samples based on immune enrichment score calculated by ssGSEA; (b) heatmap shows the expression pattern of 29 immune-related genes sets between immune-activated and immunosuppressive groups; expression of T cell stimulators (c), T cell inhibitors (d), and histocompatibility complexes (e) between immune-activated and immunosuppressive groups.

The immune enrichment score of each tumor sample was calculated using ssGSEA, and then the tumor samples were assigned into immune-activated and immunosuppressive groups using sparse hierarchical clustering (Figure 2(a)). Compared to immune-activated samples, immunosuppressive samples showed lower immune, stromal scores, and expression levels of immune-related gene sets, while showing higher tumor purity (Figure 2(b)). Additionally, expression levels of T cell stimulators, T cell inhibitors, and histocompatibility complexes were all decreased compared to immune-activated samples (Figures 2(c)–2(e)). These results suggested that immune-activated patients might be more likely to benefit from immunotherapy.

### 3.3. Genes Differentially Expressed between Immune-Activated and Immunosuppressive Groups

**Figure 3:**
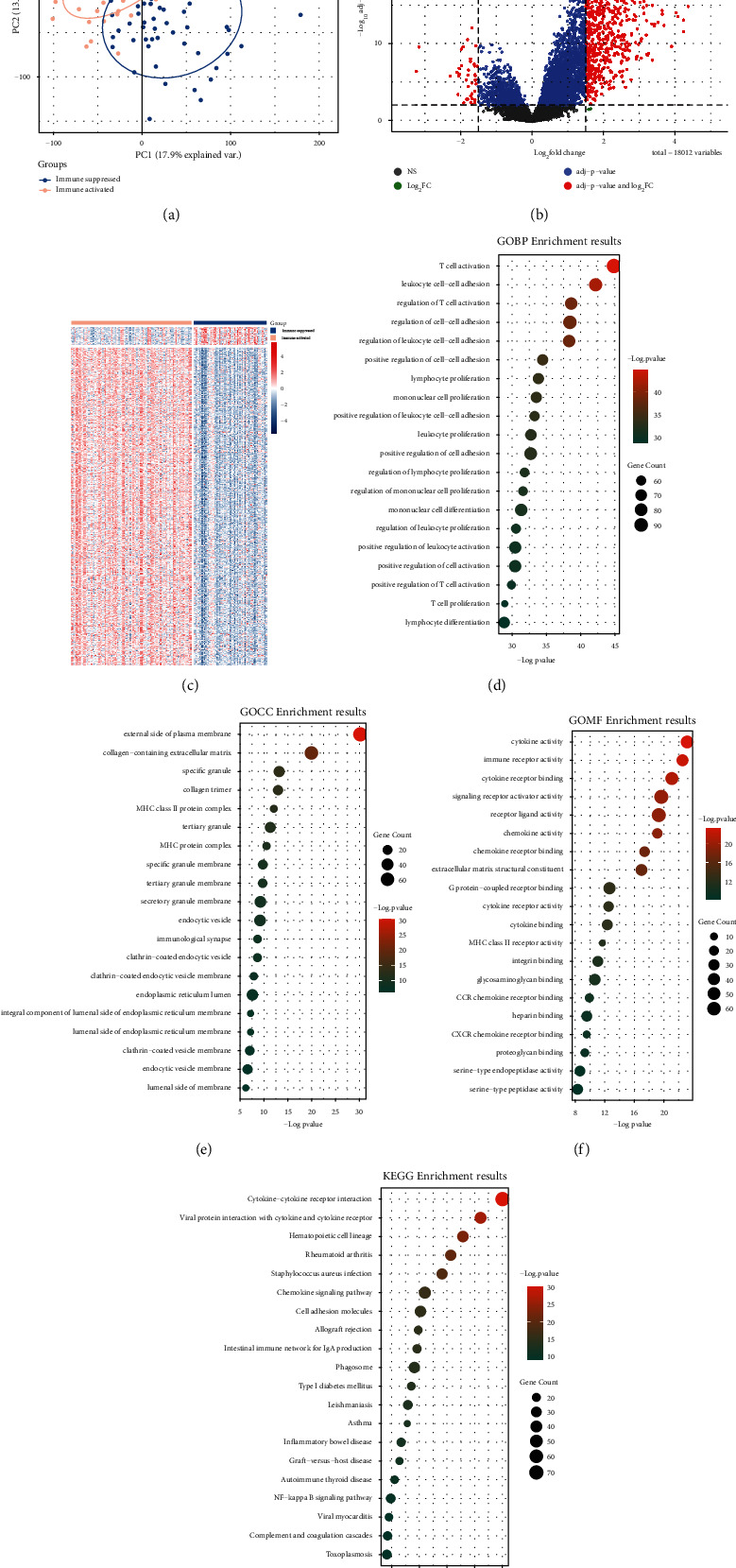
Differential expression analysis between immune-activated and immunosuppressive groups. (a) PCA score plot shows the samples difference between two groups; (b) volcano plot shows the numbers of DEGs; (c) heatmap shows the expression pattern of DEGs between the two groups; the top 20 enriched biological processes (d), cellular component (e), and molecular function (f) terms in gene ontology annotation; (g) the top 20 enriched KEGG pathways.

Immune-activated and immunosuppressive samples were distinguished in PCA, indicating the practicability and successfulness of such groups (Figure 3(a)). In total, 765 DEGs were screened between immune-activated and immunosuppressive groups, and the majority of genes (724 genes) were upregulated in immune-activated groups (Figures 3(b) and 3(c)). Functional enrichment indicated that these DEGs were involved in multiple immune related biological function, including T cell activation, leukocyte cell-cell adhesion, lymphocyte proliferation, MHC protein complex, cytokine activity, and immune receptor activity. (Figures 3(d)–3(f)). These DEGs also significantly enriched in cytokine-cytokine receptor interaction, chemokine signaling pathway, and cell adhesion molecules pathways (Figure 3(g)). GSEA analysis indicated that cell adhesion molecules, cytokine-cytokine receptor interaction, Th17 cell differentiation, and chemokine signaling pathways were markedly activated in immune-activated groups (Figure S2). These findings suggested that immune-related genes and pathways were markedly upregulated in immune-activated groups.

### 3.4. Immune Infiltration between Immune-Activated and Immunosuppressive Groups

**Figure 4:**
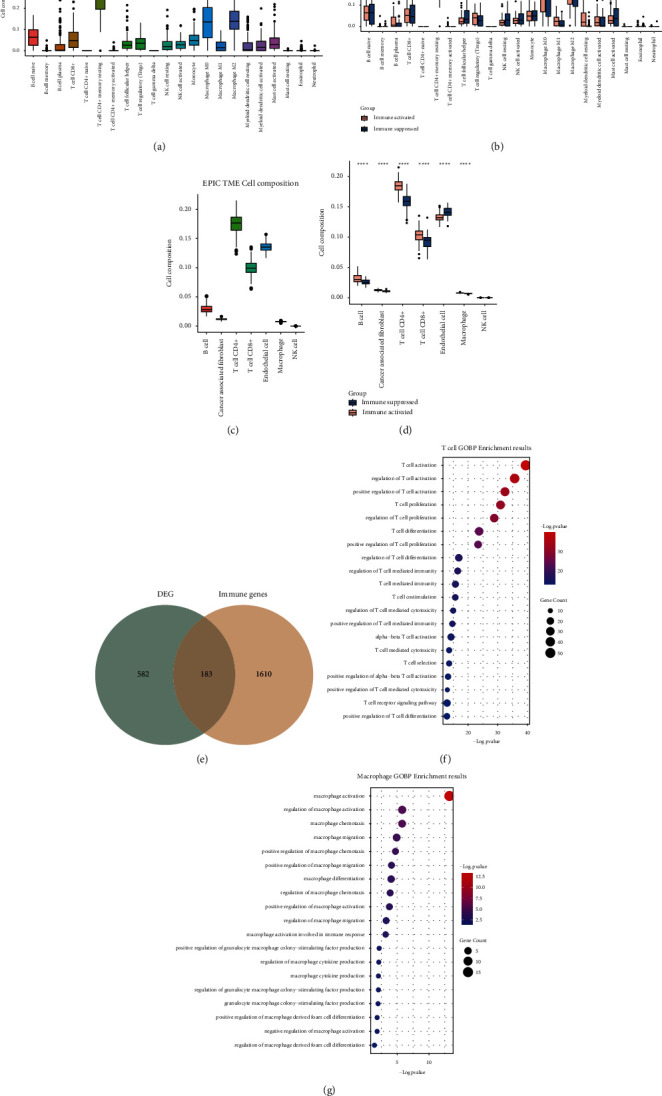
Evaluation of tumor immune microenvironment. (a) Landscape of 22 kinds of tumor-infiltrating immune cells evaluated by CIBERSORTx; (b) infiltration abundance of 22 immune cells differed between immune-activated and immunosuppressive groups; (c) landscape of 6 kinds of tumor-infiltrating immune cells evaluated by EPIC; (d) differences on infiltration abundance of 6 immune cells between immune-activated and immunosuppressive groups; (e) Venn analysis for screening the DEIRGs between immune gene sets and DEGs; (f) the enriched T cells (f) and macrophages (g) related biological processes for DEIRGs.

Immune cells composition for all tumor samples was evaluated using CIBERSORTx. Resting memory CD4+ T cells, CD8+ T cells, naive B cells, and M0/M2 macrophages accounted for high composition (Figure 4(a)). Infiltration abundance of follicular helper T cells, resting NK cells, resting myeloid dendritic cells, and M1 macrophages in immune-activated groups significantly differed from immunosuppressive groups (Figure 4(b)). Based on the EPIC tool, we found CD4+ T cells, CD8+ T cells, and endothelial cells showed higher composition in tumor samples (Figure 4(c)). Of which, immune-activated samples showed higher infiltration abundance of CD4+ T cells and CD8+ T cells compared to that of immunosuppressive samples (Figure 4(d)), indicating high antitumor immunity in immune-activated samples.

### 3.5. Differentially Expressed Immune-Related Genes (DEIRGs)

From the ImmPort database, 1793 IRGs were obtained. Of which, 183 DEIRGs were screened by filtrating with the 765 DEGs between immune-activated and immunosuppressive groups (Figure 4(e)). These DEIRGs were significantly enriched in various biological processes associated with T cells (Figure 4(f)), including T cell activation/proliferation/differentiation, T cell mediated immunity, and T cell mediated cytotoxicity. Various macrophage-associated biological processes were also enriched (Figure 4(g)), including macrophage activation/chemotaxis/migration/differentiation, macrophage activation involved in immune response, and macrophage cytokine production. This was consistent with the high infiltration composition of T cells and macrophages in the tumor microenvironment.

### 3.6. Machine Learning and Model Construction

**Figure 5:**
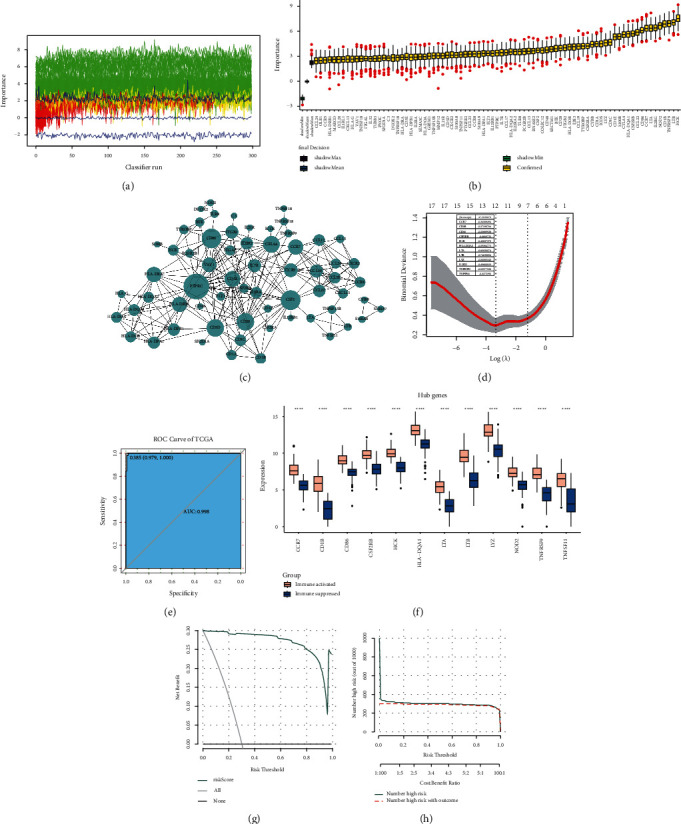
Machine learning and model construction. (a, b) Screening of feature genes by importance calculated by Boruta algorithm; (c) the protein-protein interaction network for feature genes; (d) the optimal gene signature identified by Lasso cox analysis to construct risk-score model; (e) ROC curve shows the predictive performance of risk-score model; (f) genes in model differentially expressed between immune-activated and immunosuppressive groups; decision curve analysis (g) and clinical impact curve (h) show the clinical benefits and accuracy of the model.

Using a random forest algorithm provided in Boruta, we screened 81 feature genes from the 183 DEIRGs (Figures 5(a) and 5(b)). The protein-protein interactions were further predicted. Of which, PTPRC, CSF2, CD86, CCR7, and CTLA4 were the top five hub genes with higher degree (Figure 5(c)). Lasso cox analysis identified 12 optimal genes signature (Figure 5(d)), and a risk-score model was constructed with the following formula: risk score = 47.21266778 -1.34308494 ∗ CCR7 -0.57530764 ∗ CD1B -0.03889928 ∗ CD86 -0.06197250 ∗ CSF2RB -1.43837279 ∗ HCK -0.09384775 ∗ HLA-DQA1 -0.83051074 ∗ LTA-0.74620894 ∗ LTB -0.61083543 ∗ LYZ -0.49800153 ∗ NOD2 -0.13977936 ∗ TNFRSF9 -0.03725910 ∗ TNFSF11. The ROC curve indicated that the risk-score showed a good performance to evaluate the risk of immune-activated and immunosuppressive status, with an area under curve (AUC) of 0.998 (Figure 5(e)). All the 12 genes in model were highly expressed in immune-activated samples than immunosuppressive samples (Figure 5(f)). DCA curve indicated that the risk score model showed higher clinical benefit (Figure 5(g)). The clinical impact curve confirmed that the prediction result of risk-score model was close to clinical reality, suggesting a high accuracy (Figure 5(h)). These findings suggested that the risk-score model had a good performance to evaluate the immune-activated or immunosuppressive status for invasive thyroid carcinoma.

### 3.7. Validation of Risk-Score Model by External Datasets

**Figure 6:**
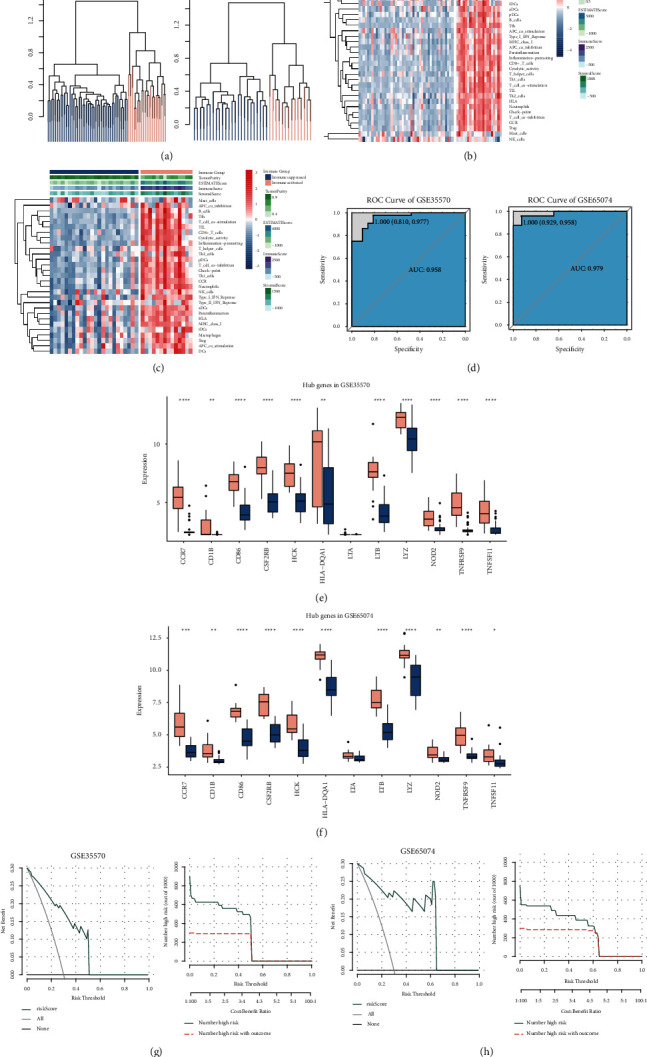
External validation for the model. (a) Sparse hierarchical clustering for all tumor samples in GSE35570 (left) and GSE65074 (right); heatmap shows the expression pattern of 29 immune-related genes sets between immune-activated and immunosuppressive groups in GSE35570 (b) and GSE65074 (c) datasets; (d) ROC curve shows the predictive performance of risk-score model in GSE35570 (left) and GSE65074 (right) dataset; genes in model differentially expressed between immune-activated and immunosuppressive groups in GSE35570 (e) and GSE65074 (f) datasets; decision curve analysis and clinical impact curve show the clinical benefits and accuracy of the model based on GSE35570 (g) and GSE65074 (h) datasets.

To avoid overfitting of the model in the TCGA-cohort, two external GEO datasets (GSE35570 and GSE65074) were used to validate the model. First, the tumor samples in these two datasets were clustered into immune-activated or immunosuppressive groups, respectively (Figure 6(a)). Similarly, immune-activated samples showed a high expression of immune-related gene sets and a high immune score, but it showed a low tumor purity (Figures 6(b) and 6(c)). Risk-score models were constructed as the formula mentioned above. As shown in Figure 6(d), the model had a good performance to evaluate the risk of immune-activated and immunosuppressive status, with an AUC of 0.958 in GSE35570 dataset and AUC of 0.979 in GSE65074 dataset. Consistently, all the 12 genes in model were highly expressed in immune-activated groups (Figures 6(e) and 6(f)). Furthermore, DCA curves (Figure 6(g)) and clinical impact curves (Figure 6(h)) showed that the model showed higher clinical benefit and a high accuracy to evaluate the risk of immune-activated and immunosuppressive status.

### 3.8. Drug Sensitivity Analysis

**Figure 7:**
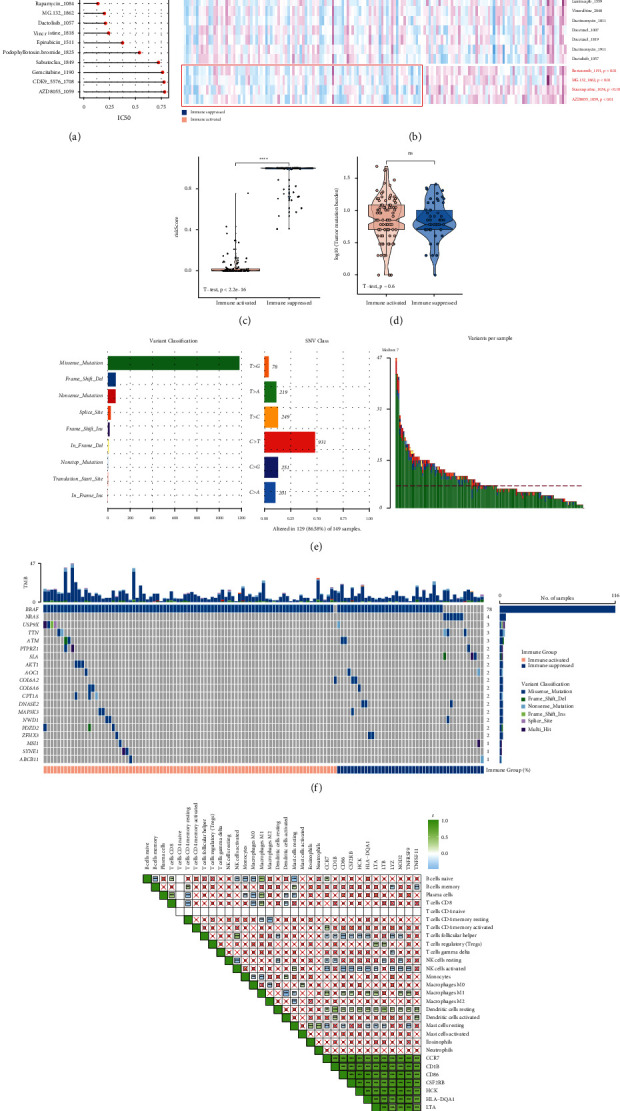
Mutation analysis and drug sensitivity analysis. (a) The drugs with IC50 < 1 for all tumor samples in TCGA cohort; (b) heatmap shows the differences on IC50 value (evaluation for drug sensitivity) for immune-activated and immunosuppressive patients; (c) risk score significantly differed between immune-activated and immunosuppressive patients; (d) tumor mutation burden shows no differences between immune-activated and immunosuppressive patients; (e) statistical result of somatic mutation for all tumor samples in TCGA cohort; (f) waterfall plot shows the top 20 mutation genes; (g) correlation analysis between tumor-infiltrating immune cells evaluated by CIBERSORTx and genes in model.

In order to screen possible drugs for the treatment of invasive thyroid carcinoma, the drugs with IC50 < 1 for all tumor samples were analyzed (Figure 7(a)). From which, four drugs, including bortezomib, MG.132, staurosporine, and AZD8055, showed significant differences between immune-activated and immunosuppressive groups (Figure 7(b)). All these four drugs showed lower IC50 values in immune-activated samples, suggesting that immune-activated patients were more sensitive to these drugs. Immunosuppressive group showed significant high-risk score than immune-activated group (Figure 7(c)). This also confirmed that immune-activated patients might be more likely to benefit from drug therapy.

### 3.9. Mutation Analysis and Correlation Analysis

Since all the genes in the model were highly expressed in the immune-activated group compared to immunosuppressive group, we analyzed the possible reasons for the gene expression differences from the perspective of gene mutation. There were no differences on tumor mutation burden between immune-activated and immunosuppressive groups (Figure 7(d)). Missense mutation accounted for the majority of the variants (Figure 7(e)). Among the top 20 mutation genes, mutation in BRAF gene accounted for 78% of tumor samples (Figure 7(f)), suggesting that BRAF mutation might be correlated with the development of invasive thyroid carcinoma. Genes in the model were not found in the top 20 mutation genes, suggesting that the expression differences of these genes might not be caused by gene mutation. We further analyzed the correlations between genes in the model and tumor-infiltrating immune cells (Figure 7(g)), and we found that most of the genes were significantly correlated with the follicular helper T cells, activated NK cells, M1 macrophages, resting dendritic cells, and resting mast cells.

## 4. Discussion

Extrathyroidal extension of thyroid carcinoma has been demonstrated to show an adverse impact on the prognosis of patients [25]. In this study, we first investigated the genes and pathways that dysregulated in invasive thyroid carcinoma based on the data in the TCGA database (thyroid carcinoma samples with extrathyroid carcinoma present extension status ≥ T3). A large-scale of gene expression pattern changed in invasive thyroid carcinoma compared to control. Additionally, various tumorigenesis-associated pathways were significantly activated, including the p53 signaling pathway, cell adhesion molecules, cytokine-cytokine receptor interaction, and transcriptional misregulation in cancer. Therefore, we suggested that these dysregulated genes might mediate the development and progression of invasive thyroid carcinoma by affecting tumorigenesis-associated pathways. Notably, the thyroid hormone synthesis pathway was found to be inhibited in invasive thyroid carcinoma samples. Thyroid stimulating hormone (TSH) is the most important hormone regulating thyroid gland function. It activates the cAMP pathway and regulates hormone synthesis and proliferation of thyroid follicular cells by binding TSH receptor on the membrane of thyroid follicular cells, thus affecting the onset or progression of follicular cells-originated thyroid cancer [26, 27].

Based on the immune enrichment score, all the invasive tumor samples were divided into immune-activated and immunosuppressive groups. Immune-activated samples showed lower tumor purity, but it showed higher immune/stromal scores and higher expression of T cell stimulators, T cell inhibitors, and histocompatibility complexes. Moreover, immune-activated samples showed higher infiltration abundance of CD4+ T cells and CD8+ T cells. Increased studies have indicated that patients with high immune infiltration (hot tumors) were more likely to benefit from immunotherapy, while immunosuppressive (cold tumor) tumors were prone to be resistant to immune checkpoint blocker [28, 29]. Immune infiltration status and levels in TME showed significant correlations with the prognosis of tumor patients [30]. Tumor infiltrating lymphocytes (TIL) is a heterogeneous lymphocyte group with CD8+ T cells as the main effector cells, exert antitumor roles in TME [31, 32]. The loss of TILs function in TME mainly responsible for the tumor progression and failure of cellular immunotherapy [33, 34]. CD4+ T cells have the dual function both in killing tumor cells directly and in maintaining and promoting CD8+ T cells survival [35]. Considering their importance in antitumor immunity, we concluded that immune-activated patients might be likely to benefit from immunotherapy. In addition, genes that are implicated in immune-related biological processes (such as T cell activation, leukocyte cell-cell adhesion, lymphocyte proliferation, MHC protein complex, cytokine activity, and immune receptor activity) were significantly upregulated in immune-activated patients, indicating that the immunological grouping is reasonable.

Based on an immune-related gene signature (CCR7, CD1B, CD86, CSF2RB, HCK, HLA-DQA1, LTA, LTB, LYZ, NOD2, TNFRSF9, and TNFSF11), a risk-score model was established to evaluate the immune-activated or immunosuppressive status for invasive thyroid carcinoma. The CCR7 chemokine axis not only mediates the trafficking of effector cells that produce an immune response to growing tumors (helping to combat the spread of cancer) but also controls the migration and metastasis of tumor cells to the lymphatic system (contributing to tumor expansion), and therefore, CCR7 has been considered as a tumor therapeutic target [36]. In PTC, CCR7 has been found to show strong correlations with tumor aggressiveness indicators and tumor size [37]. CD1B and CD86 both function in antitumor immunity by mediating the T cell function and NK cell cytotoxicity [38, 39]. CSD2RB has been identified as a recurrent and oncogenic hotspot gain-of-function mutation [40]. HLA-DQA1, a member of MHC Class II molecules, its elevated expression has been identified as an adverse indicator for worse prognosis for esophageal squamous cell carcinoma patients [41]. Loss of NOD2 may promote polarization of tumor-associated macrophages into the M2 phenotype (protumorigenic phenotype) [42]. LTA and LTB encode lymphotoxin alpha and beta proteins, which belong to the tumor necrosis factor (TNF) family together with TNFRSF9 and TNFSF11. Interaction between TNF-TNFR superfamilies can initiate costimulatory signals that play important roles in antitumor immune responses by accelerating the differentiation, survival, and clonal expansion of antigen-primed CD8+ T and CD4+ cells [43]. All of these genes were upregulated in immune-activated samples, suggesting a higher antitumor immunity in these samples. Additionally, several genes were found to be correlated with tumor infiltrating abundance of follicular helper T cells (B cell-help providers) [44], activated NK cells (antitumor cytotoxic lymphocytes) [45], and M1 macrophages (antitumor phenotype) [46], and these cells play important roles in antitumor response. This also confirmed the higher antitumor immunity in immune-activated samples.

We further analyzed the drug sensitivity to identify possible drugs for the treatment of invasive thyroid carcinoma. Bortezomib is an inhibitor for proto-oncogene RET has been approved for treatment of medullary thyroid cancer [47]. MG132 is a proteasome inhibitor that can induce tumor cell apoptosis in thyroid carcinoma [48]. AZD8055 inhibits tumor progression in PTC by affecting mTOR activity [49, 50]. Staurosporine is a bacterial alkaloid, which can inhibit growth of TPC-derived cell line by the synergy effect with rotenone [51]. Immunosuppressive samples showed higher IC50 for bortezomib, MG.132, staurosporine, and AZD8055, suggesting that patients with immunosuppressive status may be resistant to bortezomib, MG.132, staurosporine, and AZD8055 treatments.

High TMB has been proposed as a useful biomarker for predicting the response to immune checkpoint blockade [52]. However, high TMB has also been reported to be failed to predict the response to immune checkpoint blockade in all solid cancer types [53]. In this study, the TMB showed no significant differences between immune-activated and immunosuppressive samples. Among the top 20 mutation genes, mutation in the BRAF gene accounted for 78% of tumor samples, suggesting that BRAF mutation might be correlated with the development of invasive thyroid carcinoma. This was consistent with previous studies. BRAF mutation is the most common mutation in PTC, and tumors with BRAF mutation showed significant correlations with advanced T-stage and more frequently central neck dissection [54]. Genes in the model were not found in the top 20 mutation genes, suggesting that the expression differences of these genes might not be caused by gene mutation.

## 5. Conclusion

We identified a 12-gene-based immune signature, which contributes to predicting the immune-activated or immunosuppressive status for patients with invasive thyroid carcinoma. Patients with immune-activated invasive thyroid carcinoma are more likely to benefit from immunotherapy. Immunosuppressive patients might be resistant to bortezomib, MG.132, Staurosporine, and AZD8055 treatments. These findings will provide clues for improving the clinical management of invasive thyroid carcinoma.

## Data Availability

The dataset supporting the conclusions of this manuscript is included within the manuscript.

## Supplementary Materials

Supplementary Materials Figure S1: functional enrichment analysis for DEG between tumor and normal control. The top 20 enriched biological processes (A), cellular component (B), and molecular function (C) terms in gene ontology annotation; (D) the top 20 enriched KEGG pathways. Figure S2: GSEA for DEGs between immune-activated and immunosuppressive groups. A, the bubble diagram shows the top 20 activated and suppressed pathways in GSEA analysis; B, the top 10 pathways in GSEA ranked by NES value. Table S1: the 29 immune related gene sets. Table S2: immune-related genes obtained from the ImmPort database.
