# Supplementary material for: Development of a Risk Predictive Model for Evaluating Immune Infiltration Status in Invasive Thyroid Carcinoma
Source: Evid Based Complement Alternat Med. 2022 Jun 3;2022:5803077. doi: 10.1155/2022/5803077 (PMC9187459; doi:10.1155/2022/5803077)
Supplement: Supplementary Materials — Figure S1: functional enrichment analysis for DEG between tumor and normal control. The top 20 enriched biological processes (A), cellular component (B), and molecular function (C) terms in gene ontology annotation; (D) the top 20 enriched KEGG pathways. Figure S2: GSEA for DEGs between immune-activated and immunosuppressive groups. A, the bubble diagram shows the top 20 activated and suppressed pathways in GSEA analysis; B, the top 10 pathways in GSEA ranked by NES value. Table S1: the 29 immune related gene sets. Table S2: immune-related genes obtained from the ImmPort database. [file 5803077.f1.zip › 5803077.f1/Table S1.pdf]

aDCs	CD83	LAMP3	CCL1									
APC_co_inhibition	C10orf54	CD274	LGALS9	PDCD1LG2	PVRL3							
APC_co_stimulation	CD40	CD58	CD70	ICOSLG	SLAMF1	TNFSF14	TNFSF15	TNFSF18	TNFSF4	TNFSF8	TNFSF9	
B_cells	BACH2	BANK1	BLK	BTLA	CD79A	CD79B	FCRL1	FCRL3	HVCN1	RALGPS2		
CCR	CCL16	TPO	TGFBR2	CXCL2	CCL14	TGFBR3	IL11RA	CCL11	IL4I1	IL33	CXCL12	CXCL10
CD8+_T_cells	CD8A											
Check-point	IDO1	LAG3	CTLA4	TNFRSF9	ICOS	CD80	PDCD1LG2	TIGIT	CD70	TNFSF9	ICOSLG	KIR3DL1
Cytolytic_activity	PRF1	GZMA										
DCs	CCL17	CCL22	CD209	CCL13								
HLA	HLA-E	HLA-DPB2	HLA-C	HLA-J	HLA-DQB1	HLA-DQB2	HLA-DQA2	HLA-DQA1	HLA-A	HLA-DMA	HLA-DOB	HLA-DRB1
iDCs	CD1A	CD1E										
Inflammation-promoting	CCL5	CD19	CD8B	CXCL10	CXCL13	CXCL9	GNLY	GZMB	IFNG	IL12A	IL12B	IRF1
Macrophages	C11orf45	CD68	CLEC5A	CYBB	FUCA1	GPNMB	HS3ST2	LGMN	MMP9	TM4SF19		
Mast_cells	CMA1	MS4A2	TPSAB1									
MHC_class_I	B2M	HLA-A	TAP1									
Neutrophils	EVI2B	HSD17B11	KDM6B	MEGF9	MNDA	NLRP12	PADI4	SELL	TRANK1	VNN3		
NK_cells	KLRC1	KLRF1										
Parainflammation	CXCL10	PLAT	CCND1	LGMN	PLAUR	AIM2	MMP7	ICAM1	MX2	CXCL9	ANXA1	TLR2
pDCs	CLEC4C	CXCR3	GZMB	IL3RA	IRF7	IRF8	LILRA4	PHEX	PLD4	PTCRA		
T_cell_co-inhibition	BTLA	C10orf54	CD160	CD244	CD274	CTLA4	HAVCR2	LAG3	LAIR1	TIGIT		
T_cell_co-stimulation	CD2	CD226	CD27	CD28	CD40LG	ICOS	SLAMF1	TNFRSF18	TNFRSF25	TNFRSF4	TNFRSF8	TNFRSF9
T_helper_cells	CD4											
Tfh	PDCD1	CXCL13	CXCR5									
Th1_cells	IFNG	TBX21	CTLA4	STAT4	CD38	IL12RB2	LTA	CSF2				
Th2_cells	PMCH	LAIR2	SMAD2	CXCR6	GATA3	IL26						
TIL	ITM2C	CD38	THEMIS2	GLYR1	ICOS	F5	TIGIT	KLRD1	IRF4	PRKCQ	FCRL5	SIRPG
Treg	IL12RB2	TMPRSS6	CTSC	LAPTM4B	TFRC	RNF145	NETO2	ADAT2	CHST2	CTLA4	NFE2L3	LIMA1
Type_I_IFN_Reponse	DDX4	IFIT1	IFIT2	IFIT3	IRF7	ISG20	MX1	MX2	RSAD2	TNFSF10		
Type_II_IFN_Reponse	GPR146	SELP	AHR									

BMPER	BMP8A	CXCL11	IL21R	IL17B	TNFRSF9	ILF2	CX3CR1	CCR8	TNFSF12	CSF3	TNFSF4	BMP3	CX3CL1
CD86	PDCD1	LAIR1	TNFRSF8	TNFSF15	TNFRSF14	IDO2	CD276	CD40	TNFRSF4	TNFSF14	HHLA2	CD244	CD274

HLA-H	HLA-B	HLA-DRB5	HLA-DOA	HLA-DPB1	HLA-DRA	HLA-DRB6	HLA-L	HLA-F	HLA-G	HLA-DMB	HLA-DPA1
PRF1	STAT1	TBX21									

PLA2G2D	ITGA2	MX1	HMOX1	CD276	TIRAP	IL33	PTGES	TNFRSF12A	SCARB1	CD14	BLNK	IFIT3	RETNLB
TNFSF14													

LPXN	IL2RG	CCL5	LCK	TRAF3IP3	CD86	MAL	LILRB1	DOK2	CD6	PAG1	LAX1	PLEK	PIK3CD
IL1R2	ICOS	HSDL2	HTATIP2	FKBP1A	TIGIT	CCR8	LTA	SLC35F2	IL21R	AHCYL1	SOCS2	ETV7	BCL2L1

BMP5	CXCR2	TNFRSF10D	BMP2	CXCL14	CCL28	CXCL3	BMP6	CCL21	CXCL9	CCL23	IL6	TNFRSF18	IL17RD
HAVCR2	CD27	BTLA	LGALS9	TMIGD2	CD28	CD48	TNFRSF25	CD40LG	ADORA2A	VTCN1	CD160	CD44	TNFSF18

IFIT2	ISG15	OAS2	REL	OAS3	CD44	PPARG	BST2	OAS1	NOX1	PLA2G2A	IFIT1	IFITM3	IL1RN

SLAMF1	XCL1	GPR171	XCL2	TBX21	CD2	CD53	KLHL6	SLAMF6	CD40	SIT1	TNFRSF4	CD79A	CD247
RRAGB	ACSL4	CHRNA6	BATF	LAX1	ADPRH	TNFRSF4	ANKRD10	CD274	CASP1	LY75	NPTN	SSTR3	GRSF1

IL17D	IL27	CCL7	IL1R1	CXCR4	CXCR2P1	TGFB1I1	IFNGR1	IL9R	IL1RAPL1	IL11	CSF1	IL20RA	IL25
TNFRSF18	BTNL2	C10orf54	CD200R1	TNFSF4	CD200	NRP1							

LCP2	CD3D	CD27	SH2D1A	FYB	ARHGAP3(	ACAP1	CST7	CD3G	IL2RB	CD3E	FCRL3	CORO1A	ITK
CSF2RB	TMEM184(	NDFIP2	ZBTB38	ERI1	TRAF3	NAB1	HS3ST3B1	LAYN	JAK1	VDR	LEPROT	GCNT1	PTPRJ

TNFRSF4	IL18	ILF3	CCL20	TNFRSF12A	IL6ST	CXCL13	IL12B	TNFRSF8	IL6R	BMPR2	IFNE	IL1RAPL2	IL3RA

TCL1A	CYBB	CSF2RB	IKZF1	NCF4	DOCK2	CCR2	PTPRC	PLAC8	NCKAP1L	IL7R	6-Sep	CD28	STAT4
IKZF2	CSF1	ENTPD1	TNFRSF18	METTL7A	KSR1	SSH1	CADM1	IL1R1	ACP5	CHST7	THADA	CD177	NFAT5

BMP4	CCL24	TNFSF13B	CCR4	IL2RA	IL32	TNFRSF10[illegible]	IL22RA1	BMPR1A	CXCR5	CXCR3	IFNA8	IL17REL	IFNB1

CD8A	LY9	CD48	HCST	PTPRCAP	SASH3	ARHGAP25	LAT	TRAT1	IL10RA	PAX5	CCR7	DOCK11	PARVG
ZNF282	MAGEH1												

IFNAR1	TNFRSF1B	CCL17	IFNL1	IL16	IL1RL1	ILK	CCL25	ILDR2	CXCR1	IL36RN	IL34	TGFB1	IFNG
SPNS1	CD52	HCLS1	ARHGAP9	GIMAP6	PRKCB	MS4A1	GPR18	TBC1D10C	GVINP1	P2RY8	EVI2B	VAMP5	KLRK1

IL19	ILKAP	BMP2K	CCR10	ILDR1	EPO	CCR7	IL17C	IL23A	CCR5	IL7	EPOR	CCL13	IL2RG
SELL	MPEG1	MS4A6A	ARHGAP15	MFNG	GZMK	SELPLG	TARP	GIMAP7	FAM65B	INPP5D	ITGA4	MZB1	GPSM3

IL31RA	TNFAIP6	IFNL2	BMP1	IL12RB1	TNFAIP8	IL4R	TNFRSF6B	TNFAIP8L1	TNFRSF10E	IFNL3	CCL5	CXCL6	CXCL1

STK10	CLEC2D	IL16	NLRC3	GIMAP5	GIMAP4	IFFO1	CFH	PVRIG	CFHR1

CCR3	TNFSF11	CSF1R	IL21	IL1RAP	IL12RB2	CCL1	IL17RA	CCR1	IL1RN	TNFRSF11E	TNFRSF14	IL13	IL2RB

BMP8B	CCL2	IL24	IL18RAP	TGFBI	TNFSF10	TNFRSF11A	CXCL5	IL5RA	TNFSF9	IL1RL2	TNFRSF13C	IL36G	IL15RA

TNFRSF21 CXCL8 IL22RA2 TNFAIP8L2 IL18R1 IFNLR1 CXCR6 CCL3L3 TNFRSF1A IL17RE IFNGR2 IL17RC TNFAIP8L3 ILVBL

TGFBRAP1 CCL4L1 CSF2RA CCRN4L CCL26 TNFAIP1 CCRL2 IFNA10 TNFRSF17 IFNA13 IL20 IL18BP CCL3L1 TNFSF12-T

IL5	IL23R	IL26	TNF	TGFA	CSF2	IL1F10	CXCL17	TNFSF13	IFNA4	IL37	IL12A	IL7R	IFNA1

IL1A	IL4	IL2	CCL22	CSF3R	IL10	IFNK	TGFB2	IL1R2	IL1B	IL17F	IL27RA	IL15	TNFSF8

IL36B	XCL1	CXCL16	TNFRSF19	IL3	CCL3	IFNA2	BMPR1B	IFNA21	TNFSF18	CCL8	IL17RB	TNFRSF25	IL22

IL10RB	IFNAR2	CCL18	IFNA16	CSF2RB	IL36A	TNFAIP3	IL13RA2	IL13RA1	CCR9	TNFRSF10A	IFNA7	IFNW1	XCL2

TNFSF14	CCR2	BMP15	BMP10	CCL15-CCL	TGFBR1	IFNA5	BMP7	IFNA14	IL20RB	IL10RA	IFNA17	CCR6	TGFB3

CCL15	CCL4	CCL27	TNFRSF13E	TNFAIP2	IL31	IL17A	TNFSF15	CCL19	IFNA6	IL9
