# Supplementary material for: Development of a Risk Predictive Model for Evaluating Immune Infiltration Status in Invasive Thyroid Carcinoma
Source: Evid Based Complement Alternat Med. 2022 Jun 3;2022:5803077. doi: 10.1155/2022/5803077 (PMC9187459; doi:10.1155/2022/5803077)
Supplement: Supplementary Materials — Figure S1: functional enrichment analysis for DEG between tumor and normal control. The top 20 enriched biological processes (A), cellular component (B), and molecular function (C) terms in gene ontology annotation; (D) the top 20 enriched KEGG pathways. Figure S2: GSEA for DEGs between immune-activated and immunosuppressive groups. A, the bubble diagram shows the top 20 activated and suppressed pathways in GSEA analysis; B, the top 10 pathways in GSEA ranked by NES value. Table S1: the 29 immune related gene sets. Table S2: immune-related genes obtained from the ImmPort database. [file 5803077.f1.zip › 5803077.f1/Table S2.pdf]

Symbol	ID	Name	Synonyms	Chrom	Category
AZGP1	563	alpha-2-glyco	ZA2G|ZAG	7	Antigen_Processing_and_Presentation
B2M	567	beta-2-microg	IMD43	15	Antigen_Processing_and_Presentation
CALR	811	calreticulin	CRT|HEL-S-99n|RO|	19	Antigen_Processing_and_Presentation
CANX	821	calnexin	CNX|IP90|P90	5	Antigen_Processing_and_Presentation
CD1A	909	CD1a molecule	CD1|FCB6|HTA1|R4|	1	Antigen_Processing_and_Presentation
CD1B	910	CD1b molecule	CD1|CD1A|R1	1	Antigen_Processing_and_Presentation
CD1C	911	CD1c molecule	BDCA1|CD1|CD1A|R7	1	Antigen_Processing_and_Presentation
CD1D	912	CD1d molecule	CD1A|R3|R3G1	1	Antigen_Processing_and_Presentation
CD1E	913	CD1e molecule	CD1A|R2	1	Antigen_Processing_and_Presentation
CD4	920	CD4 molecule	CD4mut	12	Antigen_Processing_and_Presentation
CD8A	925	CD8a molecule	CD8|Leu2|p32	2	Antigen_Processing_and_Presentation
CD8B	926	CD8b molecule	CD8B1|LEU2|LY3|LY	2	Antigen_Processing_and_Presentation
CD74	972	CD74 molecule	DHLAG|HLADG|II|Ia-	5	Antigen_Processing_and_Presentation
CREB1	1385	cAMP responsi	CREB|CREB-1	2	Antigen_Processing_and_Presentation
CTSB	1508	cathepsin B	APPS|CPSB|RECEUP	8	Antigen_Processing_and_Presentation
CTSE	1510	cathepsin E	CATE	1	Antigen_Processing_and_Presentation
CTSL	1514	cathepsin L	CATL|CTSL1|MEP	9	Antigen_Processing_and_Presentation
CTSS	1520	cathepsin S	-	1	Antigen_Processing_and_Presentation
FCER1G	2207	Fc fragment o	FCRG	1	Antigen_Processing_and_Presentation
FCGRT	2217	Fc fragment o	FCRN|alpha-chain	19	Antigen_Processing_and_Presentation
PDIA3	2923	protein disul	ER60|ERp57|ERp60|	15	Antigen_Processing_and_Presentation
HFE	3077	homeostatic i	HFE1|HH|HLA-H|MVC	6	Antigen_Processing_and_Presentation
HLA-A	3105	major histoco	HLAA	6	Antigen_Processing_and_Presentation
HLA-B	3106	major histoco	AS|B-4901|HLAB	6	Antigen_Processing_and_Presentation
HLA-C	3107	major histoco	D6S204|HLA-JY3|HL	6	Antigen_Processing_and_Presentation
HLA-DMA	3108	major histoco	D6S222E|DMA|HLADM	6	Antigen_Processing_and_Presentation
HLA-DMB	3109	major histoco	D6S221E|RING7	6	Antigen_Processing_and_Presentation
HLA-DOA	3111	major histoco	HLA-DNA|HLA-DZA|H	6	Antigen_Processing_and_Presentation
HLA-DOB	3112	major histoco	DOB|HLA_DOB	6	Antigen_Processing_and_Presentation
HLA-DPA1	3113	major histoco	DP(W3)|DP(W4)|DPA	6	Antigen_Processing_and_Presentation
HLA-DPB1	3115	major histoco	DPB1|HLA-DP|HLA-D	6	Antigen_Processing_and_Presentation
HLA-DQA1	3117	major histoco	CELIAC1|DQ-A1|DQA	6	Antigen_Processing_and_Presentation
HLA-DQA2	3118	major histoco	DC-alpha|DX-ALPHA	6	Antigen_Processing_and_Presentation
HLA-DQB1	3119	major histoco	CELIAC1|HLA-DQB|I	6	Antigen_Processing_and_Presentation
HLA-DRA	3122	major histoco	HLA-DRA1	6	Antigen_Processing_and_Presentation
HLA-DRB1	3123	major histoco	DRB1|HLA-DR1B|HLA-	6	Antigen_Processing_and_Presentation
HLA-DRB3	3125	major histoco	DRB3|HLA-DPB1|HLA-	6	Antigen_Processing_and_Presentation
HLA-DRB4	3126	major histoco	DR4|DRB4|HLA-DR4B	6	Antigen_Processing_and_Presentation
HLA-DRB5	3127	major histoco	-	6	Antigen_Processing_and_Presentation
HLA-E	3133	major histoco	HLA-6.2|QA1	6	Antigen_Processing_and_Presentation
HLA-F	3134	major histoco	CDA12|HLA-5.4|HLA-	6	Antigen_Processing_and_Presentation
HLA-G	3135	major histoco	MHC-G	6	Antigen_Processing_and_Presentation
HLA-H	3136	major histoco	HLAHP	6	Antigen_Processing_and_Presentation
MR1	3140	major histoco	HLALS	1	Antigen_Processing_and_Presentation
HSPA1A	3303	heat shock pr	HEL-S-103|HSP70-1	6	Antigen_Processing_and_Presentation
HSPA1B	3304	heat shock pr	HSP70-1|HSP70-1B|	6	Antigen_Processing_and_Presentation
HSPA1L	3305	heat shock pr	HSP70-1L|HSP70-HO	6	Antigen_Processing_and_Presentation
HSPA2	3306	heat shock pr	HSP70-2|HSP70-3	14	Antigen_Processing_and_Presentation
HSPA4	3308	heat shock pr	APG-2|HEL-S-5a|HS	5	Antigen_Processing_and_Presentation
HSPA5	3309	heat shock pr	BIP|GRP78|HEL-S-8	9	Antigen_Processing_and_Presentation
HSPA6	3310	heat shock pr	HSP70B'	1	Antigen_Processing_and_Presentation
HSPA8	3312	heat shock pr	HEL-33|HEL-S-72p|	11	Antigen_Processing_and_Presentation
HSP90AA1	3320	heat shock pr	EL52|HEL-S-65p|HS	14	Antigen_Processing_and_Presentation

HSP90AB1	3326	heat shock pro	D6S182|HSP84|HSP9(	6	Antigen_Processing_and_Presentation
ICAM1	3383	intercellular	BB2|CD54|P3.58	19	Antigen_Processing_and_Presentation
IFNA1	3439	interferon al	IFL|IFN|IFN-ALPHA	9	Antigen_Processing_and_Presentation
IFNA2	3440	interferon al	IFN-alpha-2|IFN-a	9	Antigen_Processing_and_Presentation
IFNA4	3441	interferon al	IFN-alpha4a|INFA4	9	Antigen_Processing_and_Presentation
IFNA5	3442	interferon al	IFN-alpha-5|IFN-a	9	Antigen_Processing_and_Presentation
IFNA6	3443	interferon al	IFN-alphaK	9	Antigen_Processing_and_Presentation
IFNA7	3444	interferon al	IFN-alphaJ|IFNA-J	9	Antigen_Processing_and_Presentation
IFNA8	3445	interferon al	IFN-alphaB	9	Antigen_Processing_and_Presentation
IFNA10	3446	interferon al	IFN-alphaC	9	Antigen_Processing_and_Presentation
IFNA13	3447	interferon al	-	9	Antigen_Processing_and_Presentation
IFNA14	3448	interferon al	IFN-alphaH|LEIF2H	9	Antigen_Processing_and_Presentation
IFNA16	3449	interferon al	IFN-alpha-16|IFN-a	9	Antigen_Processing_and_Presentation
IFNA17	3451	interferon al	IFN-alphaI|IFNA|II	9	Antigen_Processing_and_Presentation
IFNA21	3452	interferon al	IFN-alphaI|LeIF F	9	Antigen_Processing_and_Presentation
IFNG	3458	interferon gai	IFG|IFI	12	Antigen_Processing_and_Presentation
KIR2DL1	3802	killer cell i	CD158A|KIR-K64|KII	19	Antigen_Processing_and_Presentation
KIR2DL2	3803	killer cell i	CD158B1|CD158b|NK	19	Antigen_Processing_and_Presentation
KIR2DL3	3804	killer cell i	CD158B2|CD158b|GL	19	Antigen_Processing_and_Presentation
KIR2DL4	3805	killer cell i	CD158D|G9P|KIR-10	19	Antigen_Processing_and_Presentation
KIR2DS1	3806	killer cell i	CD158H|CD158a|p50.	19	Antigen_Processing_and_Presentation
KIR2DS3	3808	killer cell i	NKAT7	19	Antigen_Processing_and_Presentation
KIR2DS4	3809	killer cell i	CD158I|KIR-2DS4|K	19	Antigen_Processing_and_Presentation
KIR2DS5	3810	killer cell i	CD158G|NKAT9	19	Antigen_Processing_and_Presentation
KIR3DL1	3811	killer cell i	CD158E1|KIR|KIR3DI	19	Antigen_Processing_and_Presentation
KIR3DL2	3812	killer cell i	3DL2|CD158K|KIR-3I	19	Antigen_Processing_and_Presentation
KLRC1	3821	killer cell l	CD159A|NKG2|NKG2A	12	Antigen_Processing_and_Presentation
KLRC2	3822	killer cell l	CD159c|NKG2-C|NKG	12	Antigen_Processing_and_Presentation
KLRC3	3823	killer cell l	NKG2-E|NKG2E	12	Antigen_Processing_and_Presentation
KLRD1	3824	killer cell l	CD94	12	Antigen_Processing_and_Presentation
LTA	4049	lymphotoxin a	LT|TNFB|TNFSF1|TNI	6	Antigen_Processing_and_Presentation
CIITA	4261	class II majo	C2TA|CIITAIV|MHC2	16	Antigen_Processing_and_Presentation
MICA	1E+08	MHC class I p	MIC-A|PERB11.1	6	Antigen_Processing_and_Presentation
MICB	4277	MHC class I p	PERB11.2	6	Antigen_Processing_and_Presentation
NFYA	4800	nuclear trans	CBF-A|CBF-B|HAP2|I	6	Antigen_Processing_and_Presentation
NFYB	4801	nuclear trans	CBF-A|CBF-B|HAP3|I	12	Antigen_Processing_and_Presentation
NFYC	4802	nuclear trans	CBF-C|CBFC|H1TF2A	1	Antigen_Processing_and_Presentation
LGMN	5641	legumain	AEP|LGMN1|PRSC1	14	Antigen_Processing_and_Presentation
PSMB8	5696	proteasome 20	ALDD|D6S216|D6S21	6	Antigen_Processing_and_Presentation
PSMC1	5700	proteasome 26	P26S4|S4|p56	14	Antigen_Processing_and_Presentation
PSMC2	5701	proteasome 26	MSS1|Nbla10058|S7	7	Antigen_Processing_and_Presentation
PSMC3	5702	proteasome 26	TBP1	11	Antigen_Processing_and_Presentation
PSMC4	5704	proteasome 26	MIP224|RPT3|S6|TBI	19	Antigen_Processing_and_Presentation
PSMC5	5705	proteasome 26	S8|SUG-1|SUG1|TBP	17	Antigen_Processing_and_Presentation
PSMC6	5706	proteasome 26	SUG2|p42	14	Antigen_Processing_and_Presentation
PSMD1	5707	proteasome 26	P112|Rpn2|S1	2	Antigen_Processing_and_Presentation
PSMD2	5708	proteasome 26	P97|RPN1|S2|TRAP2	3	Antigen_Processing_and_Presentation
PSMD3	5709	proteasome 26	P58|RPN3|S3|TSTA2	17	Antigen_Processing_and_Presentation
PSMD4	5710	proteasome 26	AF|AF-1|ASF|MCB1|I	1	Antigen_Processing_and_Presentation
PSMD5	5711	proteasome 26	S5B	9	Antigen_Processing_and_Presentation
PSMD7	5713	proteasome 26	MOV34|P40|Rpn8|S1	16	Antigen_Processing_and_Presentation
PSMD8	5714	proteasome 26	HEL-S-91n|HIP6|HYI	19	Antigen_Processing_and_Presentation
PSMD10	5716	proteasome 26	dJ889N15.2|p28|p2	X	Antigen_Processing_and_Presentation
PSMD11	5717	proteasome 26	Rpn6|S9|p44.5	17	Antigen_Processing_and_Presentation

PSMD13	5719	proteasome 26S	HSPC027|Rpn9|S11|p	11	Antigen_Processing_and_Presentation
PSME1	5720	proteasome ac	HEL-S-129m|IFI5111	14	Antigen_Processing_and_Presentation
PSME1	5720	proteasome ac	HEL-S-129m|IFI5111	14	Antigen_Processing_and_Presentation
PSME2	5721	proteasome ac	PA28B|PA28beta|REC	14	Antigen_Processing_and_Presentation
PSME2	5721	proteasome ac	PA28B|PA28beta|REC	14	Antigen_Processing_and_Presentation
RELB	5971	RELB proto-onc	I-REL|IMD53|IREL|I	19	Antigen_Processing_and_Presentation
RFX5	5993	regulatory fac	-	1	Antigen_Processing_and_Presentation
RFXAP	5994	regulatory fac	-	13	Antigen_Processing_and_Presentation
SLC10A2	6555	solute carrier	ASBT|IBAT|ISBT|NTC	13	Antigen_Processing_and_Presentation
TAP1	6890	transporter 1,	ABC17|ABCB2|APT1|I	6	Antigen_Processing_and_Presentation
TAP2	6891	transporter 2,	ABC18|ABCB3|APT2|I	6	Antigen_Processing_and_Presentation
TAPBP	6892	TAP binding pr	NGS17|TAPA|TPN|TPS	6	Antigen_Processing_and_Presentation
THBS1	7057	thrombospondin	THBS|THBS-1|TSP|TS	15	Antigen_Processing_and_Presentation
SEM1	7979	SEM1 26S prote	C7orf76|DSS1|ECD|S	7	Antigen_Processing_and_Presentation
KLRC4	8302	killer cell le	NKG2-F|NKG2F	12	Antigen_Processing_and_Presentation
AP3B1	8546	adaptor relate	ADTB3|ADTB3A|HPS|I	5	Antigen_Processing_and_Presentation
RFXANK	8625	regulatory fac	ANKRA1|BLS|F14150_	19	Antigen_Processing_and_Presentation
PSMD6	9861	proteasome 26S	Rpn7|S10|SGA-113M	3	Antigen_Processing_and_Presentation
PSME3	10197	proteasome ac	HEL-S-283|Ki|PA28-	17	Antigen_Processing_and_Presentation
PSMD14	10213	proteasome 26S	PAD1|POH1|RPN11	2	Antigen_Processing_and_Presentation
CLEC4M	10332	C-type lectin	CD209L|CD299|DC-SI	19	Antigen_Processing_and_Presentation
IFI30	10437	IFI30 lysosoma	GILT|IFI-30|IP-30	19	Antigen_Processing_and_Presentation
PROCR	10544	protein C rece	CCCA|CCD41|EPCR	20	Antigen_Processing_and_Presentation
ADRM1	11047	adhesion regul	ARM-1|ARM1|GP110	20	Antigen_Processing_and_Presentation
ECPAS	23392	Ecm29 proteaso	ECM29|KIAA0368	9	Antigen_Processing_and_Presentation
TRPC4AP	26133	transient rece	C20orf188|PPP1R158	20	Antigen_Processing_and_Presentation
CD209	30835	CD209 molecule	CDSIGN|CLEC4L|DC-S	19	Antigen_Processing_and_Presentation
UBXN1	51035	UBX domain pro	2B28|SAKS1|UBXD10	11	Antigen_Processing_and_Presentation
ERAP1	51752	endoplasmic re	A-LAP|ALAP|APPILS	5	Antigen_Processing_and_Presentation
TAPBPL	55080	TAP binding pr	TAPBP-R|TAPBPR	12	Antigen_Processing_and_Presentation
KIR2DL5A	57292	killer cell im	CD158F|KIR2DL5|KII	19	Antigen_Processing_and_Presentation
ERAP2	64167	endoplasmic re	L-RAP|LRAP	5	Antigen_Processing_and_Presentation
ULBP3	79465	UL16 binding p	N2DL-3|NKG2DL3|RAI	6	Antigen_Processing_and_Presentation
ULBP2	80328	UL16 binding p	ALCAN-alpha|N2DL2	6	Antigen_Processing_and_Presentation
ULBP1	80329	UL16 binding p	N2DL-1|NKG2DL1|RAI	6	Antigen_Processing_and_Presentation
KIR3DL3	1E+05	killer cell im	CD158Z|KIR3DL7|KII	19	Antigen_Processing_and_Presentation
RAET1E	1E+05	retinoic acid	LETAL|N2DL-4|NKG2I	6	Antigen_Processing_and_Presentation
RAET1L	2E+05	retinoic acid	ULBP6	6	Antigen_Processing_and_Presentation
UBR1	2E+05	ubiquitin pro	JBS	15	Antigen_Processing_and_Presentation
RAET1G	4E+05	retinoic acid	ULBP5	6	Antigen_Processing_and_Presentation
PDIA2	64714	protein disul	PDA2|PDI|PDIP|PDII	16	Antigen_Processing_and_Presentation
HAMP	57817	hepcidin antim	HEPC|HFE2B|LEAP1|I	19	Antimicrobials
PI3	5266	peptidase inh	ESI|SKALP|WAP3|WFI	20	Antimicrobials
CAMP	820	cathelicidin a	CAP-18|CAP18|CRAMI	3	Antimicrobials
DEFB4A	1673	defensin beta	BD-2|DEFB-2|DEFB10	8	Antimicrobials
PPBP	5473	pro-platelet b	B-TG1|Beta-TG|CTAI	4	Antimicrobials
REG3G	1E+05	regenerating f	LPPM429|PAP IB|PAI	2	Antimicrobials
CXCL14	9547	C-X-C motif cl	BMAC|BRAK|KEC|KS1	5	Antimicrobials
CXCL16	58191	C-X-C motif cl	CXCLG16|SR-PSOX|SI	17	Antimicrobials
SLPI	6590	secretory leul	ALK1|ALP|BLPI|HUSI	20	Antimicrobials
CXCL8	3576	C-X-C motif cl	GCP-1|GCP1|IL8|LEC	4	Antimicrobials
CXCL10	3627	C-X-C motif cl	C7|IFI10|INP10|IP-	4	Antimicrobials
CXCL9	4283	C-X-C motif cl	CMK|Humig|MIG|SCYI	4	Antimicrobials
CXCL5	6374	C-X-C motif cl	ENA-78|SCYB5	4	Antimicrobials

CXCL11	6373	C-X-C motif cl	H174|I-TAC|IP-9|II	4	Antimicrobials
CXCL6	6372	C-X-C motif cl	CKA-3|GCP-2|GCP2|S	4	Antimicrobials
CXCL1	2919	C-X-C motif cl	FSP|GRO1|GROa|MGSA	4	Antimicrobials
CXCL12	6387	C-X-C motif cl	IRH|PBSF|SCYB12|SI	10	Antimicrobials
CXCL13	10563	C-X-C motif cl	ANGIE|ANGIE2|BCA-1	4	Antimicrobials
CXCL2	2920	C-X-C motif cl	CINC-2a|GRO2|GROb	4	Antimicrobials
PF4	5196	platelet facto	CXCL4|PF-4|SCYB4	4	Antimicrobials
XCL1	6375	X-C motif chem	ATAC|LPTN|LTN|SCM-	1	Antimicrobials
CXCL3	2921	C-X-C motif cl	CINC-2b|GRO3|GROg	4	Antimicrobials
DEFB103B	55894	defensin beta	BD-3|DEFB-3|DEFB10	8	Antimicrobials
CCL13	6357	C-C motif chem	CKb10|MCP-4|NCC-1	17	Antimicrobials
CCL1	6346	C-C motif chem	I-309|P500|SCYA1|S	17	Antimicrobials
DEFB1	1672	defensin beta	BD1|DEFB-1|DEFB101	8	Antimicrobials
CCL8	6355	C-C motif chem	HC14|MCP-2|MCP2|SC	17	Antimicrobials
ELANE	1991	elastase, neu	ELA2|GE|HLE|HNE|NE	19	Antimicrobials
DEFB103A	4E+05	defensin beta	BD-3|DEFB-3|DEFB10	8	Antimicrobials
DEFA3	1668	defensin alpha	DEF3|HNP-3|HNP3|HP	8	Antimicrobials
DEFA1	1667	defensin alpha	DEF1|DEFA2|HNP-1|H	8	Antimicrobials
TMSB10	9168	thymosin beta	MIG12|TB10	2	Antimicrobials
DEFA6	1671	defensin alpha	DEF6|HD-6	8	Antimicrobials
DEFA5	1670	defensin alpha	DEF5|HD-5	8	Antimicrobials
DEFA4	1669	defensin alpha	DEF4|HNP-4|HP-4|HP	8	Antimicrobials
LCN2	3934	lipocalin 2	24p3|MSFI|NGAL|p25	9	Antimicrobials
LCN1	3933	lipocalin 1	PMFA|TLC|TP|VEGP	9	Antimicrobials
COLEC10	10584	collectin sub	3MC3|CL-34|CLL1	8	Antimicrobials
BPI	671	bactericidal p	BPIFD1|rBPI	20	Antimicrobials
S100A9	6280	S100 calcium b	60B8AG|CAGB|CFAG|C	1	Antimicrobials
S100A8	6279	S100 calcium b	60B8AG|CAGA|CFAG|C	1	Antimicrobials
DCD	1E+05	dermcidin	AIDD|DCD-1|DSEP|HC	12	Antimicrobials
LCN6	2E+05	lipocalin 6	LCN5|UNQ643|hLcn5	9	Antimicrobials
S100A12	6283	S100 calcium b	CAAF1|CAGC|CGRP|EN	1	Antimicrobials
HTN3	3347	histatin 3	HIS2|HTN2|HTN5|PB	4	Antimicrobials
LCN8	1E+05	lipocalin 8	EP17|LCN5	9	Antimicrobials
DEFA1B	7E+05	defensin alpha	HNP-1|HP-1|HP1	8	Antimicrobials
CCR10	2826	C-C motif chem	GPR2	17	Antimicrobials
CELA1	1990	chymotrypsin l	ELA1	12	Antimicrobials
DEFB106A	2E+05	defensin beta	BD-6|DEFB-6|DEFB10	8	Antimicrobials
PENK	5179	proenkephalin	PE|PENK-A	8	Antimicrobials
BPIFC	3E+05	BPI fold conta	BPIL2	22	Antimicrobials
MMP12	4321	matrix metallo	HME|ME|MME|MMP-12	11	Antimicrobials
BPIFB6	1E+05	BPI fold conta	BPIL3|LPLUNC6	20	Antimicrobials
LEAP2	1E+05	liver enriched	LEAP-2	5	Antimicrobials
SFTPD	6441	surfactant pro	COLEC7|PSP-D|SFTP4	10	Antimicrobials
LCN9	4E+05	lipocalin 9	HEL129	9	Antimicrobials
BPIFB2	80341	BPI fold conta	BPIL1|C20orf184|LP	20	Antimicrobials
PTGDS	5730	prostaglandin	L-PGDS|LPGDS|PDS|P	9	Antimicrobials
TMSB4X	7114	thymosin beta	FX|PTMB4|TB4X|TMSB	X	Antimicrobials
PGLYRP1	8993	peptidoglycan	PGLYRP|PGRP|PGRP-S	19	Antimicrobials
ZC3HAV1	56829	zinc finger CC	ARTD13|FLB6421|PAR	7	Antimicrobials
TMSB15A	11013	thymosin beta	TMSB15|TMSB15B|TMS	X	Antimicrobials
S100B	6285	S100 calcium b	NEF|S100|S100-B|S1	21	Antimicrobials
S100A13	6284	S100 calcium b	-	1	Antimicrobials
S100A6	6277	S100 calcium b	2A9|5B10|CABP|CACY	1	Antimicrobials
DEFB119	2E+05	defensin beta	DEFB-19|DEFB-20|DE	20	Antimicrobials

DEFB107A	2E+05	defensin beta	BD-7|DEFB-7|DEFB1(	8	Antimicrobials
DEFB105A	2E+05	defensin beta	BD-5|DEFB-5|DEFB1(	8	Antimicrobials
SERPIND1	3053	serpin family	D22S673|HC2|HCF2|I	22	Antimicrobials
DEFB129	1E+05	defensin beta	C20orf87|DEFB-29|I	20	Antimicrobials
DEFB127	1E+05	defensin beta	C20orf73|DEF-27|DI	20	Antimicrobials
S100P	6286	S100 calcium l	MIG9	4	Antimicrobials
S100A7	6278	S100 calcium l	PSOR1|S100A7c	1	Antimicrobials
DEFB104A	1E+05	defensin beta	BD-4|DEFB-4|DEFB1(	8	Antimicrobials
DEFB126	81623	defensin beta	C20orf8|DEFB-26|DI	20	Antimicrobials
DEFB106B	5E+05	defensin beta	BD-6|DEFB-6	8	Antimicrobials
DEFB104B	5E+05	defensin beta	BD-4|DEFB-4|hBD-4	8	Antimicrobials
DEFB107B	5E+05	defensin beta	HsT21816	8	Antimicrobials
PGLYRP3	1E+05	peptidoglycan	PGLYRPIalpha|PGRP-	1	Antimicrobials
PGLYRP2	1E+05	peptidoglycan	HMFT0141|PGLYRPL|I	19	Antimicrobials
S100A10	6281	S100 calcium l	42C|ANX2L|ANX2LG|(	1	Antimicrobials
S100A2	6273	S100 calcium l	CAN19|S100L	1	Antimicrobials
DEFB125	2E+05	defensin beta	DEFB-25	20	Antimicrobials
DEFB123	2E+05	defensin beta	DEFB-23|DEFB23|ES(	20	Antimicrobials
DEFB105B	5E+05	defensin beta	BD-5|DEFB-5	8	Antimicrobials
DEFB132	4E+05	defensin beta	BD-32|DEFB-32|DEFI	20	Antimicrobials
BPIFB3	4E+05	BPI fold conta	C20orf185|LPLUNC3	20	Antimicrobials
LCN12	3E+05	lipocalin 12	-	9	Antimicrobials
PGLYRP4	57115	peptidoglycan	PGLYRPIbeta|PGRP-I	1	Antimicrobials
S100A11	6282	S100 calcium l	HEL-S-43|MLN70|S1(	1	Antimicrobials
S100A5	6276	S100 calcium l	S100D	1	Antimicrobials
S100A3	6274	S100 calcium l	S100E	1	Antimicrobials
S100A1	6271	S100 calcium l	S100|S100-alpha|S1	1	Antimicrobials
DEFB128	2E+05	defensin beta	DEFB-28|DEFB28|hBI	20	Antimicrobials
DEFB108B	2E+05	defensin beta	DEFB-8|hBD-8	11	Antimicrobials
HTN1	3346	histatin 1	HIS1	4	Antimicrobials
LMBR1L	55716	limb developm(	LIMR	12	Antimicrobials
S100A7A	3E+05	S100 calcium l	NICE-2|NICE2|S100/	1	Antimicrobials
DEFB118	1E+05	defensin beta	C20orf63|DEFB-18|I	20	Antimicrobials
COLEC12	81035	collectin sub:	CLP1|NSR2|SCARA4|S	18	Antimicrobials
TMSB4Y	9087	thymosin beta	TB4Y	Y	Antimicrobials
DEFB131A	6E+05	defensin beta	DEFB-31|DEFB131	4	Antimicrobials
DEFB134	6E+05	defensin beta	-	8	Antimicrobials
DEFB130A	2E+05	defensin beta	DEFB-30|DEFB130|DI	8	Antimicrobials
DEFB124	2E+05	defensin beta	DEFB-24	20	Antimicrobials
DEFB121	2E+05	defensin beta	DEFB21|ESC42RELC	20	Antimicrobials
DEFB116	2E+05	defensin beta	DEFB-16	20	Antimicrobials
DEFB115	2E+05	defensin beta	DEFB-15	20	Antimicrobials
DEFB114	2E+05	defensin beta	DEFB-14|DEFB14	6	Antimicrobials
DEFB113	2E+05	defensin beta	DEFB-13	6	Antimicrobials
DEFB112	2E+05	defensin beta	DEFB-12	6	Antimicrobials
DEFB110	2E+05	defensin beta	DEFB-10|DEFB-11|DI	6	Antimicrobials
TMSB15B	3E+05	thymosin beta	TMSB15A|TMSL8|TMSI	X	Antimicrobials
DEFB133	4E+05	defensin beta	-	6	Antimicrobials
S100Z	2E+05	S100 calcium l	Gm625|S100-zeta	5	Antimicrobials
MAVS	57506	mitochondrial	CARDIF|IPS-1|IPS1	20	Antimicrobials
TMSB4XP8	7117	TMSB4X pseudo;	TMSL3	4	Antimicrobials
S100A14	57402	S100 calcium l	BCMP84|S100A15	1	Antimicrobials
LCN10	4E+05	lipocalin 10	-	9	Antimicrobials
S100A16	1E+05	S100 calcium l	AAG13|DT1P1A7|S10(	1	Antimicrobials

DEFB136	6E+05	defensin beta	DEFB137	8	Antimicrobials
DEFB135	6E+05	defensin beta	DEFB136	8	Antimicrobials
DEFB117	2E+05	defensin beta	DEFB-17	20	Antimicrobials
DEFB110	2E+05	defensin beta	DEFB-10|DEFB-11|DI	6	Antimicrobials
ZC3HAV1L	92092	zinc finger C(	C7orf39	7	Antimicrobials
S100A7L2	6E+05	S100 calcium l	S100a7b	1	Antimicrobials
MBL3P	50639	mannose-bindi	COLEC2|MBL	10	Antimicrobials
DEFB4B	1E+08	defensin beta	DEFB4P	8	Antimicrobials
BPIFB4	1E+05	BPI fold conta	C20orf186|LPLUNC4	20	Antimicrobials
IFNAR1	3454	interferon al	AVP|IFN-alpha-REC	21	Antimicrobials
AZU1	566	azurocidin 1	AZAMP|AZU|CAP37|HI	19	Antimicrobials
DEFB131B	1E+08	defensin beta	-	11	Antimicrobials
DEFA1A3	6E+05	defensin alpha	DEFA1|DEFA3|DEFT1I	8	Antimicrobials
LCN1P1	3E+05	lipocalin 1 ps	LCN1L1|bA430N14.2	9	Antimicrobials
S100G	795	S100 calcium l	CABP|CABP1|CABP9K	X	Antimicrobials
DEFA7P	7E+05	defensin alpha	DEFA7	8	Antimicrobials
DEFB130B	1E+08	defensin beta	-	8	Antimicrobials
DEFB108F	1E+08	defensin beta	DEFB108P5	4	Antimicrobials
DEFB131C	1E+08	defensin beta	-	8	Antimicrobials
TCHHL1	1E+05	trichohyalin	S100A17|THHL1	1	Antimicrobials
TINAGL1	64129	tubulointerst	ARG1|LCN7|LIECG3|'	1	Antimicrobials
IFNGR1	3459	interferon gar	CD119|IFNGR|IMD27A	6	Antimicrobials
SLC22A17	51310	solute carrie	24p3R|BOCT|BOIT|N(	14	Antimicrobials
WFIKKN1	1E+05	WAP, follista	C16orf12|RJD2|WFD(	16	Antimicrobials
WFDC2	10406	WAP four-disu	EDDM4|HE4|WAP5|dJ4	20	Antimicrobials
IL6	3569	interleukin 6	BSF-2|BSF2|CDF|HGI	7	Antimicrobials
UMODL1	89766	uromodulin lil	-	21	Antimicrobials
TGFB1	7040	transforming g	CED|DPD1|IBDIMDE|I	19	Antimicrobials
PF4V1	5197	platelet fact(	CXCL4L1|CXCL4V1|PI	4	Antimicrobials
MMP9	4318	matrix metall(	CLG4B|GELB|MANDP2	20	Antimicrobials
ANOS1	3730	anosmin 1	ADMLX|HH1|HHA|KAL	X	Antimicrobials
TLR4	7099	toll like rec(	ARMD10|CD284|TLR-4	9	Antimicrobials
IFNG	3458	interferon gar	IFG|IFI	12	Antimicrobials
SPAG11B	10407	sperm associa	EDDM2B|EP2|EP2C|EI	8	Antimicrobials
A2M	2	alpha-2-macrog	A2MD|CPAMD5|FWP00'	12	Antimicrobials
CTSL	1514	cathepsin L	CATL|CTSL1|MEP	9	Antimicrobials
NFKB1	4790	nuclear facto	CVID12|EBP-1|KBF1	4	Antimicrobials
APOBEC3G	60489	apolipoprotei	A3G|ARCD|ARP-9|ARI	22	Antimicrobials
FABP6	2172	fatty acid bi	I-15P|I-BABP|I-BAI	5	Antimicrobials
NOD2	64127	nucleotide bi	ACUG|BLAU|BLAUS|CA	16	Antimicrobials
MBL2	4153	mannose bindi	COLEC1|HSMBPC|MBL	10	Antimicrobials
SFTPA1	7E+05	surfactant pr(	COLEC4|PSAP|PSP-A	10	Antimicrobials
RBP1	5947	retinol bindi	CRABP-I|CRBP|CRBP1	3	Antimicrobials
TLR2	7097	toll like rec(	CD282|TIL4	4	Antimicrobials
SLC40A1	30061	solute carrie	FPN1|HFE4|IREG1|MS	2	Antimicrobials
PLAU	5328	plasminogen a(	ATF|BDPLT5|QPD|UPA	10	Antimicrobials
IL1B	3553	interleukin 1	IL-1|IL1-BETA|IL1I	2	Antimicrobials
PAEP	5047	progestagen as	GD|GdA|GdF|GdS|PAI	9	Antimicrobials
HJV	1E+05	hemojuvelin BM	HFE2|HFE2A|JH|RGM(	1	Antimicrobials
MUC5AC	4586	mucin 5AC, ol	MUC5|TBM|leB|mucin	11	Antimicrobials
CTSS	1520	cathepsin S	-	1	Antimicrobials
OBP2A	29991	odorant bindi	LCN13|OBP|OBP2C|OI	9	Antimicrobials
PLTP	5360	phospholipid	BPIFE|HDLCQ9	20	Antimicrobials
MX1	4599	MX dynamin lil	IFI-78K|IFI78|MX|M	21	Antimicrobials

DDX58	23586	DExD/H-box he	RIG-I|RIG1|RIGI|RI	9	Antimicrobials
IFNL1	3E+05	interferon la	IL-29|IL29	19	Antimicrobials
IRF3	3661	interferon re	IIAE7	19	Antimicrobials
SFTPA2	7E+05	surfactant pro	COLEC5|PSAP|PSP-A	10	Antimicrobials
LPA	4018	lipoprotein(a)	AK38|APOA|LP	6	Antimicrobials
LBP	3929	lipopolysacch	BPIFD2	20	Antimicrobials
RBP4	5950	retinol bindi	MCOPCB10|RDCCAS	10	Antimicrobials
SFTPA1	7E+05	surfactant pro	COLEC4|PSAP|PSP-A	10	Antimicrobials
NOX4	50507	NADPH oxidase	KOX|KOX-1|RENOX	11	Antimicrobials
LTF	4057	lactotransfer	GIG12|HEL110|HLF2	3	Antimicrobials
IFNB1	3456	interferon be	IFB|IFF|IFN-beta|	9	Antimicrobials
RBP5	83758	retinol bindi	CRBP-III|CRBP3|CRI	12	Antimicrobials
FABP7	2173	fatty acid bi	B-FABP|BLBP|FABPB	6	Antimicrobials
FABP5	2171	fatty acid bi	E-FABP|EFABP|KFABI	8	Antimicrobials
FABP3	2170	fatty acid bi	FABP11|H-FABP|M-F	1	Antimicrobials
FABP2	2169	fatty acid bi	FABPI|I-FABP	4	Antimicrobials
FABP4	2167	fatty acid bi	A-FABP|AFABP|ALBP	8	Antimicrobials
R3HDML	1E+05	R3H domain co	dJ881L22.3	20	Antimicrobials
BPIFA3	1E+05	BPI fold conta	C20orf71|SPLUNC3	20	Antimicrobials
BPIFB1	92747	BPI fold conta	C20orf114|LPLUNC1	20	Antimicrobials
OASL	8638	2'-5'-oligoad	OASL1|OASLd|TRIP-	12	Antimicrobials
CRABP2	1382	cellular reti	CRABP-II|RBP6	1	Antimicrobials
CRABP1	1381	cellular reti	CRABP|CRABP-I|CRAI	15	Antimicrobials
RBP7	1E+05	retinol bindi	CRABP4|CRBP4|CRBP	1	Antimicrobials
DUOX1	53905	dual oxidase	LNOX1|NOXEF1|THOX	15	Antimicrobials
OBP2B	29989	odorant bindi	LCN14|OBPIIb	9	Antimicrobials
RBP2	5948	retinol bindi	CRABP-II|CRBP2|CRI	3	Antimicrobials
LCN15	4E+05	lipocalin 15	PRO6093|UNQ2541	9	Antimicrobials
CETP	1071	cholesteryl e	BPIFF|HDLCQ10	16	Antimicrobials
FABP12	6E+05	fatty acid bi	-	8	Antimicrobials
FABP9	6E+05	fatty acid bi	PERF|PERF15|T-FABI	8	Antimicrobials
BPIFA1	51297	BPI fold conta	LUNX|NASG|PLUNC|SI	20	Antimicrobials
LCNL1	4E+05	lipocalin lik	-	9	Antimicrobials
C8G	733	complement C8	C8C	9	Antimicrobials
SPAG11A	7E+05	sperm associa	EDDM2A|HE2	8	Antimicrobials
PI15	51050	peptidase inh	CRISP8|P24TI|P25T	8	Antimicrobials
NOX1	27035	NADPH oxidase	GP91-2|MOX1|NOH-1	X	Antimicrobials
PMP2	5375	peripheral my	CMT1G|FABP8|M-FABI	8	Antimicrobials
APOD	347	apolipoprotei	-	3	Antimicrobials
ORM2	5005	orosomucoid 2	AGP-B|AGP-B'|AGP2	9	Antimicrobials
ORM1	5004	orosomucoid 1	AGP-A|AGP1|HEL-S-	9	Antimicrobials
TNF	7124	tumor necrosi	DIF|TNF-alpha|TNF	6	Antimicrobials
CTSG	1511	cathepsin G	CATG|CG	14	Antimicrobials
PRTN3	5657	proteinase 3	ACPA|AGP7|C-ANCA|	19	Antimicrobials
MAPK1	5594	mitogen-activ	ERK|ERK-2|ERK2|ER	22	Antimicrobials
PML	5371	PML nuclear b	MYL|PP8675|RNF71|	15	Antimicrobials
AEN	64782	apoptosis enh	ISG20L1|pp12744	15	Antimicrobials
CYBB	1536	cytochrome b-	AMCBX2|CGD|GP91-1	X	Antimicrobials
BPIFA2	1E+05	BPI fold conta	C20orf70|PSP|SPLUI	20	Antimicrobials
ISG20	3669	interferon st	CD25|HEM45	15	Antimicrobials
BCL3	602	BCL3 transcri	BCL4|D19S37	19	Antimicrobials
ISG20L2	81875	interferon st	HSD38	1	Antimicrobials
NOX5	79400	NADPH oxidase	-	15	Antimicrobials
NOX3	50508	NADPH oxidase	GP91-3|MOX-2	6	Antimicrobials

DUOX2	50506	dual oxidase 2	LNOX2|NOXEF2|P138-		15	Antimicrobials
TLR3	7098	toll like rec	CD283|IIAE2		4	Antimicrobials
TFRC	7037	transferrin r	CD71|IMD46|T9|TFR		3	Antimicrobials
IFIH1	64135	interferon in	AGS7|Hlcd|IDDM19|M		2	Antimicrobials
LRP1	4035	LDL receptor	A2MR|APOER|APR|CD9		12	Antimicrobials
TRIM5	85363	tripartite mo	RNF88|TRIM5alpha		11	Antimicrobials
IDO1	3620	indoleamine 2,	IDO|IDO-1|INDO		8	Antimicrobials
GDF15	9518	growth differ	GDF-15|MIC-1|MIC1		19	Antimicrobials
NEDD4	4734	NEDD4 E3 ubiq	NEDD4-1|RPF1		15	Antimicrobials
ADIPOQ	9370	adiponectin, (	ACDC|ACRP30|ADIPQT		3	Antimicrobials
STAT3	6774	signal transdu	ADMIO|ADMIO1|APRF		17	Antimicrobials
STAT1	6772	signal transdu	CANDF7|IMD31A|IMD3		2	Antimicrobials
IFNL2	3E+05	interferon la	IL-28A|IL28A		19	Antimicrobials
SOCS3	9021	suppressor of	ATOD4|CIS3|Cish3|S		17	Antimicrobials
SEMG1	6406	semenogelin 1	CT103|SEMG|SGI|dJ1		20	Antimicrobials
TNFSF10	8743	TNF superfami	APO2L|Apo-2L|CD253		3	Antimicrobials
CCL20	6364	C-C motif che	CKb4|Exodus|LARC|M		2	Antimicrobials
SOCS1	8651	suppressor of	CIS1|CISH1|JAB|SOC		16	Antimicrobials
RNASEL	6041	ribonuclease l	PRCA1|RNS4		1	Antimicrobials
IRF1	3659	interferon re	IRF-1|MAR		5	Antimicrobials
IL15	3600	interleukin 15	IL-15		4	Antimicrobials
APOBEC3F	2E+05	apolipoprotei	A3F|ARP8|BK150C2.4		22	Antimicrobials
PLAAT4	5920	phospholipase	HRASLS4|HRSL4|PLA1		11	Antimicrobials
CHIT1	1118	chitinase 1	CHI3|CHIT|CHITD		1	Antimicrobials
IFNA1	3439	interferon al	IFL|IFN|IFN-ALPHA		9	Antimicrobials
CD40	958	CD40 molecule	Bp50|CDW40|TNFRSF5		20	Antimicrobials
TLR7	51284	toll like rec	TLR7-like	X		Antimicrobials
PPIA	5478	peptidylproly	CYPA|CYPH|HEL-S-69		7	Antimicrobials
HFE	3077	homeostatic i	HFE1|HH|HLA-H|MVCD		6	Antimicrobials
ZYX	7791	zyxin	ESP-2|HED-2		7	Antimicrobials
NLRX1	79671	NLR family me	CLR11.3|DLNB26|NOD		11	Antimicrobials
PGC	5225	progastricsin	PEPC|PGII		6	Antimicrobials
VEGFA	7422	vascular endo	MVCD1|VEGF|VPF		6	Antimicrobials
IKBKE	9641	inhibitor of	IKK-E|IKK-i|IKKE|I		1	Antimicrobials
ISG15	9636	ISG15 ubiquit	G1P2|IFI15|IMD38|I		1	Antimicrobials
DHX58	79132	DExH-box heli	D11LGP2|D11lgp2e|L		17	Antimicrobials
TNFAIP3	7128	TNF alpha ind	A20|AISBL|OTUD7C|T		6	Antimicrobials
TFR2	7036	transferrin r	HFE3|TFRC2		7	Antimicrobials
FCN2	2220	ficolin 2	EBP-37|FCNL|P35|fi		9	Antimicrobials
MUC4	4585	mucin 4, cell	ASGP|HSA276359|MUC		3	Antimicrobials
F2R	2149	coagulation f	CF2R|HTR|PAR-1|PAR		5	Antimicrobials
ELN	2006	elastin	ADCL1|SVAS|WBS|WS		7	Antimicrobials
IL27	2E+05	interleukin 2	IL-27|IL-27A|IL27A		16	Antimicrobials
MAPT	4137	microtubule a	DDPAC|FTDP-17|MAPT		17	Antimicrobials
LYZ	4069	lysozyme	LYZF1|LZM		12	Antimicrobials
CCL5	6352	C-C motif che	D17S136E|RANTES|SC		17	Antimicrobials
LEP	3952	leptin	LEPD|OB|OBS		7	Antimicrobials
CYLD	1540	CYLD lysine 6	BRSS|CDMT|CYLD1|CY		16	Antimicrobials
KLKB1	3818	kallikrein B1	KLK3|PKK|PKKD|PPK		4	Antimicrobials
CST4	1472	cystatin S	-		20	Antimicrobials
CSRP1	1465	cysteine and	CRP|CRP1|CSRP|CYRP		1	Antimicrobials
MAPK14	1432	mitogen-activ	CSBP|CSBP1|CSBP2|C		6	Antimicrobials
JUN	3725	Jun proto-onc	AP-1|AP1|c-Jun|cJU		1	Antimicrobials
ITGAV	3685	integrin subu	CD51|MSK8|VNRA|VTN		2	Antimicrobials

IRF5	3663	interferon re{	SLEB10	7	Antimicrobials
CCR6	1235	C-C motif che	BN-1|C-C CKR-6|CC-	6	Antimicrobials
IL12B	3593	interleukin 1:	CLMF|CLMF2|IL-12B	5	Antimicrobials
TLR8	51311	toll like rec	CD288	X	Antimicrobials
GNLY	10578	granulysin	D2S69E|LAG-2|LAG2	2	Antimicrobials
CD81	975	CD81 molecule	CVID6|S5.7|TAPA1|	11	Antimicrobials
EIF2AK2	5610	eukaryotic tr	EIF2AK1|LEUDEN|PKI	2	Antimicrobials
APOM	55937	apolipoprotei	G3a|HSPC336|NG20|	6	Antimicrobials
CACYBP	27101	calcyclin bin	GIG5|PNAS-107|S10	1	Antimicrobials
NOD1	10392	nucleotide bi	CARD4|CLR7.1|NLRC	7	Antimicrobials
MAPK8	5599	mitogen-activ	JNK|JNK-46|JNK1|J	10	Antimicrobials
MAPK3	5595	mitogen-activ	ERK-1|ERK1|ERT2|H	16	Antimicrobials
BST2	684	bone marrow s	CD317|TETHERIN	19	Antimicrobials
BPHL	670	biphenyl hydr	BPH-RP|MCNAA|VACV	6	Antimicrobials
PLA2G2A	5320	phospholipase	MOM1|PLA2|PLA2B|P	1	Antimicrobials
GRN	2896	granulin prec	CLN11|GEP|GP88|PC	17	Antimicrobials
NEWENTRY	2E+05	-	-	-	Antimicrobials
PDGFRA	5156	platelet deri	CD140A|PDGFR-2|PD	4	Antimicrobials
GNAI1	2770	G protein sub	Gi	7	Antimicrobials
WNT5A	7474	Wnt family me	hWNT5A	3	Antimicrobials
FURIN	5045	furin, paired	FUR|PACE|PCSK3|SP	15	Antimicrobials
ADAR	103	adenosine dea	ADAR1|AGS6|DRADA|I	1	Antimicrobials
TYK2	7297	tyrosine kina	IMD35|JTK1	19	Antimicrobials
NOS2	4843	nitric oxide	HEP-NOS|INOS|NOS|	17	Antimicrobials
TRAF3	7187	TNF receptor	CAP-1|CAP1|CD40bp	14	Antimicrobials
TPT1	7178	tumor protein,	HRF|TCTP|p02|p23	13	Antimicrobials
TPM2	7169	tropomyosin 2	AMCD1|DA1|DA2B|DA	9	Antimicrobials
NEO1	4756	neogenin 1	IGDCC2|NGN|NTN1R2	15	Antimicrobials
AHNAK	79026	AHNAK nucleop	AHNAKRS|PM227	11	Antimicrobials
TLR1	7096	toll like rec	CD281|TIL|TIL. LP	4	Antimicrobials
TK2	7084	thymidine kin	MTDPS2|MTTK|PEOB3	16	Antimicrobials
PRDX2	7001	peroxiredoxin	HEL-S-2a|NKEF-B|N	19	Antimicrobials
MX2	4600	MX dynamin li	MXB	21	Antimicrobials
FGF2	2247	fibroblast gr	BFGF|FGF-2|FGFB|H	4	Antimicrobials
FGA	2243	fibrinogen al	Fib2	4	Antimicrobials
TCF7L2	6934	transcription	TCF-4|TCF4	10	Antimicrobials
F2RL1	2150	F2R like tryp	GPR11|PAR2	5	Antimicrobials
TKFC	26007	triokinase an	DAK|NET45|TKFCD	11	Antimicrobials
MSR1	4481	macrophage sc	CD204|SCARA1|SR-A	8	Antimicrobials
NFKBIZ	64332	NFKB inhibito	IKBZ|INAP|MAIL	3	Antimicrobials
LMBR1	64327	limb developm	ACHP|C7orf2|DIF14	7	Antimicrobials
EPPIN	57119	epididymal pe	CT71|CT72|SPINLW1	20	Antimicrobials
SRC	6714	SRC proto-onc	ASV|SRC1|THC6|c-S	20	Antimicrobials
MPO	4353	myeloperoxida	-	17	Antimicrobials
ELAVL1	1994	ELAV like RNA	ELAV1|HUR|Hua|Mel	19	Antimicrobials
ROBO3	64221	roundabout gu	HGPPS|HGPPS1|HGPS	11	Antimicrobials
SP1	6667	Sp1 transcrip	-	12	Antimicrobials
SOD1	6647	superoxide di	ALS|ALS1|HEL-S-44	21	Antimicrobials
PDF	64146	peptide defor	-	16	Antimicrobials
DLL4	54567	delta like ca	AOS6|delta4|hdelt	15	Antimicrobials
ECD	11319	ecdysoneless	GCR2|HSGT1|SGT1	10	Antimicrobials
SLC11A1	6556	solute carrie	LSH|NRAMP|NRAMP1	2	Antimicrobials
DMBT1	1755	deleted in ma	GP340|SAG|SALSA|m	10	Antimicrobials
STING1	3E+05	stimulator of	ERIS|MITA|MPYS|NE	5	Antimicrobials

SKIV2L	6499	Ski2 like RNA	170A|DDX13|HLP|SKI	6	Antimicrobials
SEMG2	6407	semenogelin 2	SGII	20	Antimicrobials
LTA	4049	lymphotoxin a	LT|TNFB|TNFSF1|TNI	6	Antimicrobials
DES	1674	desmin	CDCD3|CSM1|CSM2|LO	2	Antimicrobials
DCK	1633	deoxycytidine	-	4	Antimicrobials
DAXX	1616	death domain a	BING2|DAP6|EAP1|SM	6	Antimicrobials
TNFRSF10	8797	TNF receptor s	APO2|CD261|DR4|TRA	8	Antimicrobials
TNFRSF10	8795	TNF receptor s	CD262|DR5|KILLER|I	8	Antimicrobials
EED	8726	embryonic ecto	COGIS|HEED|WAIT1	11	Antimicrobials
CCL4	6351	C-C motif chem	ACT2|AT744.1|G-26	17	Antimicrobials
LIMS1	3987	LIM zinc finge	PINCH|PINCH-1|PINC	2	Antimicrobials
LALBA	3906	lactalbumin a	LYZG	12	Antimicrobials
APOBEC3H	2E+05	apolipoprotein	A3H|ARP-10|ARP10	22	Antimicrobials
TMPRSS6	2E+05	transmembrane	IRIDA|MT2	22	Antimicrobials
SPINK5	11005	serine peptida	LEKTI|LETKI|NETS|N	5	Antimicrobials
MARCO	8685	macrophage rec	SCARA2|SR-A6	2	Antimicrobials
BECN1	8678	beclin 1	ATG6|VPS30|beclin1	17	Antimicrobials
TNFSF11	8600	TNF superfami	CD254|ODF|OPGL|OPT	13	Antimicrobials
KNG1	3827	kininogen 1	BDK|BK|HMWK|KNG	3	Antimicrobials
CSK	1445	C-terminal Src	-	15	Antimicrobials
KLRK1	22914	killer cell le	CD314|D12S2489E|KI	12	Antimicrobials
KCNH2	3757	potassium volt	ERG-1|ERG1|H-ERG|I	7	Antimicrobials
JUND	3727	JunD proto-onc	AP-1	19	Antimicrobials
JAK1	3716	Janus kinase 1	JAK1A|JAK1B|JTK3	1	Antimicrobials
CREB1	1385	cAMP responsiv	CREB|CREB-1	2	Antimicrobials
CLDN4	1364	claudin 4	CPE-R|CPER|CPETR|C	7	Antimicrobials
CCL28	56477	C-C motif chem	CCK1|MEC|SCYA28	5	Antimicrobials
RNASE3	6037	ribonuclease A	ECP|RAF1|RNS3	14	Antimicrobials
RN7SL1	6029	RNA component	7L1a|7SL|RN7SL|RNS	14	Antimicrobials
IRF7	3665	interferon reg	IMD39|IRF-7|IRF-7H	11	Antimicrobials
IREB2	3658	iron responsiv	ACO3|IRE-BP 2|IRE-	15	Antimicrobials
ILK	3611	integrin linke	HEL-S-28|ILK-1|ILK	11	Antimicrobials
IL18	3606	interleukin 18	IGIF|IL-18|IL-1g|I	11	Antimicrobials
IL17A	3605	interleukin 17	CTLA-8|CTLA8|IL-17	6	Antimicrobials
LTB4R	1241	leukotriene B4	BLT1|BLTR|CMKRL1|C	14	Antimicrobials
APOBEC3A	2E+05	apolipoprotein	A3A|ARP3|PHRBN|bK1	22	Antimicrobials
MASP2	10747	mannan binding	MAP19|MASP-2|MASP1	1	Antimicrobials
TRIM27	5987	tripartite mot	RFP|RNF76	6	Antimicrobials
RELA	5970	RELA proto-onc	CMCU|NFKB3|p65	11	Antimicrobials
IL7R	3575	interleukin 7	CD127|CDW127|IL-7R	5	Antimicrobials
IL1A	3552	interleukin 1	IL-1 alpha|IL-1A|I	2	Antimicrobials
PTX3	5806	pentraxin 3	TNFAIP5|TSG-14	3	Antimicrobials
IFNAR2	3455	interferon alp	IFN-R|IFN-alpha-RE	21	Antimicrobials
IFN1@	3438	-	IFNA	9	Antimicrobials
SYTL1	84958	synaptotagmin	JFC1|SLP1	1	Antimicrobials
APOBEC3C	27350	apolipoprotein	A3C|APOBEC1L|ARDC2	22	Antimicrobials
DDX17	10521	DEAD-box helic	P72|RH70	22	Antimicrobials
PTGS2	5743	prostaglandin-	COX-2|COX2|GRIPGHS	1	Antimicrobials
HTR1A	3350	5-hydroxytrypt	5-HT-1A|5-HT1A|5HT	5	Antimicrobials
SEPTIN7	989	septin 7	CDC10|CDC3|NBLA029	7	Antimicrobials
CD40LG	959	CD40 ligand	CD154|CD40L|HIGM1	X	Antimicrobials
CD14	929	CD14 molecule	-	5	Antimicrobials
CD8A	925	CD8a molecule	CD8|Leu2|p32	2	Antimicrobials
CD4	920	CD4 molecule	CD4mut	12	Antimicrobials

MASP1	5648	mannan binding	3MC1|CRARF|CRARF1	3	Antimicrobials
PROC	5624	protein C, in	APC|PC|PROC1|THPH	2	Antimicrobials
MAP2K2	5605	mitogen-activ	CFC4|MAPKK2|MEK2|	19	Antimicrobials
MAP2K1	5604	mitogen-activ	CFC3|MAPKK1|MEK1|	15	Antimicrobials
HRG	3273	histidine ric	HPRG|HRGP|THPH11	3	Antimicrobials
NDRG1	10397	N-myc downstr	CAP43|CMT4D|DRG-1	8	Antimicrobials
IRF9	10379	interferon re	IRF-9|ISGF3|ISGF3	14	Antimicrobials
TRIM22	10346	tripartite mo	GPSTAF50|RNF94|ST	11	Antimicrobials
LANCL1	10314	LanC like 1	GPR69A|p40	2	Antimicrobials
PPP4C	5531	protein phosp	PP-X|PP4|PP4C|PPH	16	Antimicrobials
HMOX1	3162	heme oxygenas	HMOX1D|HO-1|HSP32	22	Antimicrobials
HMGB1	3146	high mobility	HMG-1|HMG1|HMG3|S	13	Antimicrobials
HLA-B	3106	major histoco	AS|B-4901|HLAB	6	Antimicrobials
RNASE7	84659	ribonuclease	RAE1	14	Antimicrobials
ABCC4	10257	ATP binding c	MOAT-B|MOATB|MRP4	13	Antimicrobials
HGF	3082	hepatocyte gr	DFNB39|F-TCF|HGFB	7	Antimicrobials
HDAC1	3065	histone deace	GON-10|HD1|KDAC1|	1	Antimicrobials
IFNLR1	2E+05	interferon la	CRF2/12|IFNLR|IL-	1	Antimicrobials
PLSCR1	5359	phospholipid	MMTRA1B	3	Antimicrobials
B2M	567	beta-2-microg	IMD43	15	Antimicrobials
BACH2	60468	BTB domain an	BTBD25|IMD60	6	Antimicrobials
TANK	10010	TRAF family m	I-TRAF|ITRAF|TRAF	2	Antimicrobials
PIK3CG	5294	phosphatidyli	PI3CG|PI3K|PI3Kga	7	Antimicrobials
ARRB1	408	arrestin beta	ARB1|ARR1	11	Antimicrobials
RSAD2	91543	radical S-ade	2510004L01Rik|cig	2	Antimicrobials
STAB2	55576	stabilin 2	FEEL2|FELE-2|FELL	12	Antimicrobials
TBK1	29110	TANK binding	FTDALS4|IIAE8|NAK	12	Antimicrobials
PDYN	5173	prodynorphin	ADCA|PENKB|SCA23	20	Antimicrobials
PDGFRB	5159	platelet deri	CD140B|IBGC4|IMF1	5	Antimicrobials
PDCD1	5133	programmed ce	CD279|PD-1|PD1|SL	2	Antimicrobials
PCSK2	5126	proprotein co	NEC 2|NEC-2|NEC2|	20	Antimicrobials
PCSK1	5122	proprotein co	BMIQ12|NEC1|PC1|P	5	Antimicrobials
ARG2	384	arginase 2	-	14	Antimicrobials
AQP9	366	aquaporin 9	AQP-9|HsT17287|SS	15	Antimicrobials
FASLG	356	Fas ligand	ALPS1B|APT1LG1|AP	1	Antimicrobials
APOH	350	apolipoprotei	B2G1|B2GP1|BG	17	Antimicrobials
BIRC5	332	baculoviral I	API4|EPR-1	17	Antimicrobials
ANXA6	309	annexin A6	ANX6|CBP68|CPB-II	5	Antimicrobials
IL22	50616	interleukin 2	IL-21|IL-22|IL-D1	12	Antimicrobials
VTN	7448	vitronectin	V75|VN|VNT	17	Antimicrobials
VIM	7431	vimentin	-	10	Antimicrobials
VCAM1	7412	vascular cell	CD106|INCAM-100	1	Antimicrobials
PRDX1	5052	peroxiredoxin	MSP23|NKEF-A|NKEF	1	Antimicrobials
GFAP	2670	glial fibrill	ALXDRD	17	Antimicrobials
GBP2	2634	guanylate bin	-	1	Antimicrobials
ALB	213	albumin	HSA|PRO0883|PRO09	4	Antimicrobials
SLC29A3	55315	solute carrie	ENT3|HCLAP|HJCD|P	10	Antimicrobials
OAS1	4938	2'-5'-oligoad	E18/E16|IFI-4|OIA	12	Antimicrobials
AGER	177	advanced glyc	RAGE|SCARJ1	6	Antimicrobials
UNC93B1	81622	unc-93 homolo	IIAE1|UNC93|UNC93	11	Antimicrobials
TNFSF4	7292	TNF superfami	CD134L|CD252|GP34	1	Antimicrobials
NOS1	4842	nitric oxide	IHPS1|N-NOS|NC-NO	12	Antimicrobials
ACTG1	71	actin gamma 1	ACT|ACTG|DFNA20|D	17	Antimicrobials
ACTA1	58	actin alpha 1,	ACTA|ASMA|CFTD|CF	1	Antimicrobials

ACO1	48	aconitase 1	ACONS|HEL60|IREB1	9	Antimicrobials
SERPINA3	12	serpin family	AACT|ACT|GIG24|GI(	14	Antimicrobials
CXCR1	3577	C-X-C motif cl	C-C|C-C-CKR-1|CD1:	2	Antimicrobials
CCL15	6359	C-C motif chei	HCC-2|HMRP-2B|LKN-	17	Antimicrobials
CCL14	6358	C-C motif chei	CC-1|CC-3|CKB1|HC(	17	Antimicrobials
CCL4	6351	C-C motif chei	ACT2|AT744.1|G-26	17	Antimicrobials
CCL16	6360	C-C motif chei	CKb12|HCC-4|ILINCI	17	Antimicrobials
CCL19	6363	C-C motif chei	CKb11|ELC|MIP-3b|M	9	Antimicrobials
CCL13	6357	C-C motif chei	CKb10|MCP-4|NCC-1	17	Antimicrobials
CCL18	6362	C-C motif chei	AMAC-1|AMAC1|CKb7	17	Antimicrobials
CCL17	6361	C-C motif chei	A-152E5.3|ABCD-2|S	16	Antimicrobials
CCL26	10344	C-C motif chei	IMAC|MIP-4a|MIP-4a	7	Antimicrobials
CCL22	6367	C-C motif chei	A-152E5.1|ABCD-1|I	16	Antimicrobials
CCR3	1232	C-C motif chei	C C CKR3|CC-CKR-3	3	Antimicrobials
CCL28	56477	C-C motif chei	CCK1|MEC|SCYA28	5	Antimicrobials
CCL4L1	4E+05	C-C motif chei	AT744.2|CCL4L|LAG-	17	Antimicrobials
ACKR2	1238	atypical chem(	CCBP2|CCR10|CCR9|(	3	Antimicrobials
CCR7	1236	C-C motif chei	BLR2|CC-CKR-7|CCR-	17	Antimicrobials
CCL27	10850	C-C motif chei	ALP|CTACK|CTAK|ESI	9	Antimicrobials
CCR8	1237	C-C motif chei	CC-CKR-8|CCR-8|CD\	3	Antimicrobials
ACKR4	51554	atypical chem(	CC-CKR-11|CCBP2|C(	3	Antimicrobials
CCR10	2826	C-C motif chei	GPR2	17	Antimicrobials
CCL2	6347	C-C motif chei	GDCF-2|HC11|HSMCR:	17	Antimicrobials
CCL21	6366	C-C motif chei	6Ckine|CKb9|ECL|S(	9	Antimicrobials
CCL7	6354	C-C motif chei	FIC|MARC|MCP-3|MCI	17	Antimicrobials
CCL5	6352	C-C motif chei	D17S136E|RANTES|S(	17	Antimicrobials
CCL3	6348	C-C motif chei	G0S19-1|LD78ALPHA	17	Antimicrobials
CCL20	6364	C-C motif chei	CKb4|Exodus|LARC|M	2	Antimicrobials
CCL11	6356	C-C motif chei	SCYA11	17	Antimicrobials
CCR5	1234	C-C motif chei	CC-CKR-5|CCCKR5|C(	3	Antimicrobials
CCL23	6368	C-C motif chei	CK-BETA-8|CKb8|Ckl	17	Antimicrobials
CCL25	6370	C-C motif chei	Ckb15|SCYA25|TECK	19	Antimicrobials
CCL1	6346	C-C motif chei	I-309|P500|SCYA1|S	17	Antimicrobials
CCL3L3	4E+05	C-C motif chei	464.2|D17S1718|G0S	17	Antimicrobials
CCL4L2	9560	C-C motif chei	AT744.2|CCL4L|SCYA	17	Antimicrobials
CXCL12	6387	C-X-C motif cl	IRH|PBSF|SCYB12|SI	10	Antimicrobials
XCL1	6375	X-C motif chei	ATAC|LPTN|LTN|SCM-	1	Antimicrobials
CCL8	6355	C-C motif chei	HC14|MCP-2|MCP2|S(	17	Antimicrobials
CCL3L1	6349	C-C motif chei	464.2|D17S1718|G0S	17	Antimicrobials
CCR1	1230	C-C motif chei	CD191|CKR-1|CKR1|(	3	Antimicrobials
CCL24	6369	C-C motif chei	Ckb-6|MPIF-2|MPIF:	7	Antimicrobials
XCL2	6846	X-C motif chei	SCM-1b|SCM1B|SCYC:	1	Antimicrobials
CXCL1	2919	C-X-C motif cl	FSP|GRO1|GROa|MGSA	4	Antimicrobials
CXCL10	3627	C-X-C motif cl	C7|IFI10|INP10|IP-	4	Antimicrobials
CXCR4	7852	C-X-C motif cl	CD184|D2S201E|FB2:	2	Antimicrobials
CXCL2	2920	C-X-C motif cl	CINC-2a|GRO2|GROb	4	Antimicrobials
CXCR6	10663	C-X-C motif cl	BONZO|CD186|STRL3:	3	Antimicrobials
CCR4	1233	C-C motif chei	CC-CKR-4|CD194|CKI	3	Antimicrobials
CXCL11	6373	C-X-C motif cl	H174|I-TAC|IP-9|II	4	Antimicrobials
TAFA5	25817	TAFA chemokin(	FAM19A5|QLLK5208|T	22	Antimicrobials
TAFA3	3E+05	TAFA chemokin(	FAM19A3|TAFA-3	1	Antimicrobials
TAFA4	2E+05	TAFA chemokin(	FAM19A4|TAFA-4	3	Antimicrobials
TAFA1	4E+05	TAFA chemokin(	FAM19A1|TAFA-1	3	Antimicrobials
TAFA2	3E+05	TAFA chemokin(	FAM19A2|TAFA-2	12	Antimicrobials

CCL15-CC	3E+05	CCL15-CCL14 r	CCL15|HCC-2|LKN-1	17	Antimicrobials
IL6	3569	interleukin 6	BSF-2|BSF2|CDF|HGI	7	Antimicrobials
TNF	7124	tumor necrosi	DIF|TNF-alpha|TNF	6	Antimicrobials
IL1B	3553	interleukin 1	IL-1|IL1-BETA|IL1I	2	Antimicrobials
IL18	3606	interleukin 18	IGIF|IL-18|IL-1g|	11	Antimicrobials
PTK2B	2185	protein tyros	CADTK|CAKB|FADK2|I	8	Antimicrobials
VEGFA	7422	vascular endo	MVCD1|VEGF|VPF	6	Antimicrobials
IL4	3565	interleukin 4	BCGF-1|BCGF1|BSF-	5	Antimicrobials
CDH1	999	cadherin 1	Arc-1|BCDS1|CD324	16	Antimicrobials
CD40	958	CD40 molecule	Bp50|CDW40|TNFRSF	20	Antimicrobials
DEFB103B	55894	defensin beta	BD-3|DEFB-3|DEFB1	8	Antimicrobials
F2RL1	2150	F2R like tryp	GPR11|PAR2	5	Antimicrobials
MMP9	4318	matrix metall	CLG4B|GELB|MANDP2	20	Antimicrobials
LTBP1	4052	latent transf	-	2	Antimicrobials
DEFB4A	1673	defensin beta	BD-2|DEFB-2|DEFB1	8	Antimicrobials
TNFSF10	8743	TNF superfami	APO2L|Apo-2L|CD25	3	Antimicrobials
IL13	3596	interleukin 1	IL-13|P600	5	Antimicrobials
IL10	3586	interleukin 1	CSIF|GVHDS|IL-10|	1	Antimicrobials
IL2	3558	interleukin 2	IL-2|TCGF|lymphok	4	Antimicrobials
PPARG	5468	peroxisome pr	CIMT1|GLM1|NR1C3|I	3	Antimicrobials
FGR	2268	FGR proto-onc	SRC2|c-fgr|c-src2	1	Antimicrobials
MIF	4282	macrophage mi	GIF|GLIF|MMIF	22	Antimicrobials
CRP	1401	C-reactive pr	PTX1	1	Antimicrobials
JAK2	3717	Janus kinase	JTK10|THCYT3	9	Antimicrobials
IL1A	3552	interleukin 1	IL-1 alpha|IL-1A|	2	Antimicrobials
PTK2	5747	protein tyros	FADK|FAK|FAK1|FRNI	8	Antimicrobials
PTGDR	5729	prostaglandin	AS1|ASRT1|DP|DP1|I	14	Antimicrobials
CD86	942	CD86 molecule	B7-2|B7.2|B70|CD2	3	Antimicrobials
HCK	3055	HCK proto-onc	JTK9|p59Hck|p61Hcl	20	Antimicrobials
ARRB1	408	arrestin beta	ARB1|ARR1	11	Antimicrobials
GNAI1	2770	G protein sub	Gi	7	Antimicrobials
VDR	7421	vitamin D rec	NR1I1|PPP1R163	12	Antimicrobials
OLR1	4973	oxidized low	CLEC8A|LOX1|LOXIN	12	Antimicrobials
GRK2	156	G protein-cou	ADRBK1|BARK1|BETA-	11	Antimicrobials
TXK	7294	TXK tyrosine	BTKL|PSCTK5|PTK4|I	4	Antimicrobials
RNASE2	6036	ribonuclease	EDN|RAF3|RNS2	14	Antimicrobials
CD79A	973	CD79a molecul	IGA|MB-1	19	BCRSignalingPathway
CD79B	974	CD79b molecul	AGM6|B29|IGB	17	BCRSignalingPathway
LYN	4067	LYN proto-onc	JTK8|p53Lyn|p56Ly	8	BCRSignalingPathway
SYK	6850	spleen associ	p72-Syk	9	BCRSignalingPathway
BTK	695	Bruton tyrosi	AGMX1|AT|ATK|BPK|	X	BCRSignalingPathway
BLNK	29760	B cell linker	AGM4|BASH|BLNK-S|I	10	BCRSignalingPathway
VAV3	10451	vav guanine n	-	1	BCRSignalingPathway
VAV1	7409	vav guanine n	VAV	19	BCRSignalingPathway
VAV2	7410	vav guanine n	VAV-2	9	BCRSignalingPathway
RAC1	5879	Rac family sm	MIG5|MRD48|Rac-1|	7	BCRSignalingPathway
RAC2	5880	Rac family sm	EN-7|Gx|HSPC022|p	22	BCRSignalingPathway
RAC3	5881	Rac family sm	-	17	BCRSignalingPathway
PPP3CA	5530	protein phosp	ACCIID|CALN|CALNA	4	BCRSignalingPathway
PPP3CB	5532	protein phosp	CALNA2|CALNB|CNA2	10	BCRSignalingPathway
PPP3CC	5533	protein phosp	CALNA3|CNA3|PP2Bg	8	BCRSignalingPathway
CHP1	11261	calcineurin 1	CHP|SLC9A1BP|SPAX	15	BCRSignalingPathway
PPP3R1	5534	protein phosp	CALNB1|CNB|CNB1	2	BCRSignalingPathway
PPP3R2	5535	protein phosp	PPP3RL	9	BCRSignalingPathway

CHP2	63928	calcineurin l	-	16	BCRSignalingPathway
NFAT5	10725	nuclear facto	NF-AT5|NFATL1|NFA	16	BCRSignalingPathway
NFATC1	4772	nuclear facto	NF-ATC|NF-ATc1.2|N	18	BCRSignalingPathway
NFATC2	4773	nuclear facto	NFAT1|NFATP	20	BCRSignalingPathway
NFATC3	4775	nuclear facto	NF-AT4c|NFAT4|NFA	16	BCRSignalingPathway
NFATC4	4776	nuclear facto	NF-AT3|NF-ATC4|NF	14	BCRSignalingPathway
HRAS	3265	HRas proto-onc	C-BAS/HAS|C-H-RAS	11	BCRSignalingPathway
KRAS	3845	KRAS proto-onc	'C-K-RAS|C-K-RAS|C	12	BCRSignalingPathway
NRAS	4893	NRAS proto-onc	ALPS4|CMNS|N-ras|N	1	BCRSignalingPathway
FOS	2353	Fos proto-onco	AP-1|C-FOS|p55	14	BCRSignalingPathway
JUN	3725	Jun proto-onco	AP-1|AP1|c-Jun|cJU	1	BCRSignalingPathway
CARD11	84433	caspase recru	BENTA|BIMP3|CARMA	7	BCRSignalingPathway
BCL10	8915	BCL10 immune s	CARMEN|CIPER|CLAP	1	BCRSignalingPathway
MALT1	10892	MALT1 paracasp	IMD12|MLT|MLT1|PCA	18	BCRSignalingPathway
CHUK	1147	component of	IKBKA|IKK-alpha|IK	10	BCRSignalingPathway
IKBKB	3551	inhibitor of	IKK-beta|IKK2|IKKB	8	BCRSignalingPathway
IKBKG	8517	inhibitor of	AMCBX1|EDAID1|FIP-X		BCRSignalingPathway
NFKB1	4790	nuclear facto	CVID12|EBP-1|KBF1	4	BCRSignalingPathway
RELA	5970	RELA proto-onc	CMCU|NFKB3|p65	11	BCRSignalingPathway
NFKBIA	4792	NFKB inhibito	EDAID2|IKBA|MAD-3	14	BCRSignalingPathway
NFKBIB	4793	NFKB inhibito	IKBB|TRIP9	19	BCRSignalingPathway
NFKBIE	4794	NFKB inhibito	IKBE	6	BCRSignalingPathway
CD81	975	CD81 molecule	CVID6|S5.7|TAPA1|T	11	BCRSignalingPathway
CD19	930	CD19 molecule	B4|CVID3	16	BCRSignalingPathway
CR2	1380	complement C3	C3DR|CD21|CR|CVID	1	BCRSignalingPathway
PIK3R5	23533	phosphoinosit	F730038I15Rik|FOAP	17	BCRSignalingPathway
PIK3R1	5295	phosphoinosit	AGM7|GRB1|IMD36|p8	5	BCRSignalingPathway
PIK3R2	5296	phosphoinosit	MPPH|MPPH1|P85B|p8	19	BCRSignalingPathway
PIK3R3	8503	phosphoinosit	p55|p55-GAMMA|p55P	1	BCRSignalingPathway
PIK3CA	5290	phosphatidyli	CLAPO|CLOVE|CWS5|M	3	BCRSignalingPathway
PIK3CB	5291	phosphatidyli	P110BETA|PI3K|PI3K	3	BCRSignalingPathway
PIK3CD	5293	phosphatidyli	APDS|IMD14|P110DEL	1	BCRSignalingPathway
PIK3CG	5294	phosphatidyli	PI3CG|PI3K|PI3Kgam	7	BCRSignalingPathway
AKT3	10000	AKT serine/th	MPPH|MPPH2|PKB-GAM	1	BCRSignalingPathway
AKT1	207	AKT serine/th	AKT|CWS6|PKB|PKB-A	14	BCRSignalingPathway
AKT2	208	AKT serine/th	HIHGHH|PKBB|PKBBET	19	BCRSignalingPathway
GSK3B	2932	glycogen synth	-	3	BCRSignalingPathway
INPP5D	3635	inositol poly	SHIP|SHIP-1|SHIP1	2	BCRSignalingPathway
CD22	933	CD22 molecule	SIGLEC-2|SIGLEC2	19	BCRSignalingPathway
CD72	971	CD72 molecule	CD72b|LYB2	9	BCRSignalingPathway
PTPN6	5777	protein tyros	HCP|HCPH|HPTP1C|PT	12	BCRSignalingPathway
LILRB3	11025	leukocyte imm	CD85A|HL9|ILT-5|IL	19	BCRSignalingPathway
FCGR2B	2213	Fc fragment o	CD32|CD32B|FCG2|FC	1	BCRSignalingPathway
RASGRP3	25780	RAS guanyl re	GRP3	2	BCRSignalingPathway
PLCG2	5336	phospholipase	APLAID|FCAS3|PLC-I	16	BCRSignalingPathway
PRKCB	5579	protein kinas	PKC-beta|PKCB|PKCI	16	BCRSignalingPathway
IFITM1	8519	interferon in	9-27|CD225|DSPA2a	11	BCRSignalingPathway
IGH	3492	immunoglobuli	IGD1|IGH.1@|IGH@|I	14	BCRSignalingPathway
IGHA1	3493	immunoglobuli	IgA1	14	BCRSignalingPathway
IGHA2	3494	immunoglobuli	-	14	BCRSignalingPathway
IGHD	3495	immunoglobuli	-	14	BCRSignalingPathway
IGHD1-1	28510	immunoglobuli	IGHD11	14	BCRSignalingPathway
IGHD1-14	28508	immunoglobuli	DM2|IGHD114	14	BCRSignalingPathway
IGHD1-20	28507	immunoglobuli	IGHD120	14	BCRSignalingPathway

IGHD1-26	28506	immunoglobuli	IGHD126	14	BCRSignalingPathway
IGHD1-7	28509	immunoglobuli	DM1|IGHD17	14	BCRSignalingPathway
IGHD2-15	28503	immunoglobuli	D2|IGHD215	14	BCRSignalingPathway
IGHD2-2	28505	immunoglobuli	IGHD22	14	BCRSignalingPathway
IGHD2-21	28502	immunoglobuli	IGHD221	14	BCRSignalingPathway
IGHD2-8	28504	immunoglobuli	DLR1|IGHD28	14	BCRSignalingPathway
IGHD3-10	28499	immunoglobuli	DXP'1|IGHD310	14	BCRSignalingPathway
IGHD3-16	28498	immunoglobuli	IGHD316	14	BCRSignalingPathway
IGHD3-22	28497	immunoglobuli	IGHD322	14	BCRSignalingPathway
IGHD3-3	28501	immunoglobuli	DXP4|IGHD33	14	BCRSignalingPathway
IGHD3-9	28500	immunoglobuli	DXP1|IGHD39	14	BCRSignalingPathway
IGHD4-11	28495	immunoglobuli	DA1|IGHD411	14	BCRSignalingPathway
IGHD4-17	28494	immunoglobuli	IGHD417	14	BCRSignalingPathway
IGHD4-23	28493	immunoglobuli	IGHD423	14	BCRSignalingPathway
IGHD4-4	28496	immunoglobuli	DA4|IGHD44	14	BCRSignalingPathway
IGHD5-12	28491	immunoglobuli	DK1|IGHD512	14	BCRSignalingPathway
IGHD5-18	28490	immunoglobuli	IGHD518	14	BCRSignalingPathway
IGHD5-24	28489	immunoglobuli	IGHD524	14	BCRSignalingPathway
IGHD5-5	28492	immunoglobuli	DK4|IGHD55	14	BCRSignalingPathway
IGHD6-13	28487	immunoglobuli	DN1|IGHD613	14	BCRSignalingPathway
IGHD6-19	28486	immunoglobuli	IGHD619	14	BCRSignalingPathway
IGHD6-25	28485	immunoglobuli	IGHD625	14	BCRSignalingPathway
IGHD6-6	28488	immunoglobuli	D(N4)|IGHD66	14	BCRSignalingPathway
IGHD7-27	28484	immunoglobuli	DHQ52|IGHD727	14	BCRSignalingPathway
IGHE	3497	immunoglobuli	IgE	14	BCRSignalingPathway
IGHG1	3500	immunoglobuli	-	14	BCRSignalingPathway
IGHG2	3501	immunoglobuli	-	14	BCRSignalingPathway
IGHG3	3502	immunoglobuli	IgG3	14	BCRSignalingPathway
IGHG4	3503	immunoglobuli	-	14	BCRSignalingPathway
IGHJ1	28483	immunoglobuli	JH1	14	BCRSignalingPathway
IGHJ2	28481	immunoglobuli	JH2	14	BCRSignalingPathway
IGHJ3	28479	immunoglobuli	JH3b	14	BCRSignalingPathway
IGHJ4	28477	immunoglobuli	JH4b	14	BCRSignalingPathway
IGHJ5	28476	immunoglobuli	JH5b	14	BCRSignalingPathway
IGHJ6	28475	immunoglobuli	JH6b	14	BCRSignalingPathway
IGHM	3507	immunoglobuli	AGM1|MU|VH	14	BCRSignalingPathway
IGH	3492	immunoglobuli	IGD1|IGH.1@|IGH@|	14	BCRSignalingPathway
IGHV1-18	28468	immunoglobuli	IGHV118	14	BCRSignalingPathway
IGHV1-2	28474	immunoglobuli	IGHV12|V35	14	BCRSignalingPathway
IGHV1-24	28467	immunoglobuli	IGHV124|VH	14	BCRSignalingPathway
IGHV1-3	28473	immunoglobuli	IGHV13|VI-3B	14	BCRSignalingPathway
IGHV1-45	28466	immunoglobuli	IGHV145|VH	14	BCRSignalingPathway
IGHV1-46	28465	immunoglobuli	IGHV146	14	BCRSignalingPathway
IGHV1-58	28464	immunoglobuli	IGHV158|VH	14	BCRSignalingPathway
IGHV1-69	28461	immunoglobuli	IGHV1-E|IGHV169|I	14	BCRSignalingPathway
IGHV1-8	28472	immunoglobuli	IGHV18	14	BCRSignalingPathway
IGHV1-38	28460	immunoglobuli	IGHV1-C|IGHV1C	14	BCRSignalingPathway
IGHV1-69	28458	immunoglobuli	IGHV1-F|IGHV1F	14	BCRSignalingPathway
IGHV2-26	28455	immunoglobuli	IGHV226|VH	14	BCRSignalingPathway
IGHV2-5	28457	immunoglobuli	IGHV25|VH	14	BCRSignalingPathway
IGHV2-70	28454	immunoglobuli	IGHV270|VH	14	BCRSignalingPathway
IGHV3-11	28450	immunoglobuli	IGHV311|VH	14	BCRSignalingPathway
IGHV3-13	28449	immunoglobuli	IGHV313	14	BCRSignalingPathway
IGHV3-15	28448	immunoglobuli	IGHV315|VH	14	BCRSignalingPathway

IGHV3-16	28447	immunoglobuli	IGHV316|VH	14	BCRSignalingPathway
IGHV3-20	28445	immunoglobuli	IGHV320|VH	14	BCRSignalingPathway
IGHV3-21	28444	immunoglobuli	IGHV321|VH	14	BCRSignalingPathway
IGHV3-23	28442	immunoglobuli	DP47|IGHV323|V3-2:	14	BCRSignalingPathway
IGHV3-30	28439	immunoglobuli	IGHV330|VH	14	BCRSignalingPathway
IGHV3-30	57290	immunoglobuli	IGHV3-3|IGHV3303	14	BCRSignalingPathway
IGHV3-30	89770	immunoglobuli	IGHV3-3|IGHV3305	14	BCRSignalingPathway
IGHV3-33	28434	immunoglobuli	IGHV333|VH	14	BCRSignalingPathway
IGHV3-35	28432	immunoglobuli	IGHV335|VH	14	BCRSignalingPathway
IGHV3-38	28429	immunoglobuli	IGHV338|VH	14	BCRSignalingPathway
IGHV3-43	28426	immunoglobuli	IGHV343|VH	14	BCRSignalingPathway
IGHV3-48	28424	immunoglobuli	IGHV348|VH	14	BCRSignalingPathway
IGHV3-49	28423	immunoglobuli	IGHV349|VH	14	BCRSignalingPathway
IGHV3-53	28420	immunoglobuli	IGHV353|VH	14	BCRSignalingPathway
IGHV3-64	28414	immunoglobuli	IGHV364|VH	14	BCRSignalingPathway
IGHV3-66	28412	immunoglobuli	IGHV366|VH	14	BCRSignalingPathway
IGHV3-7	28452	immunoglobuli	IGHV37|VH	14	BCRSignalingPathway
IGHV3-72	28410	immunoglobuli	IGHV372|VH	14	BCRSignalingPathway
IGHV3-73	28409	immunoglobuli	IGHV373|VH	14	BCRSignalingPathway
IGHV3-74	28408	immunoglobuli	IGHV374|VH	14	BCRSignalingPathway
IGHV3-9	28451	immunoglobuli	IGHV39|VH	14	BCRSignalingPathway
IGHV3-38	28404	immunoglobuli	IGHV3-D|IGHV3D	14	BCRSignalingPathway
IGHV3-69	28402	immunoglobuli	IGH|IGHM|IGHV|IGH	14	BCRSignalingPathway
IGHV4-28	28400	immunoglobuli	IGHV428|VH	14	BCRSignalingPathway
IGHV4-30	28399	immunoglobuli	IGHV4-3	14	BCRSignalingPathway
IGHV4-30	28398	immunoglobuli	IGHV4-3|IGHV4302	14	BCRSignalingPathway
IGHV4-30	28397	immunoglobuli	IGHV4-3|IGHV4304	14	BCRSignalingPathway
IGHV4-31	28396	immunoglobuli	IGHV431	14	BCRSignalingPathway
IGHV4-34	28395	immunoglobuli	IGHV434|VH	14	BCRSignalingPathway
IGHV4-39	28394	immunoglobuli	IGHV439|VH	14	BCRSignalingPathway
IGHV4-4	28401	immunoglobuli	IGHV44|VH	14	BCRSignalingPathway
IGHV4-59	28392	immunoglobuli	IGHV459|VH	14	BCRSignalingPathway
IGHV4-61	28391	immunoglobuli	IGHV461|VH	14	BCRSignalingPathway
IGHV4-38	28389	immunoglobuli	IGHV4-B|IGHV4B	14	BCRSignalingPathway
IGHV5-51	28388	immunoglobuli	IGHV551|VH	14	BCRSignalingPathway
IGHV5-10	28386	immunoglobuli	IGHV5-A|IGHV5A	14	BCRSignalingPathway
IGHV6-1	28385	immunoglobuli	IGHV61|VH	14	BCRSignalingPathway
IGHV7-4-	57289	immunoglobuli	IGHV7-41|IGHV741	14	BCRSignalingPathway
IGHV7-81	28378	immunoglobuli	IGHV781	14	BCRSignalingPathway
IGK	50802	immunoglobuli	IGK@	2	BCRSignalingPathway
IGKC	3514	immunoglobuli	HCAK1|IGKCD|Km	2	BCRSignalingPathway
IGKDEL	3515	immunoglobuli	IGKDE	2	BCRSignalingPathway
IGKJ	7842	-	IGKJ@	2	BCRSignalingPathway
IGKJ1	28950	immunoglobuli	J1	2	BCRSignalingPathway
IGKJ2	28949	immunoglobuli	J2	2	BCRSignalingPathway
IGKJ3	28948	immunoglobuli	J3	2	BCRSignalingPathway
IGKJ4	28947	immunoglobuli	J4	2	BCRSignalingPathway
IGKJ5	28946	immunoglobuli	J5	2	BCRSignalingPathway
IGKV@	3519	-	IGKV|IGKV1|IGKV1@	2	BCRSignalingPathway
IGKV1-12	28940	immunoglobuli	IGKV112|L19	2	BCRSignalingPathway
IGKV1-13	28939	immunoglobuli	IGKV113|L18	2	BCRSignalingPathway
IGKV1-16	28938	immunoglobuli	IGKV116|L1	2	BCRSignalingPathway
IGKV1-17	28937	immunoglobuli	A30|IGKV117	2	BCRSignalingPathway
IGKV1-27	28935	immunoglobuli	A20|IGKV127	2	BCRSignalingPathway

IGKV1-33	28933	immunoglobuli	IGKV133|O18	2	BCRSignalingPathway
IGKV1-37	28931	immunoglobuli	IGKV137|O14	2	BCRSignalingPathway
IGKV1-39	28930	immunoglobuli	IGKV139|O12|O12a	2	BCRSignalingPathway
IGKV1-5	28299	immunoglobuli	IGKV|IGKV15|L12|L	2	BCRSignalingPathway
IGKV1-6	28943	immunoglobuli	IGKV16|L11	2	BCRSignalingPathway
IGKV1-8	28942	immunoglobuli	IGKV18|L9	2	BCRSignalingPathway
IGKV1-9	28941	immunoglobuli	IGKV19|L8	2	BCRSignalingPathway
IGKV1D-1	28903	immunoglobuli	IGKV1D12|L19	2	BCRSignalingPathway
IGKV1D-1	28902	immunoglobuli	IGKV1D13|L18	2	BCRSignalingPathway
IGKV1D-1	28901	immunoglobuli	IGKV1D16|L15|L15a	2	BCRSignalingPathway
IGKV1D-1	28900	immunoglobuli	IGKV1D17|L14	2	BCRSignalingPathway
IGKV1D-3	28896	immunoglobuli	IGKV1D33|O8	2	BCRSignalingPathway
IGKV1D-3	28894	immunoglobuli	IGKV1D37|O4	2	BCRSignalingPathway
IGKV1D-3	28893	immunoglobuli	IGKV1D39|O2	2	BCRSignalingPathway
IGKV1D-4	28892	immunoglobuli	IGKV1D42|L22	2	BCRSignalingPathway
IGKV1D-4	28891	immunoglobuli	IGKV1D43|L23|L23a	2	BCRSignalingPathway
IGKV1D-8	28904	immunoglobuli	IGKV1D8|L24|L24a	2	BCRSignalingPathway
IGKV2-24	28923	immunoglobuli	A23|IGKV224	2	BCRSignalingPathway
IGKV2-28	28921	immunoglobuli	A19|IGKV228	2	BCRSignalingPathway
IGKV2-30	28919	immunoglobuli	A17|IGKV230	2	BCRSignalingPathway
IGKV2-40	28916	immunoglobuli	IGKV240|O11|O11a	2	BCRSignalingPathway
IGKV2D-2	28885	immunoglobuli	A7|IGKV2D24	2	BCRSignalingPathway
IGKV2D-2	28883	immunoglobuli	A3|IGKV2D28	2	BCRSignalingPathway
IGKV2D-2	28882	immunoglobuli	A2a|A2c|IGKV2D29	2	BCRSignalingPathway
IGKV2D-3	28881	immunoglobuli	A1|IGKV2D30	2	BCRSignalingPathway
IGKV2D-4	28878	immunoglobuli	IGKV2D40|O1	2	BCRSignalingPathway
IGKV3-11	28914	immunoglobuli	IGKV311|L6	2	BCRSignalingPathway
IGKV3-15	28913	immunoglobuli	IGKV315|L2	2	BCRSignalingPathway
IGKV3-20	28912	immunoglobuli	13K18|A27|IGKV320	2	BCRSignalingPathway
IGKV3-7	28915	immunoglobuli	IGKV37|L10|L10a|Vl	2	BCRSignalingPathway
IGKV3D-1	28876	immunoglobuli	IGKV3D11|L20	2	BCRSignalingPathway
IGKV3D-1	28875	immunoglobuli	IGKV3D15|L16|L16a	2	BCRSignalingPathway
IGKV3D-2	28874	immunoglobuli	A11|A11a|IGKV3D20	2	BCRSignalingPathway
IGKV3D-7	28877	immunoglobuli	IGKV3D7|L25	2	BCRSignalingPathway
IGKV4-1	28908	immunoglobuli	B3|IGKV41	2	BCRSignalingPathway
IGKV5-2	28907	immunoglobuli	B2|IGKV52	2	BCRSignalingPathway
IGKV6-21	28906	immunoglobuli	A26|IGKV621	2	BCRSignalingPathway
IGKV6D-2	28870	immunoglobuli	A10|IGKV6D21	2	BCRSignalingPathway
IGKV6D-4	28869	immunoglobuli	A14	2	BCRSignalingPathway
IGL	3535	immunoglobuli	IGL@|IGLC6	22	BCRSignalingPathway
IGLC1	3537	immunoglobuli	IGLC	22	BCRSignalingPathway
IGLC2	3538	immunoglobuli	IGLC	22	BCRSignalingPathway
IGLC3	3539	immunoglobuli	IGLC	22	BCRSignalingPathway
IGLC6	3542	immunoglobuli	IGLC	22	BCRSignalingPathway
IGLC7	28834	immunoglobuli	C7	22	BCRSignalingPathway
IGLJ	8217	-	IGLJ@	22	BCRSignalingPathway
IGLJ1	28833	immunoglobuli	J1	22	BCRSignalingPathway
IGLJ2	28832	immunoglobuli	J2	22	BCRSignalingPathway
IGLJ3	28831	immunoglobuli	J3	22	BCRSignalingPathway
IGLJ4	28830	immunoglobuli	-	22	BCRSignalingPathway
IGLJ5	28829	immunoglobuli	-	22	BCRSignalingPathway
IGLJ6	28828	immunoglobuli	-	22	BCRSignalingPathway
IGLJ7	28827	immunoglobuli	J7	22	BCRSignalingPathway
IGLV@	3546	-	IGLV	22	BCRSignalingPathway

IGLV1-36	28826	immunoglobuli	IGLV136|V1-11	22	BCRSignalingPathway
IGLV1-40	28825	immunoglobuli	IGLV140|V1-13	22	BCRSignalingPathway
IGLV1-44	28823	immunoglobuli	IGLV144|V1-16	22	BCRSignalingPathway
IGLV1-47	28822	immunoglobuli	IGLV147|V1-17	22	BCRSignalingPathway
IGLV1-50	28821	immunoglobuli	IGLV150|V1-18	22	BCRSignalingPathway
IGLV1-51	28820	immunoglobuli	IGLV151|V1-19	22	BCRSignalingPathway
IGLV10-5	28772	immunoglobuli	IGLV1054|V1-20	22	BCRSignalingPathway
IGLV11-5	28770	immunoglobuli	IGLV1155|V4-6	22	BCRSignalingPathway
IGLV2-11	28816	immunoglobuli	IGLV211|V1-3	22	BCRSignalingPathway
IGLV2-14	28815	immunoglobuli	IGLV214|V1-4	22	BCRSignalingPathway
IGLV2-18	28814	immunoglobuli	IGLV218|V1-5	22	BCRSignalingPathway
IGLV2-23	28813	immunoglobuli	IGLV223|V1-7	22	BCRSignalingPathway
IGLV2-33	28811	immunoglobuli	IGLV233|V1-9	22	BCRSignalingPathway
IGLV2-8	28817	immunoglobuli	IGLV28|V1-2	22	BCRSignalingPathway
IGLV3-1	28809	immunoglobuli	IGLV31|V2-1	22	BCRSignalingPathway
IGLV3-10	28803	immunoglobuli	IGLV310|V2-7	22	BCRSignalingPathway
IGLV3-12	28802	immunoglobuli	IGLV312|V2-8	22	BCRSignalingPathway
IGLV3-16	28799	immunoglobuli	IGLV316|V2-11	22	BCRSignalingPathway
IGLV3-19	28797	immunoglobuli	IGLV319|V2-13|VL3I	22	BCRSignalingPathway
IGLV3-21	28796	immunoglobuli	IGLV321|V2-14	22	BCRSignalingPathway
IGLV3-22	28795	immunoglobuli	IGLV322|V2-15	22	BCRSignalingPathway
IGLV3-25	28793	immunoglobuli	IGLV325|V2-17	22	BCRSignalingPathway
IGLV3-27	28791	immunoglobuli	IGLV327|V2-19	22	BCRSignalingPathway
IGLV3-32	28787	immunoglobuli	IGLV332|V2-23P	22	BCRSignalingPathway
IGLV3-9	28804	immunoglobuli	IGLV39|V2-6	22	BCRSignalingPathway
IGLV4-3	28786	immunoglobuli	IGLV43|V5-1	22	BCRSignalingPathway
IGLV4-60	28785	immunoglobuli	IGLV460|V5-4	22	BCRSignalingPathway
IGLV4-69	28784	immunoglobuli	IGLV469|V5-6	22	BCRSignalingPathway
IGLV5-37	28783	immunoglobuli	IGLV537|V4-1	22	BCRSignalingPathway
IGLV5-39	28782	immunoglobuli	IGLV539	22	BCRSignalingPathway
IGLV5-45	28781	immunoglobuli	IGLV545|V4-2	22	BCRSignalingPathway
IGLV5-48	28780	immunoglobuli	IGLV548|V4-3	22	BCRSignalingPathway
IGLV5-52	28779	immunoglobuli	IGLV552|V4-4	22	BCRSignalingPathway
IGLV6-57	28778	immunoglobuli	IGLV657|V1-22	22	BCRSignalingPathway
IGLV7-43	28776	immunoglobuli	IGLV743|V3-2	22	BCRSignalingPathway
IGLV7-46	28775	immunoglobuli	IGLV746|V3-3	22	BCRSignalingPathway
IGLV8-61	28774	immunoglobuli	IGLV861|V3-4	22	BCRSignalingPathway
IGLV9-49	28773	immunoglobuli	IGLV949|V5-2	22	BCRSignalingPathway
C3	718	complement C3	AHUS5|ARMD9|ASP|C3	19	Chemokines
C5	727	complement C5	C5D|C5a|C5b|CPAMD4	9	Chemokines
CAMP	820	cathelicidin a	CAP-18|CAP18|CRAMI	3	Chemokines
CCL1	6346	C-C motif chem	I-309|P500|SCYA1|S	17	Chemokines
CCL11	6356	C-C motif chem	SCYA11	17	Chemokines
CCL13	6357	C-C motif chem	CKb10|MCP-4|NCC-1	17	Chemokines
CCL14	6358	C-C motif chem	CC-1|CC-3|CKB1|HCC	17	Chemokines
CCL15-CC	3E+05	CCL15-CCL14 re	CCL15|HCC-2|LKN-1	17	Chemokines
CCL15	6359	C-C motif chem	HCC-2|HMRP-2B|LKN-	17	Chemokines
CCL16	6360	C-C motif chem	CKb12|HCC-4|ILINCI	17	Chemokines
CCL17	6361	C-C motif chem	A-152E5.3|ABCD-2|S	16	Chemokines
CCL18	6362	C-C motif chem	AMAC-1|AMAC1|CKb7	17	Chemokines
CCL19	6363	C-C motif chem	CKb11|ELC|MIP-3b|M	9	Chemokines
CCL2	6347	C-C motif chem	GDCF-2|HC11|HSMCR3	17	Chemokines
CCL20	6364	C-C motif chem	CKb4|Exodus|LARC|M	2	Chemokines
CCL21	6366	C-C motif chem	6Ckine|CKb9|ECL|SC	9	Chemokines

CCL22	6367	C-C motif cher	A-152E5.1|ABCD-1|I	16	Chemokines
CCL23	6368	C-C motif cher	CK-BETA-8|CKb8|Ckl	17	Chemokines
CCL24	6369	C-C motif cher	Ckb-6|MPIF-2|MPIF2	7	Chemokines
CCL25	6370	C-C motif cher	Ckb15|SCYA25|TECK	19	Chemokines
CCL26	10344	C-C motif cher	IMAC|MIP-4a|MIP-4a	7	Chemokines
CCL27	10850	C-C motif cher	ALP|CTACK|CTAK|ESI	9	Chemokines
CCL28	56477	C-C motif cher	CCK1|MEC|SCYA28	5	Chemokines
CCL3	6348	C-C motif cher	G0S19-1|LD78ALPHA	17	Chemokines
CCL3L1	6349	C-C motif cher	464.2|D17S1718|G0S	17	Chemokines
CCL3P1	4E+05	C-C motif cher	CCL3L2|G0S19-3|LD7	17	Chemokines
CCL3L3	4E+05	C-C motif cher	464.2|D17S1718|G0S	17	Chemokines
CCL4	6351	C-C motif cher	ACT2|AT744.1|G-26	17	Chemokines
CCL4L2	9560	C-C motif cher	AT744.2|CCL4L|SCYA	17	Chemokines
CCL4L1	4E+05	C-C motif cher	AT744.2|CCL4L|LAG-	17	Chemokines
CCL5	6352	C-C motif cher	D17S136E|RANTES|SC	17	Chemokines
CCL7	6354	C-C motif cher	FIC|MARC|MCP-3|MCI	17	Chemokines
CCL8	6355	C-C motif cher	HC14|MCP-2|MCP2|SC	17	Chemokines
CKLF	51192	chemokine lik	C32|CKLF1|CKLF2|CI	16	Chemokines
CMA1	1215	chymase 1	CYH|MCT1|chymase	14	Chemokines
CTSG	1511	cathepsin G	CATG|CG	14	Chemokines
CX3CL1	6376	C-X3-C motif (	ABCD-3|C3Xkine|CXC	16	Chemokines
CXCL1	2919	C-X-C motif cl	FSP|GRO1|GROa|MGSA	4	Chemokines
CXCL10	3627	C-X-C motif cl	C7|IFI10|INP10|IP-	4	Chemokines
CXCL11	6373	C-X-C motif cl	H174|I-TAC|IP-9|II	4	Chemokines
CXCL12	6387	C-X-C motif cl	IRH|PBSF|SCYB12|SI	10	Chemokines
CXCL13	10563	C-X-C motif cl	ANGIE|ANGIE2|BCA-1	4	Chemokines
CXCL14	9547	C-X-C motif cl	BMAC|BRAK|KEC|KS1	5	Chemokines
CXCL16	58191	C-X-C motif cl	CXCLG16|SR-PSOX|SI	17	Chemokines
CXCL17	3E+05	C-X-C motif cl	DMC|Dcip1|UNQ473|V	19	Chemokines
CXCL2	2920	C-X-C motif cl	CINC-2a|GRO2|GROb	4	Chemokines
CXCL3	2921	C-X-C motif cl	CINC-2b|GRO3|GROg	4	Chemokines
CXCL5	6374	C-X-C motif cl	ENA-78|SCYB5	4	Chemokines
CXCL6	6372	C-X-C motif cl	CKA-3|GCP-2|GCP2|S	4	Chemokines
CXCL9	4283	C-X-C motif cl	CMK|Humig|MIG|SCYI	4	Chemokines
CCN1	3491	cellular comm	CYR61|GIG1|IGFBP1(	1	Chemokines
DEFA1	1667	defensin alpha	DEF1|DEFA2|HNP-1|I	8	Chemokines
DEFA3	1668	defensin alpha	DEF3|HNP-3|HNP3|HI	8	Chemokines
DEFA5	1670	defensin alpha	DEF5|HD-5	8	Chemokines
DEFB1	1672	defensin beta	BD1|DEFB-1|DEFB10	8	Chemokines
DEFB103B	55894	defensin beta	BD-3|DEFB-3|DEFB1(	8	Chemokines
DEFB104A	1E+05	defensin beta	BD-4|DEFB-4|DEFB1(	8	Chemokines
DEFB4A	1673	defensin beta	BD-2|DEFB-2|DEFB1(	8	Chemokines
EDN1	1906	endothelin 1	ARCND3|ET1|HDLCQ7	6	Chemokines
EDN2	1907	endothelin 2	ET-2|ET2|PPET2	1	Chemokines
EDN3	1908	endothelin 3	ET-3|ET3|HSCR4|PPI	20	Chemokines
FGF10	2255	fibroblast gr(	-	5	Chemokines
FGF2	2247	fibroblast gr(	BFGF|FGF-2|FGFB|HI	4	Chemokines
HTN3	3347	histatin 3	HIS2|HTN2|HTN5|PB	4	Chemokines
CXCL8	3576	C-X-C motif cl	GCP-1|GCP1|IL8|LEC	4	Chemokines
LECT2	3950	leukocyte cel	chm-II|chm2	5	Chemokines
PF4	5196	platelet facto	CXCL4|PF-4|SCYB4	4	Chemokines
PF4V1	5197	platelet facto	CXCL4L1|CXCL4V1|PI	4	Chemokines
PLAU	5328	plasminogen ac	ATF|BDPLT5|QPD|UPA	10	Chemokines
PPBP	5473	pro-platelet	B-TG1|Beta-TG|CTAI	4	Chemokines

PPBPP1	7E+05	pro-platelet l	PPBPL1|TGB2	4	Chemokines
PROK2	60675	prokineticin 2	BV8|HH4|KAL4|MIT1	3	Chemokines
RNASE2	6036	ribonuclease A	EDN|RAF3|RNS2	14	Chemokines
SAA1	6288	serum amyloid	PIG4|SAA|SAA2|TP53	11	Chemokines
SAA2	6289	serum amyloid	SAA|SAA1	11	Chemokines
SBDS	51119	SBDS ribosome	CGI-97|SDS|SWDS	7	Chemokines
SEMA3A	10371	semaphorin 3A	COLL1|HH16|Hsema-I	7	Chemokines
SEMA3B	7869	semaphorin 3B	LUCA-1|SEMA5|SEMA	3	Chemokines
SEMA3C	10512	semaphorin 3C	SEMAE|SemE	7	Chemokines
SEMA3D	2E+05	semaphorin 3D	Sema-Z2|coll-2	7	Chemokines
SEMA3E	9723	semaphorin 3E	M-SEMAH|M-SemaK|SI	7	Chemokines
SEMA3F	6405	semaphorin 3F	SEMA-IV|SEMA4|SEM	3	Chemokines
SEMA3G	56920	semaphorin 3G	sem2	3	Chemokines
SEMA4A	64218	semaphorin 4A	CORD10|RP35|SEMAB	1	Chemokines
SEMA4B	10509	semaphorin 4B	SEMAC|SemC	15	Chemokines
SEMA4C	54910	semaphorin 4C	M-SEMA-F|SEMACL1|S	2	Chemokines
SEMA4D	10507	semaphorin 4D	A8|BB18|C9orf164|C	9	Chemokines
SEMA4F	10505	ssemaphorin 4I	M-SEMA|PRO2353|S4I	2	Chemokines
SEMA4G	57715	semaphorin 4G	-	10	Chemokines
SEMA5A	9037	semaphorin 5A	SEMAF|semF	5	Chemokines
SEMA5B	54437	semaphorin 5B	SEMAG|SemG	3	Chemokines
SEMA6A	57556	semaphorin 6A	HT018|SEMA|SEMA6A1	5	Chemokines
SEMA6B	10501	semaphorin 6B	EPM11|SEM-SEMA-Y|S	19	Chemokines
SEMA6C	10500	semaphorin 6C	SEMAY|m-SemaY|m-Se	1	Chemokines
SEMA6D	80031	semaphorin 6D	-	15	Chemokines
SEMA7A	8482	semaphorin 7A	CD108|CDw108|H-SEM	15	Chemokines
SLIT1	6585	slit guidance	MEGF4|SLIL1|SLIT-1	10	Chemokines
SLIT2	9353	slit guidance	SLIL3|Slit-2	4	Chemokines
TNC	3371	tenascin C	150-225|DFNA56|GMI	9	Chemokines
TYMP	1890	thymidine pho	ECGF|ECGF1|MEDPS1	22	Chemokines
XCL1	6375	X-C motif chem	ATAC|LPTN|LTN|SCM-	1	Chemokines
XCL2	6846	X-C motif chem	SCM-1b|SCM1B|SCYC2	1	Chemokines
C5AR1	728	complement C5a	C5A|C5AR|C5R1|CD88	19	Chemokine_Receptors
ACKR2	1238	atypical chem	CCBP2|CCR10|CCR9|C	3	Chemokine_Receptors
CCR1	1230	C-C motif chem	CD191|CKR-1|CKR1|C	3	Chemokine_Receptors
CCR10	2826	C-C motif chem	GPR2	17	Chemokine_Receptors
CCR3	1232	C-C motif chem	C C CKR3|CC-CKR-3	3	Chemokine_Receptors
CCR4	1233	C-C motif chem	CC-CKR-4|CD194|CKI	3	Chemokine_Receptors
CCR5	1234	C-C motif chem	CC-CKR-5|CCCKR5|CC	3	Chemokine_Receptors
CCR6	1235	C-C motif chem	BN-1|C-C CKR-6|CC-	6	Chemokine_Receptors
CCR7	1236	C-C motif chem	BLR2|CC-CKR-7|CCR-	17	Chemokine_Receptors
CCR8	1237	C-C motif chem	CC-CKR-8|CCR-8|CDw	3	Chemokine_Receptors
CCR9	10803	C-C motif chem	CC-CKR-9|CDw199|GI	3	Chemokine_Receptors
ACKR4	51554	atypical chem	CC-CKR-11|CCBP2|CC	3	Chemokine_Receptors
CCRL2	9034	C-C motif chem	ACKR5|CKRX|CRAM|CI	3	Chemokine_Receptors
CMKLR1	1240	chemerin chem	CHEMERINR|ChemR23	12	Chemokine_Receptors
CX3CR1	1524	C-X3-C motif c	CCRL1|CMKBRL1|CMKI	3	Chemokine_Receptors
CXCR3	2833	C-X-C motif cl	CD182|CD183|CKR-L2	X	Chemokine_Receptors
CXCR4	7852	C-X-C motif cl	CD184|D2S201E|FB22	2	Chemokine_Receptors
CXCR5	643	C-X-C motif cl	BLR1|CD185|MDR15	11	Chemokine_Receptors
CXCR6	10663	C-X-C motif cl	BONZO|CD186|STRL33	3	Chemokine_Receptors
ACKR3	57007	atypical chem	CMKOR1|CXC-R7|CXCI	2	Chemokine_Receptors
CYSLTR1	10800	cysteinyl leul	CYSLT1|CYSLT1R|CYS	X	Chemokine_Receptors
CYSLTR2	57105	cysteinyl leul	CYSLT2|CYSLT2R|GPC	13	Chemokine_Receptors

ACKR1	2532	atypical chem	CCBP1|CD234|DARC|I		1	Chemokine_Receptors
EDNRA	1909	endothelin re	ET-A|ETA|ETA-R|ET/		4	Chemokine_Receptors
EDNRB	1910	endothelin re	ABCDS|ET-B|ET-BR|I		13	Chemokine_Receptors
FPR1	2357	formyl peptid	FMLP|FPR		19	Chemokine_Receptors
FPR2	2358	formyl peptid	ALXR|FMLP-R-II|FMI		19	Chemokine_Receptors
FPR2	2358	formyl peptid	ALXR|FMLP-R-II|FMI		19	Chemokine_Receptors
GPR17	2840	G protein-cou	-		2	Chemokine_Receptors
GPR32	2854	G protein-cou	RVDR1		19	Chemokine_Receptors
GPR33	2856	G protein-cou	-		14	Chemokine_Receptors
PTGDR2	11251	prostaglandin	CD294|CRTH2|DL1R|I		11	Chemokine_Receptors
C5AR2	27202	complement co	C5L2|GPF77|GPR77		19	Chemokine_Receptors
CXCR1	3577	C-X-C motif cl	C-C|C-C-CKR-1|CD1		2	Chemokine_Receptors
CXCR2	3579	C-X-C motif cl	CD182|CDw128b|CMK/		2	Chemokine_Receptors
LTB4R	1241	leukotriene B	BLT1|BLTR|CMKRL1|(		14	Chemokine_Receptors
LTB4R2	56413	leukotriene B	BLT2|BLTR2|JULF2|I		14	Chemokine_Receptors
PLAUR	5329	plasminogen a	CD87|U-PAR|UPAR|UI		19	Chemokine_Receptors
PLXNA1	5361	plexin A1	NOV|NOVP|PLEXIN-A		3	Chemokine_Receptors
PLXNA2	5362	plexin A2	OCT|PLXN2		1	Chemokine_Receptors
PLXNA3	55558	plexin A3	6.3|HSSEXGENE|PLXI	X		Chemokine_Receptors
PLXNA4	91584	plexin A4	FAYV2820|PLEXA4|PI		7	Chemokine_Receptors
PLXNB1	5364	plexin B1	PLEXIN-B1|PLXN5|SI		3	Chemokine_Receptors
PLXNB2	23654	plexin B2	MM1|Nbla00445|PLE		22	Chemokine_Receptors
PLXNB3	5365	plexin B3	PLEXB3|PLEXR|PLXN(	X		Chemokine_Receptors
PLXNC1	10154	plexin C1	CD232|PLXN-C1|VESI		12	Chemokine_Receptors
PLXND1	23129	plexin D1	PLEXD1		3	Chemokine_Receptors
PTAFR	5724	platelet acti	PAFR		1	Chemokine_Receptors
ROBO1	6091	roundabout gu	DUTT1|SAX3		3	Chemokine_Receptors
ROBO2	6092	roundabout gu	SAX3		3	Chemokine_Receptors
ROBO3	64221	roundabout gu	HGPPS|HGPPS1|HGPS		11	Chemokine_Receptors
RXFP3	51289	relaxin famil	GPCR135|RLN3R1|RXI		5	Chemokine_Receptors
XCR1	2829	X-C motif che	CCXCR1|GPR5		3	Chemokine_Receptors
ADIPOQ	9370	adiponectin, (	ACDC|ACRP30|ADIPQ		3	Cytokines
ADM	133	adrenomedulli	AM|PAMP		11	Cytokines
ADM2	79924	adrenomedulli	AM2|dJ579N16.4		22	Cytokines
AGRP	181	agouti relate	AGRT|ART|ASIP2		16	Cytokines
AGT	183	angiotensinog	ANHU|SERPINA8|hFL		1	Cytokines
AMBN	258	ameloblastin	AI1F		4	Cytokines
AMELX	265	amelogenin X-	AI1E|AIH1|ALGN|AM(	X		Cytokines
AMH	268	anti-Mulleria	MIF|MIS		19	Cytokines
ANGPTL5	3E+05	angiopoietin	-		11	Cytokines
ANGPTL7	10218	angiopoietin	AngX|CDT6|dJ647M1(		1	Cytokines
APLN	8862	apelin	APEL|XNPEP2	X		Cytokines
AREG	374	amphiregulin	AR|AREGB|CRDGF|SD(		4	Cytokines
MANF	7873	mesencephalic	ARMET|ARP		3	Cytokines
CDNF	4E+05	cerebral dopa	ARMETL1		10	Cytokines
ARTN	9048	artemin	ART|ENOVIN|EVN|NBI		1	Cytokines
AVP	551	arginine vaso	ADH|ARVP|AVP-NPII		20	Cytokines
AZU1	566	azurocidin 1	AZAMP|AZU|CAP37|HI		19	Cytokines
BDNF	627	brain derived	ANON2|BULN2		11	Cytokines
BMP1	649	bone morphoge	OI13|PCOLC|PCP|PCI		8	Cytokines
BMP10	27302	bone morphoge	-		2	Cytokines
BMP15	9210	bone morphoge	GDF9B|ODG2|POF4	X		Cytokines
BMP2	650	bone morphoge	BDA2|BMP2A|SSFSC		20	Cytokines
BMP3	651	bone morphoge	BMP-3A		4	Cytokines

BMP4	652	bone morphoge	BMP2B|BMP2B1|MCOP	14	Cytokines
BMP5	653	bone morphoge	-	6	Cytokines
BMP6	654	bone morphoge	VGR|VGR1	6	Cytokines
BMP7	655	bone morphoge	OP-1	20	Cytokines
BMP8A	4E+05	bone morphoge	OP-2	1	Cytokines
BMP8B	656	bone morphoge	BMP8|OP2	1	Cytokines
BTC	685	betacellulin	-	4	Cytokines
MYDGF	56005	myeloid deriv	C19orf10|EUROIMAG	19	Cytokines
C3	718	complement C3	AHUS5|ARMD9|ASP|C	19	Cytokines
C5	727	complement C5	C5D|C5a|C5b|CPAMD	9	Cytokines
CALCA	796	calcitonin re	CALC1|CGRP|CGRP-I	11	Cytokines
CALCB	797	calcitonin re	CALC2|CGRP-II|CGR	11	Cytokines
CAMP	820	cathelicidin	CAP-18|CAP18|CRAM	3	Cytokines
CAT	847	catalase	-	11	Cytokines
CCK	885	cholecystokin	-	3	Cytokines
CCL1	6346	C-C motif che	I-309|P500|SCYA1|	17	Cytokines
CCL11	6356	C-C motif che	SCYA11	17	Cytokines
CCL13	6357	C-C motif che	CKb10|MCP-4|NCC-1	17	Cytokines
CCL14	6358	C-C motif che	CC-1|CC-3|CKB1|HC	17	Cytokines
CCL15-CC	3E+05	CCL15-CCL14 r	CCL15|HCC-2|LKN-1	17	Cytokines
CCL15	6359	C-C motif che	HCC-2|HMRP-2B|LKN	17	Cytokines
CCL16	6360	C-C motif che	CKb12|HCC-4|ILINC	17	Cytokines
CCL17	6361	C-C motif che	A-152E5.3|ABCD-2|	16	Cytokines
CCL18	6362	C-C motif che	AMAC-1|AMAC1|CKb7	17	Cytokines
CCL19	6363	C-C motif che	CKb11|ELC|MIP-3b|	9	Cytokines
CCL2	6347	C-C motif che	GDCF-2|HC11|HSMCR	17	Cytokines
CCL20	6364	C-C motif che	CKb4|Exodus|LARC|	2	Cytokines
CCL21	6366	C-C motif che	6Ckine|CKb9|ECL|S	9	Cytokines
CCL22	6367	C-C motif che	A-152E5.1|ABCD-1|	16	Cytokines
CCL23	6368	C-C motif che	CK-BETA-8|CKb8|Ck	17	Cytokines
CCL24	6369	C-C motif che	Ckb-6|MPIF-2|MPIF	7	Cytokines
CCL25	6370	C-C motif che	Ckb15|SCYA25|TECK	19	Cytokines
CCL26	10344	C-C motif che	IMAC|MIP-4a|MIP-4	7	Cytokines
CCL27	10850	C-C motif che	ALP|CTACK|CTAK|ES	9	Cytokines
CCL28	56477	C-C motif che	CCK1|MEC|SCYA28	5	Cytokines
CCL3	6348	C-C motif che	G0S19-1|LD78ALPHA	17	Cytokines
CCL3L1	6349	C-C motif che	464.2|D17S1718|G0	17	Cytokines
CCL3P1	4E+05	C-C motif che	CCL3L2|G0S19-3|LD	17	Cytokines
CCL3L3	4E+05	C-C motif che	464.2|D17S1718|G0	17	Cytokines
CCL4	6351	C-C motif che	ACT2|AT744.1|G-26	17	Cytokines
CCL4L2	9560	C-C motif che	AT744.2|CCL4L|SCY	17	Cytokines
CCL4L1	4E+05	C-C motif che	AT744.2|CCL4L|LAG	17	Cytokines
CCL5	6352	C-C motif che	D17S136E|RANTES|S	17	Cytokines
CCL7	6354	C-C motif che	FIC|MARC|MCP-3|MC	17	Cytokines
CCL8	6355	C-C motif che	HC14|MCP-2|MCP2|S	17	Cytokines
CD320	51293	CD320 molecul	8D6|8D6A|TCBLR|TC	19	Cytokines
CD40LG	959	CD40 ligand	CD154|CD40L|HIGM1	X	Cytokines
CD70	970	CD70 molecule	CD27-L|CD27L|CD27	19	Cytokines
ADA2	51816	adenosine dea	ADGF|CECR1|IDGFL|	22	Cytokines
CER1	9350	cerberus 1, D	DAND4	9	Cytokines
CGA	1081	glycoprotein	CG-ALPHA|FSHA|GPA	6	Cytokines
CGB3	1082	chorionic gon	CGB|CGB5|CGB7|CGB	19	Cytokines
CGB1	1E+05	chorionic gon	-	19	Cytokines
CGB2	1E+05	chorionic gon	-	19	Cytokines

CGB5	93659	chorionic gon	CGB|HCG|hCGB	19	Cytokines
CGB7	94027	chorionic gon	CG-beta-a|CGB6	19	Cytokines
CGB8	94115	chorionic gon	-	19	Cytokines
CHGA	1113	chromogranin	CGA	14	Cytokines
CHGB	1114	chromogranin	SCG1	20	Cytokines
CKLF	51192	chemokine lik	C32|CKLF1|CKLF2|C	16	Cytokines
CLCF1	23529	cardiotrophin	BSF-3|BSF3|CISS2|	11	Cytokines
CLEC11A	6320	C-type lectin	CLECSF3|LSLCL|P47	19	Cytokines
CMA1	1215	chymase 1	CYH|MCT1|chymase	14	Cytokines
CMTM1	1E+05	CKLF like MAR	CKLFH|CKLFH1|CKLF	16	Cytokines
CMTM2	1E+05	CKLF like MAR	CKLFSF2	16	Cytokines
CMTM3	1E+05	CKLF like MAR	BNAS2|CKLFSF3	16	Cytokines
CMTM4	1E+05	CKLF like MAR	CKLFSF4	16	Cytokines
CMTM5	1E+05	CKLF like MAR	CKLFSF5	14	Cytokines
CMTM6	54918	CKLF like MAR	CKLFSF6|PRO2219	3	Cytokines
CMTM7	1E+05	CKLF like MAR	CKLFSF7	3	Cytokines
CMTM8	2E+05	CKLF like MAR	CKLFSF8|CKLFSF8-V	3	Cytokines
CNTF	1270	ciliary neuro	HCNTF	11	Cytokines
CORT	1325	cortistatin	CST-14|CST-17|CST-	1	Cytokines
CRH	1392	corticotropin	CRF|CRH1	8	Cytokines
CSF1	1435	colony stimul	CSF-1|MCSF	1	Cytokines
CSF2	1437	colony stimul	CSF|GMCSF	5	Cytokines
CSF3	1440	colony stimul	C17orf33|CSF3OS|G	17	Cytokines
CSH1	1442	chorionic som	CS-1|CSA|CSMT|GHB	17	Cytokines
CSH2	1443	chorionic som	CS-2|CSB|GHB1|PL|	17	Cytokines
CSHL1	1444	chorionic som	CS-5|CSHP1|CSL|GH	17	Cytokines
CSPG5	10675	chondroitin s	NGC	3	Cytokines
CTF1	1489	cardiotrophin	CT-1|CT1	16	Cytokines
CCN2	1490	cellular comm	CTGF|HCS24|IGFBP8	6	Cytokines
CTSG	1511	cathepsin G	CATG|CG	14	Cytokines
CX3CL1	6376	C-X3-C motif	ABCD-3|C3Xkine|CX	16	Cytokines
CXCL1	2919	C-X-C motif c	FSP|GRO1|GROa|MGS	4	Cytokines
CXCL10	3627	C-X-C motif c	C7|IFI10|INP10|IP-	4	Cytokines
CXCL11	6373	C-X-C motif c	H174|I-TAC|IP-9|I	4	Cytokines
CXCL12	6387	C-X-C motif c	IRH|PBSF|SCYB12|S	10	Cytokines
CXCL13	10563	C-X-C motif c	ANGIE|ANGIE2|BCA-	4	Cytokines
CXCL14	9547	C-X-C motif c	BMAC|BRAK|KEC|KS1	5	Cytokines
CXCL16	58191	C-X-C motif c	CXCLG16|SR-PSOX|S	17	Cytokines
CXCL17	3E+05	C-X-C motif c	DMC|Dcip1|UNQ473|	19	Cytokines
CXCL2	2920	C-X-C motif c	CINC-2a|GRO2|GROb	4	Cytokines
CXCL3	2921	C-X-C motif c	CINC-2b|GRO3|GROg	4	Cytokines
CXCL5	6374	C-X-C motif c	ENA-78|SCYB5	4	Cytokines
CXCL6	6372	C-X-C motif c	CKA-3|GCP-2|GCP2|	4	Cytokines
CXCL9	4283	C-X-C motif c	CMK|Humig|MIG|SCY	4	Cytokines
CCN1	3491	cellular comm	CYR61|GIG1|IGFBP1	1	Cytokines
DEFA1	1667	defensin alph	DEF1|DEFA2|HNP-1|	8	Cytokines
DEFA3	1668	defensin alph	DEF3|HNP-3|HNP3|H	8	Cytokines
DEFA5	1670	defensin alph	DEF5|HD-5	8	Cytokines
DEFB1	1672	defensin beta	BD1|DEFB-1|DEFB10	8	Cytokines
DEFB103B	55894	defensin beta	BD-3|DEFB-3|DEFB1	8	Cytokines
DEFB104A	1E+05	defensin beta	BD-4|DEFB-4|DEFB1	8	Cytokines
DEFB4A	1673	defensin beta	BD-2|DEFB-2|DEFB1	8	Cytokines
DKK1	22943	dickkopf WNT	DKK-1|SK	10	Cytokines
EBI3	10148	Epstein-Barr	IL-27B|IL27B|IL35	19	Cytokines

EDN1	1906	endothelin 1	ARCND3|ET1|HDLCQ7		6	Cytokines
EDN2	1907	endothelin 2	ET-2|ET2|PPET2		1	Cytokines
EDN3	1908	endothelin 3	ET-3|ET3|HSCR4|PPI		20	Cytokines
EGF	1950	epidermal grow	HOMG4|URG		4	Cytokines
EPGN	3E+05	epithelial mi	ALGV3072|EPG|PRO99		4	Cytokines
EPO	2056	erythropoietin	DBAL|ECYT5|EP|MVCI		7	Cytokines
EREG	2069	epiregulin	EPR|ER|Ep		4	Cytokines
ESM1	11082	endothelial ce	endocan		5	Cytokines
FAM3B	54097	FAM3 metaboli	2-21|C21orf11|C21c		21	Cytokines
FAM3C	10447	FAM3 metaboli	GS3786|ILEI		7	Cytokines
FAM3D	1E+05	FAM3 metaboli	EF7|OIT1		3	Cytokines
FASLG	356	Fas ligand	ALPS1B|APT1LG1|APT		1	Cytokines
FGF1	2246	fibroblast gro	AFGF|ECGF|ECGF-be		5	Cytokines
FGF10	2255	fibroblast gro	-		5	Cytokines
FGF11	2256	fibroblast gro	FGF-11|FHF-3|FHF3		17	Cytokines
FGF12	2257	fibroblast gro	EIEE47|FGF12B|FHF		3	Cytokines
FGF13	2258	fibroblast gro	FGF-13|FGF2|FHF-2	X		Cytokines
FGF14	2259	fibroblast gro	FGF-14|FHF-4|FHF4		13	Cytokines
FGF16	8823	fibroblast gro	FGF-16|MF4	X		Cytokines
FGF17	8822	fibroblast gro	FGF-13|FGF-17|HH20		8	Cytokines
FGF18	8817	fibroblast gro	FGF-18|ZFGF5		5	Cytokines
FGF19	9965	fibroblast gro	-		11	Cytokines
FGF2	2247	fibroblast gro	BFGF|FGF-2|FGFB|HI		4	Cytokines
FGF20	26281	fibroblast gro	FGF-20|RHDA2		8	Cytokines
FGF21	26291	fibroblast gro	-		19	Cytokines
FGF22	27006	fibroblast gro	-		19	Cytokines
FGF23	8074	fibroblast gro	ADHR|FGFN|HFTC2|HI		12	Cytokines
FGF3	2248	fibroblast gro	HBGF-3|INT2		11	Cytokines
FGF4	2249	fibroblast gro	FGF-4|HBGF-4|HST|I		11	Cytokines
FGF5	2250	fibroblast gro	HBGF-5|Smag-82|TCN		4	Cytokines
FGF6	2251	fibroblast gro	HBGF-6|HST2		12	Cytokines
FGF7	2252	fibroblast gro	HBGF-7|KGF		15	Cytokines
FGF8	2253	fibroblast gro	AIGF|FGF-8|HBGF-8		10	Cytokines
FGF9	2254	fibroblast gro	FGF-9|GAF|HBFG-9|I		13	Cytokines
VEGFD	2277	vascular endo	FIGF|VEGF-D	X		Cytokines
FIGNL2	4E+05	fidgetin like	-		12	Cytokines
FLT3LG	2323	fms related re	FL|FLG3L|FLT3L		19	Cytokines
FSHB	2488	follicle stimu	HH24		11	Cytokines
GAL	51083	galanin and GM	ETL8|GAL-GMAP|GALN		11	Cytokines
GALP	85569	galanin like p	-		19	Cytokines
GAST	2520	gastrin	GAS		17	Cytokines
GCG	2641	glucagon	GLP-1|GLP1|GLP2|GI		2	Cytokines
GDF1	2657	growth differe	CERS1|CHTD6|DORV|I		19	Cytokines
GDF10	2662	growth differe	BIP|BMP-3b|BMP3B		10	Cytokines
GDF11	10220	growth differe	BMP-11|BMP11		12	Cytokines
GDF15	9518	growth differe	GDF-15|MIC-1|MIC1		19	Cytokines
GDF2	2658	growth differe	BMP-9|BMP9|HHT5		10	Cytokines
GDF3	9573	growth differe	KFS3|MCOP7|MCOPCB6		12	Cytokines
GDF5	8200	growth differe	BDA1C|BMP-14|BMP14		20	Cytokines
GDF6	4E+05	growth differe	BMP-13|BMP13|CDMP2		8	Cytokines
GDF7	2E+05	growth differe	BMP12		2	Cytokines
GDF9	2661	growth differe	POF14		5	Cytokines
GDNF	2668	glial cell der	ATF|ATF1|ATF2|HFB1		5	Cytokines
GH1	2688	growth hormone	GH|GH-N|GHB5|GHN|I		17	Cytokines

GH2	2689	growth hormon(	GH-V|GHB2|GHL|GHV	17	Cytokines
GHRH	2691	growth hormon(	GHRF|GRF|INN	20	Cytokines
GHRL	51738	ghrelin and ol	MTLRP	3	Cytokines
GIP	2695	gastric inhib:	-	17	Cytokines
GKN1	56287	gastrokine 1	AMP18|BRICD1|CA11	2	Cytokines
GMFB	2764	glia maturati(	GMF	14	Cytokines
GMFG	9535	glia maturati(	GMF-GAMMA	19	Cytokines
GNRH1	2796	gonadotropin :	GNRH|GRH|LHRH|LNRI	8	Cytokines
GNRH2	2797	gonadotropin :	GnRH-II|LH-RHII	20	Cytokines
GPHA2	2E+05	glycoprotein l	A2|GPA2|ZSIG51	11	Cytokines
GPHB5	1E+05	glycoprotein l	B5|GPB5|ZLUT1	14	Cytokines
GPI	2821	glucose-6-pho:	AMF|GNPI|NLK|PGI|I	19	Cytokines
GREM1	26585	gremlin 1, DAI	C15DUPq|CKTSF1B1|(	15	Cytokines
GREM2	64388	gremlin 2, DAI	CKTSF1B2|DAND3|PRI	1	Cytokines
GRN	2896	granulin prec(	CLN11|GEP|GP88|PCI	17	Cytokines
GRP	2922	gastrin relea:	BN|GRP-10|preproGI	18	Cytokines
GUCA2A	2980	guanylate cyc:	GCAP-I|GUCA2|STAR/	1	Cytokines
HAMP	57817	hepcidin anti	HEPC|HFE2B|LEAP1|I	19	Cytokines
HBEGF	1839	heparin bindi	DTR|DTS|DTSF|HEGFI	5	Cytokines
HDGF	3068	heparin bindi	HMG1L2	1	Cytokines
HDGFL3	50810	HDGF like 3	CGI-142|HDGF-2|HD(	15	Cytokines
HGF	3082	hepatocyte gr(	DFNB39|F-TCF|HGFB	7	Cytokines
HTN3	3347	histatin 3	HIS2|HTN2|HTN5|PB	4	Cytokines
IAPP	3375	islet amyloid	DAP|IAP	12	Cytokines
IFNA1	3439	interferon al	IFL|IFN|IFN-ALPHA	9	Cytokines
IFNA10	3446	interferon al	IFN-alphaC	9	Cytokines
IFNA13	3447	interferon al	-	9	Cytokines
IFNA14	3448	interferon al	IFN-alphaH|LEIF2H	9	Cytokines
IFNA16	3449	interferon al	IFN-alpha-16|IFN-a	9	Cytokines
IFNA17	3451	interferon al	IFN-alphaI|IFNA|II	9	Cytokines
IFNA2	3440	interferon al	IFN-alpha-2|IFN-a	9	Cytokines
IFNA21	3452	interferon al	IFN-alphaI|LeIF F	9	Cytokines
IFNA4	3441	interferon al	IFN-alpha4a|INFA4	9	Cytokines
IFNA5	3442	interferon al	IFN-alpha-5|IFN-a	9	Cytokines
IFNA6	3443	interferon al	IFN-alphaK	9	Cytokines
IFNA7	3444	interferon al	IFN-alphaJ|IFNA-J	9	Cytokines
IFNA8	3445	interferon al	IFN-alphaB	9	Cytokines
IFNB1	3456	interferon be	IFB|IFF|IFN-beta|	9	Cytokines
IFNE	3E+05	interferon ep:	IFN-E|IFNE1|IFNT1	9	Cytokines
IFNG	3458	interferon gai	IFG|IFI	12	Cytokines
IFNK	56832	interferon ka	IFNT1|INFE1	9	Cytokines
IFNW1	3467	interferon om(	-	9	Cytokines
IGF1	3479	insulin like {	IGF|IGF-I|IGFI|MGI	12	Cytokines
IGF2	3481	insulin like {	C11orf43|GRDF|IGF-	11	Cytokines
IL10	3586	interleukin 1(	CSIF|GVHDS|IL-10|	1	Cytokines
IL11	3589	interleukin 1	AGIF|IL-11	19	Cytokines
IL12A	3592	interleukin 1:	CLMF|IL-12A|NFSK|I	3	Cytokines
IL12B	3593	interleukin 1:	CLMF|CLMF2|IL-12B	5	Cytokines
IL13	3596	interleukin 1:	IL-13|P600	5	Cytokines
IL15	3600	interleukin 1!	IL-15	4	Cytokines
IL16	3603	interleukin 1(	LCF|NIL16|PRIL16|	15	Cytokines
IL17A	3605	interleukin 1'	CTLA-8|CTLA8|IL-1'	6	Cytokines
IL17B	27190	interleukin 1'	IL-17B|IL-20|NIRF	5	Cytokines
IL17C	27189	interleukin 1'	CX2|IL-17C	16	Cytokines

IL17D	53342	interleukin 1'	IL-17D	13	Cytokines
IL17F	1E+05	interleukin 1'	CANDF6|IL-17F|ML-1	6	Cytokines
IL18	3606	interleukin 18	IGIF|IL-18|IL-1g|1	11	Cytokines
IL19	29949	interleukin 19	IL-10C|MDA1|NG.1|Z	1	Cytokines
IL1A	3552	interleukin 1	IL-1 alpha|IL-1A|1	2	Cytokines
IL1B	3553	interleukin 1	IL-1|IL1-BETA|IL1I	2	Cytokines
IL1F10	84639	interleukin 1	FIL1-theta|FKSG75	2	Cytokines
IL36RN	26525	interleukin 3(	FIL1|FIL1(DELTA)|I	2	Cytokines
IL36A	27179	interleukin 3(	FIL1|FIL1(EPSILON)	2	Cytokines
IL37	27178	interleukin 3'	FIL1|FIL1(ZETA)|F	2	Cytokines
IL36B	27177	interleukin 3(	FIL1|FIL1-(ETA)|F	2	Cytokines
IL36G	56300	interleukin 3(	IL-1F9|IL-1H1|IL-1	2	Cytokines
IL1RN	3557	interleukin 1	DIRA|ICIL-1RA|IL-1	2	Cytokines
IL2	3558	interleukin 2	IL-2|TCGF|lymphoki	4	Cytokines
IL20	50604	interleukin 2(	IL-20|IL10D|ZCYTO	1	Cytokines
IL21	59067	interleukin 2	CVID11|IL-21|Za11	4	Cytokines
IL22	50616	interleukin 2:	IL-21|IL-22|IL-D1	12	Cytokines
IL23A	51561	interleukin 2:	IL-23|IL-23A|IL23I	12	Cytokines
IL24	11009	interleukin 2	C49A|FISP|IL10B|MI	1	Cytokines
IL25	64806	interleukin 2!	IL17E	14	Cytokines
IL26	55801	interleukin 2(	AK155|IL-26	12	Cytokines
IL27	2E+05	interleukin 2'	IL-27|IL-27A|IL27	16	Cytokines
IFNL2	3E+05	interferon lar	IL-28A|IL28A	19	Cytokines
IFNL3	3E+05	interferon lar	IFN-lambda-3|IFN-1	19	Cytokines
IFNL1	3E+05	interferon lar	IL-29|IL29	19	Cytokines
IL3	3562	interleukin 3	IL-3|MCGF|MULTI-CS	5	Cytokines
IL31	4E+05	interleukin 3	IL-31	12	Cytokines
IL32	9235	interleukin 3:	IL-32alpha|IL-32be	16	Cytokines
IL33	90865	interleukin 3:	C9orf26|DVS27|IL1I	9	Cytokines
IL34	1E+05	interleukin 3	C16orf77|IL-34	16	Cytokines
IL4	3565	interleukin 4	BCGF-1|BCGF1|BSF-1	5	Cytokines
IL5	3567	interleukin 5	EDF|IL-5|TRF	5	Cytokines
IL6	3569	interleukin 6	BSF-2|BSF2|CDF|HGI	7	Cytokines
IL6ST	3572	interleukin 6	CD130|CDW130|GP13(	5	Cytokines
IL7	3574	interleukin 7	IL-7	8	Cytokines
CXCL8	3576	C-X-C motif cl	GCP-1|GCP1|IL8|LE(	4	Cytokines
IL9	3578	interleukin 9	HP40|IL-9|P40	5	Cytokines
INHA	3623	inhibin subun	-	2	Cytokines
INHBA	3624	inhibin subun	EDF|FRP	7	Cytokines
INHBB	3625	inhibin subun	-	2	Cytokines
INHBC	3626	inhibin subun	IHBC	12	Cytokines
INHBE	83729	inhibin subun	-	12	Cytokines
INS	3630	insulin	IDDM|IDDM1|IDDM2|1	11	Cytokines
INS-IGF2	7E+05	INS-IGF2 read	INSIGF	11	Cytokines
INSL3	3640	insulin like :	RLF|RLNL|ley-I-L	19	Cytokines
INSL4	3641	insulin like	EPIL|PLACENTIN	9	Cytokines
INSL5	10022	insulin like !	PRO182|UNQ156	1	Cytokines
INSL6	11172	insulin like (	RIF1	9	Cytokines
JAG1	182	jagged canoni(	AGS|AGS1|AHD|AWS|(	20	Cytokines
JAG2	3714	jagged canoni(	HJ2|SER2	14	Cytokines
FGF7P6	4E+05	fibroblast gr(	KGFLP1	9	Cytokines
FGF7P3	7E+05	fibroblast gr(	KGFLP2	9	Cytokines
KITLG	4254	KIT ligand	DCUA|DFNA69|FPH2|I	12	Cytokines
KL	9365	klotho	HFTC3	13	Cytokines

LACRT	90070	lacritin	-		12	Cytokines
LECT2	3950	leukocyte cel	chm-II|chm2		5	Cytokines
LEFTY1	10637	left-right de	LEFTB|LEFTYB		1	Cytokines
LEFTY2	7044	left-right de	EBAF|LEFTA|LEFTYA		1	Cytokines
LEP	3952	leptin	LEPD|OB|OBS		7	Cytokines
LHB	3972	luteinizing h	CGB4|HH23|LSH-B|L		19	Cytokines
LIF	3976	LIF interleuk	CDF|DIA|HILDA|MLPI		22	Cytokines
LRSAM1	90678	leucine rich	CMT2P|RIFLE|TAL		9	Cytokines
LTA	4049	lymphotoxin a	LT|TNFB|TNFSF1|TNI		6	Cytokines
LTB	4050	lymphotoxin b	TNFC|TNFSF3|TNLG1(		6	Cytokines
LTBP1	4052	latent transf	-		2	Cytokines
LTBP2	4053	latent transf	C14orf141|GLC3D|L		14	Cytokines
LTBP3	4054	latent transf	DASS|GPHYSD3|LTBP-		11	Cytokines
LTBP4	8425	latent transf	ARCL1C|LTBP-4|LTBI		19	Cytokines
MDK	4192	midkine	ARAP|MK|NEGF2		11	Cytokines
MIA	8190	MIA SH3 domai	CD-RAP		19	Cytokines
MIF	4282	macrophage mi	GIF|GLIF|MMIF		22	Cytokines
MLN	4295	motilin	-		6	Cytokines
MSTN	2660	myostatin	GDF8|MSLHP		2	Cytokines
NAMPT	10135	nicotinamide	1110035014Rik|PBEI		7	Cytokines
NDP	4693	norrin cystin	EVR2|FEVR|ND	X		Cytokines
NENF	29937	neudesin neur	CIR2|SCIRP10|SPUF		1	Cytokines
NGF	4803	nerve growth	Beta-NGF|HSAN5|NGI		1	Cytokines
NMB	4828	neuromedin B	-		15	Cytokines
NODAL	4838	nodal growth	HTX5		10	Cytokines
CCN3	4856	cellular comm	IBP-9|IGFBP-9|IGFI		8	Cytokines
NPFF	8620	neuropeptide	FMRFAL		12	Cytokines
NPPA	4878	natriuretic p	ANF|ANP|ATFB6|ATR		1	Cytokines
NPPB	4879	natriuretic p	BNP		1	Cytokines
NPPC	4880	natriuretic p	CNP|CNP2		2	Cytokines
NPY	4852	neuropeptide	PYY4		7	Cytokines
NRG1	3084	neuregulin 1	ARIA|GGF|GGF2|HGL		8	Cytokines
NRG2	9542	neuregulin 2	DON1|HRG2|NTAK		5	Cytokines
NRG3	10718	neuregulin 3	HRG3|pro-NRG3		10	Cytokines
NRG4	1E+05	neuregulin 4	HRG4		15	Cytokines
NRTN	4902	neurturin	NTN		19	Cytokines
NTF3	4908	neurotrophin	HDNF|NGF-2|NGF2|N		12	Cytokines
NTF4	4909	neurotrophin	GLC10|GLC1O|NT-4|		19	Cytokines
NTS	4922	neurotensin	NMN-125|NN|NT|NT/		12	Cytokines
NUDT6	11162	nudix hydrola	ASFGF2|FGF-AS|FGF		4	Cytokines
OGN	4969	osteoglycin	OG|OIF|SLRR3A		9	Cytokines
OSGIN1	29948	oxidative str	BDGI|OKL38		16	Cytokines
OSM	5008	oncostatin M	-		22	Cytokines
OSTN	3E+05	osteocrin	MUSCLIN		3	Cytokines
OXT	5020	oxytocin/neur	OT|OT-NPI|OXT-NPI		20	Cytokines
ENDOU	8909	endonuclease,	P11|PP11|PRSS26		12	Cytokines
PDGFA	5154	platelet deri	PDGF-A|PDGF1		7	Cytokines
PDGFB	5155	platelet deri	IBGC5|PDGF-2|PDGF		22	Cytokines
PDGFC	56034	platelet deri	FALLOTEIN|SCDGF		4	Cytokines
PDGFD	80310	platelet deri	IEGF|MSTP036|SCDGI		11	Cytokines
PDGFRA	5156	platelet deri	CD140A|PDGFR-2|PD(		4	Cytokines
PDGFRB	5159	platelet deri	CD140B|IBGC4|IMF1		5	Cytokines
PDGFRL	5157	platelet deri	PDGRL|PRLTS		8	Cytokines
PDYN	5173	prodynorphin	ADCA|PENKB|SCA23		20	Cytokines

PENK	5179	proenkephalin	PE|PENK-A	8	Cytokines
PF4	5196	platelet facto	CXCL4|PF-4|SCYB4	4	Cytokines
PF4V1	5197	platelet facto	CXCL4L1|CXCL4V1|PF	4	Cytokines
PGF	5228	placental grow	D12S1900|PGFL|PIGF	14	Cytokines
PLAU	5328	plasminogen ac	ATF|BDPLT5|QPD|UPA	10	Cytokines
PMCH	5367	pro-melanin co	MCH|ppMCH	12	Cytokines
PNOC	5368	prepronocicep	N/OFQ|NOP|OFQ|PPNO	8	Cytokines
POMC	5443	proopiomelanoc	ACTH|CLIP|LPH|MSH	2	Cytokines
PPBP	5473	pro-platelet b	B-TG1|Beta-TG|CTAP	4	Cytokines
PPBPP1	7E+05	pro-platelet b	PPBPL1|TGB2	4	Cytokines
PPBPP2	10895	pro-platelet b	PPBPL2|SPBPBP	4	Cytokines
PPY	5539	pancreatic pol	PNP|PP	17	Cytokines
PRL	5617	prolactin	GHA1	6	Cytokines
PRLH	51052	prolactin rele	PRH|PRRP	2	Cytokines
PROK1	84432	prokineticin 1	EGVEGF|PK1|PRK1	1	Cytokines
PROK2	60675	prokineticin 2	BV8|HH4|KAL4|MIT1	3	Cytokines
PSPN	5623	persephin	PSP	19	Cytokines
PTH	5741	parathyroid ho	FIH1|PTH1	11	Cytokines
PTH2	1E+05	parathyroid ho	TIP39	19	Cytokines
PTHLH	5744	parathyroid ho	BDE2|HHM|PLP|PTHR	12	Cytokines
PTN	5764	pleiotrophin	HARP|HB-GAM|HBBM|H	7	Cytokines
PYY	5697	peptide YY	PYY-I|PYY1	17	Cytokines
QRFP	3E+05	pyroglutamyla	26RFa|P518	9	Cytokines
RABEP1	9135	rabaptin, RAB	RAB5EP|RABPT5	17	Cytokines
RABEP2	79874	rabaptin, RAB	FRA	16	Cytokines
REG1A	5967	regenerating i	ICRF|P19|PSP|PSPS	2	Cytokines
RETN	56729	resistin	ADSF|FIZZ3|RETN1|R	19	Cytokines
RETNLB	84666	resistin like	FIZZ1|FIZZ2|HXCP2	3	Cytokines
RLN1	6013	relaxin 1	H1|H1RLX|RLXH1|bA	9	Cytokines
RLN2	6019	relaxin 2	H2|H2-RLX|RLXH2|bA	9	Cytokines
RLN3	1E+05	relaxin 3	H3|RXN3|ZINS4|insl	19	Cytokines
RNASE2	6036	ribonuclease A	EDN|RAF3|RNS2	14	Cytokines
S100A6	6277	S100 calcium b	2A9|5B10|CABP|CACY	1	Cytokines
SAA1	6288	serum amyloid	PIG4|SAA|SAA2|TP53	11	Cytokines
SAA2	6289	serum amyloid	SAA|SAA1	11	Cytokines
SBDS	51119	SBDS ribosome	CGI-97|SDS|SWDS	7	Cytokines
SCG2	7857	secretogranin	CHGC|EM66|SN|SgII	2	Cytokines
SCGB3A1	92304	secretoglobin	HIN-1|HIN1|LU105|P	5	Cytokines
SCT	6343	secretin	-	11	Cytokines
AIMP1	9255	aminoacyl tRNA	EMAP2|EMAPII|HLD3	4	Cytokines
SECTM1	6398	secreted and t	K12|SECTM	17	Cytokines
SEMA3A	10371	semaphorin 3A	COLL1|HH16|Hsema-I	7	Cytokines
SEMA3B	7869	semaphorin 3B	LUCA-1|SEMA5|SEMA	3	Cytokines
SEMA3C	10512	semaphorin 3C	SEMAE|SemE	7	Cytokines
SEMA3D	2E+05	semaphorin 3D	Sema-Z2|coll-2	7	Cytokines
SEMA3E	9723	semaphorin 3E	M-SEMAH|M-SemaK|SE	7	Cytokines
SEMA3F	6405	semaphorin 3F	SEMA-IV|SEMA4|SEMA	3	Cytokines
SEMA3G	56920	semaphorin 3G	sem2	3	Cytokines
SEMA4A	64218	semaphorin 4A	CORD10|RP35|SEMAB	1	Cytokines
SEMA4B	10509	semaphorin 4B	SEMAC|SemC	15	Cytokines
SEMA4C	54910	semaphorin 4C	M-SEMA-F|SEMACL1|S	2	Cytokines
SEMA4D	10507	semaphorin 4D	A8|BB18|C9orf164|C	9	Cytokines
SEMA4F	10505	ssemaphorin 4F	M-SEMA|PRO2353|S4F	2	Cytokines
SEMA4G	57715	semaphorin 4G	-	10	Cytokines

SEMA5A	9037	semaphorin 5A	SEMAF|semF	5	Cytokines
SEMA5B	54437	semaphorin 5B	SEMAG|SemG	3	Cytokines
SEMA6A	57556	semaphorin 6A	HT018|SEMA|SEMA6A1	5	Cytokines
SEMA6B	10501	semaphorin 6B	EPM11|SEM-SEMA-Y|S	19	Cytokines
SEMA6C	10500	semaphorin 6C	SEMAY|m-SemaY|m-Se	1	Cytokines
SEMA6D	80031	semaphorin 6D	-	15	Cytokines
SEMA7A	8482	semaphorin 7A	CD108|CDw108|H-SEM	15	Cytokines
SLIT1	6585	slit guidance	MEGF4|SLIL1|SLIT-1	10	Cytokines
SLIT2	9353	slit guidance	SLIL3|Slit-2	4	Cytokines
SLURP1	57152	secreted LY6/I	ANUP|ARS|ArsB|LY6-	8	Cytokines
SPP1	6696	secreted phos	BNSP|BSPI|ETA-1|OI	4	Cytokines
SST	6750	somatostatin	SMST	3	Cytokines
STC1	6781	stanniocalcin	STC	8	Cytokines
STC2	8614	stanniocalcin	STC-2|STCRP	5	Cytokines
TAC1	6863	tachykinin pr	Hs.2563|NK2|NKNA|N	7	Cytokines
TDGF1	6997	teratocarcino	CR|CR-1|CRGF|CRIPT	3	Cytokines
TDGF1P3	6998	teratocarcino	CR-3|CRIPTO|CRIPTO X		Cytokines
TG	7038	thyroglobulin	AITD3|TGN	8	Cytokines
TGFA	7039	transforming	TFGA	2	Cytokines
TGFB1	7040	transforming	CED|DPD1|IBDIMDE|I	19	Cytokines
TGFB2	7042	transforming	G-TSF|LDS4|TGF-be	1	Cytokines
TGFB3	7043	transforming	ARVD|ARVD1|LDS5|RN	14	Cytokines
THPO	7066	thrombopoieti	MGDF|MKCSF|ML|MPLI	3	Cytokines
TNC	3371	tenascin C	150-225|DFNA56|GMI	9	Cytokines
TNF	7124	tumor necrosis	DIF|TNF-alpha|TNFA	6	Cytokines
TNFRSF11	4982	TNF receptor s	OCIF|OPG|PDB5|TR1	8	Cytokines
TNFSF10	8743	TNF superfami	APO2L|Apo-2L|CD253	3	Cytokines
TNFSF11	8600	TNF superfami	CD254|ODF|OPGL|OPT	13	Cytokines
TNFSF12	8742	TNF superfami	APO3L|DR3LG|TNLG4A	17	Cytokines
TNFSF13	8741	TNF superfami	APRIL|CD256|TALL-2	17	Cytokines
TNFSF13B	10673	TNF superfami	BAFF|BLYS|CD257|DT	13	Cytokines
TNFSF14	8740	TNF superfami	CD258|HVEML|LIGHT	19	Cytokines
TNFSF15	9966	TNF superfami	TL1|TL1A|TNLG1B|VI	9	Cytokines
TNFSF18	8995	TNF superfami	AITRL|GITRL|TL6|TN	1	Cytokines
TNFSF4	7292	TNF superfami	CD134L|CD252|GP34	1	Cytokines
TNFSF8	944	TNF superfami	CD153|CD30L|CD30LG	9	Cytokines
TNFSF9	8744	TNF superfami	4-1BB-L|CD137L|TNI	19	Cytokines
TOR2A	27433	torsin family	TORP1	9	Cytokines
TRH	7200	thyrotropin r	Pro-TRH|TRF	3	Cytokines
TSHB	7252	thyroid stimu	TSH-B|TSH-BETA	1	Cytokines
TSLP	85480	thymic stroma	-	5	Cytokines
TXLNA	2E+05	taxilin alpha	IL14|TXLN	1	Cytokines
TYMP	1890	thymidine pho	ECGF|ECGF1|MEDPS1	22	Cytokines
UCN	7349	urocortin	UI|UROC	2	Cytokines
UCN2	90226	urocortin 2	SRP|UCN-II|UCNI|UI	3	Cytokines
UCN3	1E+05	urocortin 3	SCP|SPC|UCNIII	10	Cytokines
UTS2	10911	urotensin 2	PRO1068|U-II|UCN2	1	Cytokines
UTS2B	3E+05	urotensin 2B	U2B|URP|UTS2D	3	Cytokines
VEGFA	7422	vascular endo	MVCD1|VEGF|VPF	6	Cytokines
VEGFB	7423	vascular endo	VEGFL|VRF	11	Cytokines
VEGFC	7424	vascular endo	Flt4-L|LMPH1D|LMPI	4	Cytokines
VGF	7425	VGF nerve gro	SCG7|SgVII	7	Cytokines
VIP	7432	vasoactive in	PHM27	6	Cytokines
XCL1	6375	X-C motif che	ATAC|LPTN|LTN|SCM-	1	Cytokines

XCL2	6846	X-C motif che	SCM-1b|SCM1B|SCYC2	1	Cytokines
ACVR1B	91	activin A rec	ACTRIB|ACVRLK4|ALI	12	Cytokine_Receptors
ACVR1C	1E+05	activin A rec	ACVRLK7|ALK7	2	Cytokine_Receptors
ACVR2A	92	activin A rec	ACTRII|ACVR2	2	Cytokine_Receptors
ACVR2B	93	activin A rec	ACTRIIB|ActR-IIB|I	3	Cytokine_Receptors
ACVRL1	94	activin A rec	ACVRLK1|ALK-1|ALK1	12	Cytokine_Receptors
ADCYAP1R	117	ADCYAP recept	PAC1|PAC1R|PACAPR	7	Cytokine_Receptors
ADIPOR1	51094	adiponectin r	ACDCR1|CGI-45|CGI	1	Cytokine_Receptors
ADIPOR2	79602	adiponectin r	ACDCR2|PAQR2	12	Cytokine_Receptors
ADRB1	153	adrenoceptor	ADRB1R|B1AR|BETA1	10	Cytokine_Receptors
ADRB2	154	adrenoceptor	ADRB2R|ADRBR|B2AR	5	Cytokine_Receptors
AGTR1	185	angiotensin I	AG2S|AGTR1B|AT1|A	3	Cytokine_Receptors
AGTR2	186	angiotensin I	AT2|ATGR2|MRX88	X	Cytokine_Receptors
AMHR2	269	anti-Mulleria	AMHR|MISR2|MISRII	12	Cytokine_Receptors
ANGPT1	284	angiopoietin	AGP1|AGPT|ANG1	8	Cytokine_Receptors
ANGPT4	51378	angiopoietin	ANG3|ANG4	20	Cytokine_Receptors
ANGPTL1	9068	angiopoietin	ANG3|ANGPT3|ARP1|	1	Cytokine_Receptors
ANGPTL2	23452	angiopoietin	ARP2|HARP	9	Cytokine_Receptors
ANGPTL3	27329	angiopoietin	ANG-5|ANGPT5|ANL3	1	Cytokine_Receptors
ANGPTL4	51129	angiopoietin	ARP4|FIAF|HARP|HF	19	Cytokine_Receptors
ANGPTL6	83854	angiopoietin	AGF|ARP5	19	Cytokine_Receptors
APLNR	187	apelin recept	AGTRL1|APJ|APJR|H	11	Cytokine_Receptors
AR	367	androgen rece	AIS|AR8|DHTR|HUMA	X	Cytokine_Receptors
AVPR1A	552	arginine vaso	AVPR V1a|AVPR1|V1a	12	Cytokine_Receptors
AVPR1B	553	arginine vaso	AVPR3|V1bR	1	Cytokine_Receptors
AVPR2	554	arginine vaso	ADHR|DI1|DIR|DIR3	X	Cytokine_Receptors
BMPR1A	657	bone morphoge	10q23del|ACVRLK3|	10	Cytokine_Receptors
BMPR1B	658	bone morphoge	ALK-6|ALK6|AMDD|BI	4	Cytokine_Receptors
BMPR2	659	bone morphoge	BMPR-II|BMPR3|BMR2	2	Cytokine_Receptors
BRD8	10902	bromodomain c	SMAP|SMAP2|p120	5	Cytokine_Receptors
C3AR1	719	complement C3	AZ3B|C3AR|HNFAG09	12	Cytokine_Receptors
C5AR1	728	complement C5	C5A|C5AR|C5R1|CD8	19	Cytokine_Receptors
CALCR	799	calcitonin re	CRT|CT-R|CTR|CTR1	7	Cytokine_Receptors
CALCRL	10203	calcitonin re	CGRPR|CRLR|LMPHM8	2	Cytokine_Receptors
ACKR2	1238	atypical chem	CCBP2|CCR10|CCR9|C	3	Cytokine_Receptors
CCR1	1230	C-C motif che	CD191|CKR-1|CKR1|C	3	Cytokine_Receptors
CCR10	2826	C-C motif che	GPR2	17	Cytokine_Receptors
CCR3	1232	C-C motif che	C C CKR3|CC-CKR-3	3	Cytokine_Receptors
CCR4	1233	C-C motif che	CC-CKR-4|CD194|CKI	3	Cytokine_Receptors
CCR5	1234	C-C motif che	CC-CKR-5|CCCKR5|CC	3	Cytokine_Receptors
CCR6	1235	C-C motif che	BN-1|C-C CKR-6|CC-	6	Cytokine_Receptors
CCR7	1236	C-C motif che	BLR2|CC-CKR-7|CCR-	17	Cytokine_Receptors
CCR8	1237	C-C motif che	CC-CKR-8|CCR-8|CDw	3	Cytokine_Receptors
CCR9	10803	C-C motif che	CC-CKR-9|CDw199|GI	3	Cytokine_Receptors
ACKR4	51554	atypical chem	CC-CKR-11|CCBP2|CC	3	Cytokine_Receptors
CCRL2	9034	C-C motif che	ACKR5|CKRX|CRAM|CI	3	Cytokine_Receptors
CD40	958	CD40 molecule	Bp50|CDW40|TNFRSF5	20	Cytokine_Receptors
CMKLR1	1240	chemerin chem	CHEMERINR|ChemR23	12	Cytokine_Receptors
CNTFR	1271	ciliary neuro	-	9	Cytokine_Receptors
CRHR1	1394	corticotropin	CRF-R|CRF-R-1|CRF-	17	Cytokine_Receptors
CRHR2	1395	corticotropin	CRF-RB|CRF2|CRFR2	7	Cytokine_Receptors
CRIM1	51232	cysteine rich	CRIM-1|S52	2	Cytokine_Receptors
CRLF1	9244	cytokine rece	CISS|CISS1|CLF|CLI	19	Cytokine_Receptors
CRLF2	64109	cytokine rece	CRL2|CRLF2Y|TSLPR	X|Y	Cytokine_Receptors

CRLF3	51379	cytokine rece	CREME-9|CREME9|CRI	17	Cytokine_Receptors
CSF1R	1436	colony stimula	BANDDOS|C-FMS|CD11	5	Cytokine_Receptors
CSF2RA	1438	colony stimula	CD116|CDw116|CSF2I	X|Y	Cytokine_Receptors
CSF2RB	1439	colony stimula	CD131|CDw131|IL3RI	22	Cytokine_Receptors
CSF3R	1441	colony stimula	CD114|GCSFR|SCN7	1	Cytokine_Receptors
CX3CR1	1524	C-X3-C motif (	CCRL1|CMKBRL1|CMKI	3	Cytokine_Receptors
CXCR3	2833	C-X-C motif cl	CD182|CD183|CKR-L2	X	Cytokine_Receptors
CXCR4	7852	C-X-C motif cl	CD184|D2S201E|FB22	2	Cytokine_Receptors
CXCR5	643	C-X-C motif cl	BLR1|CD185|MDR15	11	Cytokine_Receptors
CXCR6	10663	C-X-C motif cl	BONZO|CD186|STRL33	3	Cytokine_Receptors
ACKR3	57007	atypical chem	CMKOR1|CXC-R7|CXCI	2	Cytokine_Receptors
CYSLTR1	10800	cysteinyl leu	CYSLT1|CYSLT1R|CYS	X	Cytokine_Receptors
CYSLTR2	57105	cysteinyl leu	CYSLT2|CYSLT2R|GP(	13	Cytokine_Receptors
ACKR1	2532	atypical chem	CCBP1|CD234|DARC|I	1	Cytokine_Receptors
EDNRA	1909	endothelin re	ET-A|ETA|ETA-R|ET/	4	Cytokine_Receptors
EDNRB	1910	endothelin re	ABCDS|ET-B|ET-BR|I	13	Cytokine_Receptors
EGFR	1956	epidermal gro	ERBB|ERBB1|HER1|N	7	Cytokine_Receptors
ENG	2022	endoglin	END|HHT1|ORW1	9	Cytokine_Receptors
EPOR	2057	erythropoieti	EPO-R	19	Cytokine_Receptors
ESR1	2099	estrogen rece	ER|ESR|ESRA|ESTRR	6	Cytokine_Receptors
ESR2	2100	estrogen rece	ER-BETA|ESR-BETA|I	14	Cytokine_Receptors
ESRRA	2101	estrogen rela	ERR1|ERRa|ERRalpha	11	Cytokine_Receptors
ESRRB	2103	estrogen rela	DFNB35|ERR beta-2	14	Cytokine_Receptors
ESRRG	2104	estrogen rela	ERR-gamma|ERR3|ERI	1	Cytokine_Receptors
FGFR1	2260	fibroblast gr	BFGFR|CD331|CEK|E(	8	Cytokine_Receptors
FGFR2	2263	fibroblast gr	BBDS|BEK|BFR-1|CD3	10	Cytokine_Receptors
FGFR3	2261	fibroblast gr	ACH|CD333|CEK2|HSI	4	Cytokine_Receptors
FGFR4	2264	fibroblast gr	CD334|JTK2|TKF	5	Cytokine_Receptors
FGFRL1	53834	fibroblast gr	FGFR-5|FGFR5|FHFR	4	Cytokine_Receptors
FLT1	2321	fms related r	FLT|FLT-1|VEGFR-1	13	Cytokine_Receptors
FLT3	2322	fms related r	CD135|FLK-2|FLK2|S	13	Cytokine_Receptors
FLT4	2324	fms related r	CHTD7|FLT-4|FLT41	5	Cytokine_Receptors
FPR1	2357	formyl peptid	FMLP|FPR	19	Cytokine_Receptors
FPR2	2358	formyl peptid	ALXR|FMLP-R-II|FMI	19	Cytokine_Receptors
FPR2	2358	formyl peptid	ALXR|FMLP-R-II|FMI	19	Cytokine_Receptors
FSHR	2492	follicle stim	FSHR1|FSHRO|LGR1|(	2	Cytokine_Receptors
GALR2	8811	galanin recep	GAL2-R|GALNR2|GALI	17	Cytokine_Receptors
GALR3	8484	galanin recep	-	22	Cytokine_Receptors
GCGR	2642	glucagon rece	GGR|GL-R	17	Cytokine_Receptors
GHR	2690	growth hormon	GHBP|GHIP	5	Cytokine_Receptors
GHRHR	2692	growth hormon	GHRFR|GRFR|IGHD1B	7	Cytokine_Receptors
GHSR	2693	growth hormon	GHDP	3	Cytokine_Receptors
GIPR	2696	gastric inhib	PGQTL2	19	Cytokine_Receptors
GLP1R	2740	glucagon like	GLP-1|GLP-1-R|GLP-	6	Cytokine_Receptors
GLP2R	9340	glucagon like	-	17	Cytokine_Receptors
GNRHR	2798	gonadotropin	GNRHR1|GRHR|HH7|LI	4	Cytokine_Receptors
GPER1	2852	G protein-cou	CEPR|CMKRL2|DRY12	7	Cytokine_Receptors
GPR17	2840	G protein-cou	-	2	Cytokine_Receptors
GPR32	2854	G protein-cou	RVDR1	19	Cytokine_Receptors
GPR33	2856	G protein-cou	-	14	Cytokine_Receptors
PTGDR2	11251	prostaglandin	CD294|CRTH2|DL1R|I	11	Cytokine_Receptors
C5AR2	27202	complement co	C5L2|GPF77|GPR77	19	Cytokine_Receptors
HNF4A	3172	hepatocyte nu	FRTS4|HNF4|HNF4a7	20	Cytokine_Receptors
HNF4G	3174	hepatocyte nu	NR2A2|NR2A3	8	Cytokine_Receptors

HTR3A	3359	5-hydroxytryp	5-HT-3|5-HT3A|5-H'	11	Cytokine_Receptors
HTR3B	9177	5-hydroxytryp	5-HT3B	11	Cytokine_Receptors
HTR3C	2E+05	5-hydroxytryp	-	3	Cytokine_Receptors
HTR3D	2E+05	5-hydroxytryp	5HT3D	3	Cytokine_Receptors
HTR3E	3E+05	5-hydroxytryp	5-HT3-E|5-HT3E|5-H	3	Cytokine_Receptors
IFNAR1	3454	interferon al	AVP|IFN-alpha-REC	21	Cytokine_Receptors
IFNAR2	3455	interferon al	IFN-R|IFN-alpha-RI	21	Cytokine_Receptors
IFNGR1	3459	interferon ga	CD119|IFNGR|IMD27	6	Cytokine_Receptors
IFNGR2	3460	interferon ga	AF-1|IFGR2|IFNGT1	21	Cytokine_Receptors
IGF1R	3480	insulin like	CD221|IGFIR|IGFR|	15	Cytokine_Receptors
IGF2R	3482	insulin like	CD222|CI-M6PR|CIMI	6	Cytokine_Receptors
IL10RA	3587	interleukin 1	CD210|CD210a|CDW2	11	Cytokine_Receptors
IL10RB	3588	interleukin 1	CDW210B|CRF2-4|CRI	21	Cytokine_Receptors
IL11RA	3590	interleukin 1	CRSDA	9	Cytokine_Receptors
IL12RB1	3594	interleukin 1	CD212|IL-12R-BETA	19	Cytokine_Receptors
IL12RB2	3595	interleukin 1	-	1	Cytokine_Receptors
IL13RA1	3597	interleukin 1	CD213A1|CT19|IL-1	X	Cytokine_Receptors
IL13RA2	3598	interleukin 1	CD213A2|CT19|IL-1	X	Cytokine_Receptors
IL15RA	3601	interleukin 1	CD215	10	Cytokine_Receptors
IL2RB	3560	interleukin 2	CD122|IL15RB|IMD6	22	Cytokine_Receptors
IL17RA	23765	interleukin 1	CANDF5|CD217|CDw2	22	Cytokine_Receptors
IL17RB	55540	interleukin 1	CRL4|EVI27|IL17BR	3	Cytokine_Receptors
IL17RC	84818	interleukin 1	CANDF9|IL17-RL|IL	3	Cytokine_Receptors
IL17RD	54756	interleukin 1	HH18|IL-17RD|IL17I	3	Cytokine_Receptors
IL17RE	1E+05	interleukin 1	-	3	Cytokine_Receptors
IL18R1	8809	interleukin 1	CD218a|CDw218a|IL-	2	Cytokine_Receptors
IL18RAP	8807	interleukin 1	ACPL|CD218b|CDw21	2	Cytokine_Receptors
IL1R1	3554	interleukin 1	CD121A|D2S1473|IL-	2	Cytokine_Receptors
IL1R2	7850	interleukin 1	CD121b|CDw121b|IL-	2	Cytokine_Receptors
IL1RAP	3556	interleukin 1	C3orf13|IL-1RAcP|	3	Cytokine_Receptors
IL1RL1	9173	interleukin 1	DER4|FIT-1|IL33R|	2	Cytokine_Receptors
IL1RL2	8808	interleukin 1	IL-1Rrp2|IL-36R|II	2	Cytokine_Receptors
IL20RA	53832	interleukin 2	CRF2-8|IL-20R-alpl	6	Cytokine_Receptors
IL20RB	53833	interleukin 2	DIRS1|FNDC6|IL-20I	3	Cytokine_Receptors
IL21R	50615	interleukin 2	CD360|IMD56|NILR	16	Cytokine_Receptors
IL22RA1	58985	interleukin 2	CRF2-9|IL22R|IL22I	1	Cytokine_Receptors
IL22RA2	1E+05	interleukin 2	CRF2-10|CRF2-S1|CI	6	Cytokine_Receptors
IL23R	1E+05	interleukin 2	-	1	Cytokine_Receptors
IL27RA	9466	interleukin 2	CRL1|IL-27RA|IL27I	19	Cytokine_Receptors
IFNLR1	2E+05	interferon la	CRF2/12|IFNLR|IL-	1	Cytokine_Receptors
IL2RA	3559	interleukin 2	CD25|IDDM10|IL2R|	10	Cytokine_Receptors
IL2RB	3560	interleukin 2	CD122|IL15RB|IMD6	22	Cytokine_Receptors
IL2RG	3561	interleukin 2	CD132|CIDX|IL-2RG	X	Cytokine_Receptors
IL31RA	1E+05	interleukin 3	CRL|CRL3|GLM-R|GLI	5	Cytokine_Receptors
IL3RA	3563	interleukin 3	CD123|IL3R|IL3RAY	X|Y	Cytokine_Receptors
IL4R	3566	interleukin 4	CD124|IL-4RA|IL4R	16	Cytokine_Receptors
IL5RA	3568	interleukin 5	CD125|CDw125|HSIL	3	Cytokine_Receptors
IL6R	3570	interleukin 6	CD126|IL-6R-1|IL-	1	Cytokine_Receptors
IL7R	3575	interleukin 7	CD127|CDW127|IL-7I	5	Cytokine_Receptors
CXCR1	3577	C-X-C motif c	C-C|C-C-CKR-1|CD1	2	Cytokine_Receptors
CXCR2	3579	C-X-C motif c	CD182|CDw128b|CMK	2	Cytokine_Receptors
IL9R	3581	interleukin 9	CD129|IL-9R	X|Y	Cytokine_Receptors
INSR	3643	insulin recep	CD220|HHF5	19	Cytokine_Receptors
KDR	3791	kinase insert	CD309|FLK1|VEGFR|	4	Cytokine_Receptors

LEPR	3953	leptin recept(	CD295|LEP-R|LEPRD	1	Cytokine_Receptors
LGR4	55366	leucine rich	BNMD17|GPR48	11	Cytokine_Receptors
LGR5	8549	leucine rich	FEX|GPR49|GPR67|GI	12	Cytokine_Receptors
LGR6	59352	leucine rich	GPCR|VTS20631	1	Cytokine_Receptors
LHCGR	3973	luteinizing h	HHG|LCGR|LGR2|LH/(	2	Cytokine_Receptors
LIFR	3977	LIF receptor	CD118|LIF-R|SJS2|S	5	Cytokine_Receptors
LTB4R	1241	leukotriene B	BLT1|BLTR|CMKRL1|(	14	Cytokine_Receptors
LTB4R2	56413	leukotriene B	BLT2|BLTR2|JULF2|I	14	Cytokine_Receptors
LTBR	4055	lymphotoxin b	D12S370|LT-BETA-R	12	Cytokine_Receptors
MC1R	4157	melanocortin	CMM5|MSH-R|SHEP2	16	Cytokine_Receptors
MC2R	4158	melanocortin	ACTHR	18	Cytokine_Receptors
MC3R	4159	melanocortin	BMIQ9|MC3|MC3-R|OI	20	Cytokine_Receptors
MC4R	4160	melanocortin	BMIQ20	18	Cytokine_Receptors
MCHR1	2847	melanin conce	GPR24|MCH-1R|MCH1I	22	Cytokine_Receptors
MCHR2	84539	melanin conce	GPR145|GPRv17|MCH-	6	Cytokine_Receptors
MET	4233	MET proto-onc	AUTS9|DFNB97|HGFR	7	Cytokine_Receptors
MLNR	2862	motilin recep	GPR38|MTLR1	13	Cytokine_Receptors
MPL	4352	MPL proto-onc	C-MPL|CD110|MPLV|	1	Cytokine_Receptors
MTNR1A	4543	melatonin rec	MEL-1A-R|MT1	4	Cytokine_Receptors
MTNR1B	4544	melatonin rec	FGQTL2|MEL-1B-R|M	11	Cytokine_Receptors
NGFR	4804	nerve growth	CD271|Gp80-LNGFR|	17	Cytokine_Receptors
NMBR	4829	neuromedin B	BB1|BB1R|NMB-R	6	Cytokine_Receptors
NPR1	4881	natriuretic p	ANPRA|ANPa|GUC2A|(	1	Cytokine_Receptors
NPR3	4883	natriuretic p	ANP-C|ANPR-C|ANPR(	5	Cytokine_Receptors
NR0B1	190	nuclear recep	AHC|AHCH|AHX|DAX- X		Cytokine_Receptors
NR0B2	8431	nuclear recep	SHP|SHP1	1	Cytokine_Receptors
NR1D1	9572	nuclear recep	EAR1|REVERBA|REVEI	17	Cytokine_Receptors
NR1D2	9975	nuclear recep	BD73|EAR-1R|REVERI	3	Cytokine_Receptors
NR1H2	7376	nuclear recep	LXR-b|LXRB|NER|NEI	19	Cytokine_Receptors
NR1H3	10062	nuclear recep	LXR-a|LXRA|RLD-1	11	Cytokine_Receptors
NR1H4	9971	nuclear recep	BAR|FXR|HRR-1|HRR	12	Cytokine_Receptors
NR1I2	8856	nuclear recep	BXR|ONR1|PAR|PAR1	3	Cytokine_Receptors
NR1I3	9970	nuclear recep	CAR|CAR1|MB67	1	Cytokine_Receptors
NR2C1	7181	nuclear recep	TR2	12	Cytokine_Receptors
NR2C2	7182	nuclear recep	TAK1|TR4	3	Cytokine_Receptors
NR2E1	7101	nuclear recep	TLL|TLX|XTLL	6	Cytokine_Receptors
NR2E3	10002	nuclear recep	ESCS|PNR|RNR|RP37	15	Cytokine_Receptors
NR2F1	7025	nuclear recep	BBOAS|BBSOAS|COUP-	5	Cytokine_Receptors
NR2F2	7026	nuclear recep	ARP-1|ARP1|CHTD4|(	15	Cytokine_Receptors
NR2F6	2063	nuclear recep	EAR-2|EAR2|ERBAL2	19	Cytokine_Receptors
NR3C1	2908	nuclear recep	GCCR|GCR|GCRST|GR	5	Cytokine_Receptors
NR3C2	4306	nuclear recep	MCR|MLR|MR|NR3C2V	4	Cytokine_Receptors
NR4A1	3164	nuclear recep	GFRP1|HMR|N10|NAK-	12	Cytokine_Receptors
NR4A2	4929	nuclear recep	HZF-3|NOT|NURR1|RI	2	Cytokine_Receptors
NR4A3	8013	nuclear recep	CHN|CSMF|MINOR|NOI	9	Cytokine_Receptors
NR5A1	2516	nuclear recep	AD4BP|ELP|FTZ1|FT	9	Cytokine_Receptors
NR5A2	2494	nuclear recep	B1F|B1F2|CPF|FTF|I	1	Cytokine_Receptors
NR6A1	2649	nuclear recep	CT150|GCNF|GCNF1|I	9	Cytokine_Receptors
NRP1	8829	neuropilin 1	BDCA4|CD304|NP1|NI	10	Cytokine_Receptors
NRP2	8828	neuropilin 2	NP2|NPN2|PRO2714|	2	Cytokine_Receptors
OGFR	11054	opioid growth	-	20	Cytokine_Receptors
OPRD1	4985	opioid recept(	DOP|DOR|DOR1|OPRD	1	Cytokine_Receptors
OPRK1	4986	opioid recept(	K-OR-1|KOP|KOR|KOI	8	Cytokine_Receptors
OPRL1	4987	opioid relate(	KOR-3|KOR3|NOCIR|I	20	Cytokine_Receptors

OPRM1	4988	opioid recept	LMOR|M-OR-1|MOP|M(	6	Cytokine_Receptors
OSMR	9180	oncostatin M	IL-31R-beta|IL-31I	5	Cytokine_Receptors
OXTR	5021	oxytocin rece	OT-R	3	Cytokine_Receptors
PGR	5241	progesterone	NR3C3|PR	11	Cytokine_Receptors
PGRMC2	10424	progesterone	DG6|PMBP	4	Cytokine_Receptors
PLAUR	5329	plasminogen a	CD87|U-PAR|UPAR|UI	19	Cytokine_Receptors
PLXNA1	5361	plexin A1	NOV|NOVP|PLEXIN-A	3	Cytokine_Receptors
PLXNA2	5362	plexin A2	OCT|PLXN2	1	Cytokine_Receptors
PLXNA3	55558	plexin A3	6.3|HSSEXGENE|PLXI	X	Cytokine_Receptors
PLXNA4	91584	plexin A4	FAYV2820|PLEXA4|PI	7	Cytokine_Receptors
PLXNB1	5364	plexin B1	PLEXIN-B1|PLXN5|SI	3	Cytokine_Receptors
PLXNB2	23654	plexin B2	MM1|Nbla00445|PLE	22	Cytokine_Receptors
PLXNB3	5365	plexin B3	PLEXB3|PLEXR|PLXN(	X	Cytokine_Receptors
PLXNC1	10154	plexin C1	CD232|PLXN-C1|VESI	12	Cytokine_Receptors
PLXND1	23129	plexin D1	PLEXD1	3	Cytokine_Receptors
PPARA	5465	peroxisome pr	NR1C1|PPAR|PPARal	22	Cytokine_Receptors
PPARD	5467	peroxisome pr	FAAR|NR1C2|NUC1|NU	6	Cytokine_Receptors
PPARG	5468	peroxisome pr	CIMT1|GLM1|NR1C3|I	3	Cytokine_Receptors
PRLHR	2834	prolactin rel	GPR10|GR3|PrRPR	10	Cytokine_Receptors
PRLR	5618	prolactin rec	HPRL|MFAB|RI-PRLR	5	Cytokine_Receptors
PTAFR	5724	platelet acti	PAFR	1	Cytokine_Receptors
PTGDR	5729	prostaglandin	AS1|ASRT1|DP|DP1|I	14	Cytokine_Receptors
PTGDS	5730	prostaglandin	L-PGDS|LPGDS|PDS|I	9	Cytokine_Receptors
PTGER1	5731	prostaglandin	EP1	19	Cytokine_Receptors
PTGER2	5732	prostaglandin	EP2	14	Cytokine_Receptors
PTGER3	5733	prostaglandin	EP3|EP3-I|EP3-II|I	1	Cytokine_Receptors
PTGER4	5734	prostaglandin	EP4|EP4R	5	Cytokine_Receptors
PTGFR	5737	prostaglandin	FP	1	Cytokine_Receptors
PTH1R	5745	parathyroid h	EKNS|PFE|PTHR|PTHI	3	Cytokine_Receptors
PTH2R	5746	parathyroid h	PTHR2	2	Cytokine_Receptors
RARA	5914	retinoic acid	NR1B1|RAR	17	Cytokine_Receptors
RARB	5915	retinoic acid	HAP|MCOPS12|NR1B2	3	Cytokine_Receptors
RARG	5916	retinoic acid	NR1B3|RARC	12	Cytokine_Receptors
ROBO1	6091	roundabout gu	DUTT1|SAX3	3	Cytokine_Receptors
ROBO2	6092	roundabout gu	SAX3	3	Cytokine_Receptors
ROBO3	64221	roundabout gu	HGPPS|HGPPS1|HGPS	11	Cytokine_Receptors
RORA	6095	RAR related o	IDDECA|NR1F1|ROR1	15	Cytokine_Receptors
RORB	6096	RAR related o	EIG15|NR1F2|ROR-BI	9	Cytokine_Receptors
RORC	6097	RAR related o	IMD42|NR1F3|RORG|I	1	Cytokine_Receptors
RXFP1	59350	relaxin famil	LGR7|RXFPR1	4	Cytokine_Receptors
RXFP2	1E+05	relaxin famil	GPR106|GREAT|INSL	13	Cytokine_Receptors
RXFP3	51289	relaxin famil	GPCR135|RLN3R1|RXI	5	Cytokine_Receptors
RXRA	6256	retinoid X re	NR2B1	9	Cytokine_Receptors
RXRB	6257	retinoid X re	DAUDI6|H-2RIIBP|NI	6	Cytokine_Receptors
RXRG	6258	retinoid X re	NR2B3|RXRC	1	Cytokine_Receptors
S1PR1	1901	sphingosine-1	CD363|CHEDG1|D1S3	1	Cytokine_Receptors
S1PR2	9294	sphingosine-1	AGR16|DFNB68|EDG-	19	Cytokine_Receptors
SCTR	6344	secretin rece	SR	2	Cytokine_Receptors
SDC1	6382	syndecan 1	CD138|SDC|SYND1|s	2	Cytokine_Receptors
SDC2	6383	syndecan 2	CD362|HSPG|HSPG1|	8	Cytokine_Receptors
SDC3	9672	syndecan 3	SDCN|SYND3	1	Cytokine_Receptors
SDC4	6385	syndecan 4	SYND4	20	Cytokine_Receptors
SORT1	6272	sortilin 1	Gp95|LDLCQ6|NT3|N	1	Cytokine_Receptors
SSTR1	6751	somatostatin	SRIF-2|SS-1-R|SS1-	14	Cytokine_Receptors

SSTR2	6752	somatostatin r	-	17 Cytokine_Receptors
SSTR5	6755	somatostatin r	SS-5-R	16 Cytokine_Receptors
ST2	6761	-	-	11 Cytokine_Receptors
TACR1	6869	tachykinin rec	NK1R|NKIR|SPR|TAC1	2 Cytokine_Receptors
TEK	7010	TEK receptor t	CD202B|GLC3E|TIE-2	9 Cytokine_Receptors
TGFBR1	7046	transforming g	AAT5|ACVRLK4|ALK-5	9 Cytokine_Receptors
TGFBR2	7048	transforming g	AAT3|FAA3|LDS1B|LI	3 Cytokine_Receptors
TGFBR3	7049	transforming g	BGCAN|betaglycan	1 Cytokine_Receptors
THRA	7067	thyroid hormon	AR7|CHNG6|EAR7|ERI	17 Cytokine_Receptors
THRB	7068	thyroid hormon	C-ERBA-2|C-ERBA-BI	3 Cytokine_Receptors
TIE1	7075	tyrosine kinas	JTK14|TIE	1 Cytokine_Receptors
TNFRSF10	8797	TNF receptor s	APO2|CD261|DR4|TRA	8 Cytokine_Receptors
TNFRSF10	8795	TNF receptor s	CD262|DR5|KILLER|I	8 Cytokine_Receptors
TNFRSF10	8794	TNF receptor s	CD263|DCR1|DCR1-TI	8 Cytokine_Receptors
TNFRSF10	8793	TNF receptor s	CD264|DCR2|TRAIL-I	8 Cytokine_Receptors
TNFRSF11	8792	TNF receptor s	CD265|FEO|LOH18CR1	18 Cytokine_Receptors
TNFRSF12	51330	TNF receptor s	CD266|FN14|TWEAKR	16 Cytokine_Receptors
TNFRSF13	23495	TNF receptor s	CD267|CVID|CVID2|I	17 Cytokine_Receptors
TNFRSF13	1E+05	TNF receptor s	BAFF-R|BAFFR|BROMI	22 Cytokine_Receptors
TNFRSF14	8764	TNF receptor s	ATAR|CD270|HVEA|HV	1 Cytokine_Receptors
TNFRSF17	608	TNF receptor s	BCM|BCMA|CD269|TNI	16 Cytokine_Receptors
TNFRSF18	8784	TNF receptor s	AITR|CD357|ENERGEI	1 Cytokine_Receptors
TNFRSF19	55504	TNF receptor s	TAJ|TAJ-alpha|TRAI	13 Cytokine_Receptors
TNFRSF1A	7132	TNF receptor s	CD120a|FPF|TBP1|TI	12 Cytokine_Receptors
TNFRSF1B	7133	TNF receptor s	CD120b|TBPII|TNF-I	1 Cytokine_Receptors
TNFRSF21	27242	TNF receptor s	BM-018|CD358|DR6	6 Cytokine_Receptors
TNFRSF25	8718	TNF receptor s	APO-3|DDR3|DR3|GEI	1 Cytokine_Receptors
TNFRSF4	7293	TNF receptor s	ACT35|CD134|IMD16	1 Cytokine_Receptors
TNFRSF6B	8771	TNF receptor s	DCR3|DJ583P15.1.1	20 Cytokine_Receptors
TNFRSF8	943	TNF receptor s	CD30|D1S166E|Ki-1	1 Cytokine_Receptors
TNFRSF9	3604	TNF receptor s	4-1BB|CD137|CDw137	1 Cytokine_Receptors
TRHR	7201	thyrotropin re	CHNG7|TRH-R	8 Cytokine_Receptors
TSHR	7253	thyroid stimul	CHNG1|LGR3|hTSHR-I	14 Cytokine_Receptors
TUBB3	10381	tubulin beta 3	CDCBM|CDCBM1|CFEOM	16 Cytokine_Receptors
VDR	7421	vitamin D rece	NR1I1|PPP1R163	12 Cytokine_Receptors
VIPR1	7433	vasoactive int	HVR1|II|PACAP-R-2	3 Cytokine_Receptors
VIPR2	7434	vasoactive int	C16DUPq36.3|DUP7q3	7 Cytokine_Receptors
XCR1	2829	X-C motif chem	CCXCR1|GPR5	3 Cytokine_Receptors
IFNA10	3446	interferon alp	IFN-alphaC	9 Interferons
IFNA13	3447	interferon alp	-	9 Interferons
IFNA14	3448	interferon alp	IFN-alphaH|LEIF2H	9 Interferons
IFNA16	3449	interferon alp	IFN-alpha-16|IFN-a	9 Interferons
IFNA17	3451	interferon alp	IFN-alphaI|IFNA|IN	9 Interferons
IFNA2	3440	interferon alp	IFN-alpha-2|IFN-al	9 Interferons
IFNA21	3452	interferon alp	IFN-alphaI|LeIF F	9 Interferons
IFNA4	3441	interferon alp	IFN-alpha4a|INFA4	9 Interferons
IFNA5	3442	interferon alp	IFN-alpha-5|IFN-al	9 Interferons
IFNA6	3443	interferon alp	IFN-alphaK	9 Interferons
IFNA7	3444	interferon alp	IFN-alphaJ|IFNA-J	9 Interferons
IFNA8	3445	interferon alp	IFN-alphaB	9 Interferons
IFNB1	3456	interferon bet	IFB|IFF|IFN-beta|I	9 Interferons
IFNE	3E+05	interferon eps	IFN-E|IFNE1|IFNT1	9 Interferons
IFNG	3458	interferon gam	IFG|IFI	12 Interferons
IFNK	56832	interferon kap	IFNT1|INFE1	9 Interferons

IFNW1	3467	interferon om(	-	9	Interferons
IFNAR2	3455	interferon al|	IFN-R|IFN-alpha-RI	21	Interferon_Receptor
IFNGR1	3459	interferon gar	CD119|IFNGR|IMD27/	6	Interferon_Receptor
IFNGR2	3460	interferon gar	AF-1|IFGR2|IFNGT1	21	Interferon_Receptor
IL11	3589	interleukin 1	AGIF|IL-11	19	Interleukins
IL12A	3592	interleukin 1:	CLMF|IL-12A|NFSK|N	3	Interleukins
IL12B	3593	interleukin 1:	CLMF|CLMF2|IL-12B	5	Interleukins
IL13	3596	interleukin 1:	IL-13|P600	5	Interleukins
IL15	3600	interleukin 1!	IL-15	4	Interleukins
IL16	3603	interleukin 1(	LCF|NIL16|PRIL16|p	15	Interleukins
IL17A	3605	interleukin 1'	CTLA-8|CTLA8|IL-1'	6	Interleukins
IL17B	27190	interleukin 1'	IL-17B|IL-20|NIRF	5	Interleukins
IL17C	27189	interleukin 1'	CX2|IL-17C	16	Interleukins
IL17D	53342	interleukin 1'	IL-17D	13	Interleukins
IL17F	1E+05	interleukin 1'	CANDF6|IL-17F|ML-1	6	Interleukins
IL18	3606	interleukin 18	IGIF|IL-18|IL-1g|1	11	Interleukins
IL19	29949	interleukin 1!	IL-10C|MDA1|NG.1|Z	1	Interleukins
IL1A	3552	interleukin 1	IL-1 alpha|IL-1A|1	2	Interleukins
IL1B	3553	interleukin 1	IL-1|IL1-BETA|IL1I	2	Interleukins
IL1F10	84639	interleukin 1	FIL1-theta|FKSG75	2	Interleukins
IL36RN	26525	interleukin 3(	FIL1|FIL1(DELTA)|I	2	Interleukins
IL36A	27179	interleukin 3(	FIL1|FIL1(EPSILON)	2	Interleukins
IL37	27178	interleukin 3'	FIL1|FIL1(ZETA)|F1	2	Interleukins
IL36B	27177	interleukin 3(	FIL1|FIL1-(ETA)|F1	2	Interleukins
IL36G	56300	interleukin 3(	IL-1F9|IL-1H1|IL-1	2	Interleukins
IL1RN	3557	interleukin 1	DIRA|ICIL-1RA|IL-1	2	Interleukins
IL2	3558	interleukin 2	IL-2|TCGF|lymphok:	4	Interleukins
IL20	50604	interleukin 2(	IL-20|IL10D|ZCYTO1	1	Interleukins
IL21	59067	interleukin 2	CVID11|IL-21|Za11	4	Interleukins
IL22	50616	interleukin 2:	IL-21|IL-22|IL-D1	12	Interleukins
IL23A	51561	interleukin 2:	IL-23|IL-23A|IL23I	12	Interleukins
IL24	11009	interleukin 2	C49A|FISP|IL10B|MI	1	Interleukins
IL25	64806	interleukin 2!	IL17E	14	Interleukins
IL26	55801	interleukin 2(	AK155|IL-26	12	Interleukins
IL27	2E+05	interleukin 2'	IL-27|IL-27A|IL27/	16	Interleukins
IFNL2	3E+05	interferon lar	IL-28A|IL28A	19	Interleukins
IFNL3	3E+05	interferon lar	IFN-lambda-3|IFN-1	19	Interleukins
IFNL1	3E+05	interferon lar	IL-29|IL29	19	Interleukins
IL3	3562	interleukin 3	IL-3|MCGF|MULTI-CS	5	Interleukins
IL31	4E+05	interleukin 3	IL-31	12	Interleukins
IL32	9235	interleukin 3:	IL-32alpha|IL-32be	16	Interleukins
IL33	90865	interleukin 3:	C9orf26|DVS27|IL1I	9	Interleukins
IL34	1E+05	interleukin 3	C16orf77|IL-34	16	Interleukins
IL4	3565	interleukin 4	BCGF-1|BCGF1|BSF-1	5	Interleukins
IL5	3567	interleukin 5	EDF|IL-5|TRF	5	Interleukins
IL6	3569	interleukin 6	BSF-2|BSF2|CDF|HGI	7	Interleukins
IL6ST	3572	interleukin 6	CD130|CDW130|GP13(	5	Interleukins
IL7	3574	interleukin 7	IL-7	8	Interleukins
CXCL8	3576	C-X-C motif cl	GCP-1|GCP1|IL8|LE(	4	Interleukins
IL9	3578	interleukin 9	HP40|IL-9|P40	5	Interleukins
TXLNA	2E+05	taxilin alpha	IL14|TXLN	1	Interleukins
IL10RA	3587	interleukin 1(	CD210|CD210a|CDW2	11	Interleukins_Receptor
IL10RB	3588	interleukin 1(	CDW210B|CRF2-4|CRI	21	Interleukins_Receptor
IL11RA	3590	interleukin 1	CRSDA	9	Interleukins_Receptor

IL12RB1	3594	interleukin 1:	CD212|IL-12R-BETA1	19	Interleukins_Receptor
IL12RB2	3595	interleukin 1:	-	1	Interleukins_Receptor
IL13RA1	3597	interleukin 1:	CD213A1|CT19|IL-1:	X	Interleukins_Receptor
IL13RA2	3598	interleukin 1:	CD213A2|CT19|IL-1:	X	Interleukins_Receptor
IL15RA	3601	interleukin 1!	CD215	10	Interleukins_Receptor
IL2RB	3560	interleukin 2	CD122|IL15RB|IMD6:	22	Interleukins_Receptor
IL17RA	23765	interleukin 1'	CANDF5|CD217|CDw2:	22	Interleukins_Receptor
IL17RB	55540	interleukin 1'	CRL4|EVI27|IL17BR	3	Interleukins_Receptor
IL17RC	84818	interleukin 1'	CANDF9|IL17-RL|IL:	3	Interleukins_Receptor
IL17RD	54756	interleukin 1'	HH18|IL-17RD|IL17I	3	Interleukins_Receptor
IL17RE	1E+05	interleukin 1'	-	3	Interleukins_Receptor
IL18R1	8809	interleukin 1{	CD218a|CDw218a|IL-	2	Interleukins_Receptor
IL18RAP	8807	interleukin 1{	ACPL|CD218b|CDw21{	2	Interleukins_Receptor
IL1R1	3554	interleukin 1	CD121A|D2S1473|IL-	2	Interleukins_Receptor
IL1R2	7850	interleukin 1	CD121b|CDw121b|IL-	2	Interleukins_Receptor
IL1RAP	3556	interleukin 1	C3orf13|IL-1RAcP|:	3	Interleukins_Receptor
IL1RL1	9173	interleukin 1	DER4|FIT-1|IL33R|!	2	Interleukins_Receptor
IL1RL2	8808	interleukin 1	IL-1Rrp2|IL-36R|II	2	Interleukins_Receptor
IL20RA	53832	interleukin 2(	CRF2-8|IL-20R-alpl	6	Interleukins_Receptor
IL20RB	53833	interleukin 2(	DIRS1|FNDC6|IL-20I	3	Interleukins_Receptor
IL21R	50615	interleukin 2:	CD360|IMD56|NILR	16	Interleukins_Receptor
IL22RA1	58985	interleukin 2:	CRF2-9|IL22R|IL22I	1	Interleukins_Receptor
IL22RA2	1E+05	interleukin 2:	CRF2-10|CRF2-S1|CI	6	Interleukins_Receptor
IL23R	1E+05	interleukin 2:	-	1	Interleukins_Receptor
IL27RA	9466	interleukin 2'	CRL1|IL-27RA|IL27I	19	Interleukins_Receptor
IFNLR1	2E+05	interferon la	CRF2/12|IFNLR|IL-:	1	Interleukins_Receptor
IL2RA	3559	interleukin 2	CD25|IDDM10|IL2R|:	10	Interleukins_Receptor
IL2RB	3560	interleukin 2	CD122|IL15RB|IMD6:	22	Interleukins_Receptor
IL2RG	3561	interleukin 2	CD132|CIDX|IL-2RG	X	Interleukins_Receptor
IL31RA	1E+05	interleukin 3:	CRL|CRL3|GLM-R|GLI	5	Interleukins_Receptor
IL3RA	3563	interleukin 3	CD123|IL3R|IL3RAY	X|Y	Interleukins_Receptor
IL4R	3566	interleukin 4	CD124|IL-4RA|IL4R/	16	Interleukins_Receptor
IL5RA	3568	interleukin 5	CD125|CDw125|HSIL!	3	Interleukins_Receptor
IL6R	3570	interleukin 6	CD126|IL-6R-1|IL-(	1	Interleukins_Receptor
IL7R	3575	interleukin 7	CD127|CDW127|IL-7I	5	Interleukins_Receptor
CXCR1	3577	C-X-C motif cl	C-C|C-C-CKR-1|CD1:	2	Interleukins_Receptor
CXCR2	3579	C-X-C motif cl	CD182|CDw128b|CMK/	2	Interleukins_Receptor
IL9R	3581	interleukin 9	CD129|IL-9R	X|Y	Interleukins_Receptor
ST2	6761	-	-	11	Interleukins_Receptor
HLA-A	3105	major histocoı	HLAA	6	NaturalKiller_Cell_Cytotoxicity
HLA-B	3106	major histocoı	AS|B-4901|HLAB	6	NaturalKiller_Cell_Cytotoxicity
HLA-C	3107	major histocoı	D6S204|HLA-JY3|HL/	6	NaturalKiller_Cell_Cytotoxicity
HLA-E	3133	major histocoı	HLA-6.2|QA1	6	NaturalKiller_Cell_Cytotoxicity
HLA-G	3135	major histocoı	MHC-G	6	NaturalKiller_Cell_Cytotoxicity
KIR3DL1	3811	killer cell iı	CD158E1|KIR|KIR3DI	19	NaturalKiller_Cell_Cytotoxicity
KIR3DL2	3812	killer cell iı	3DL2|CD158K|KIR-3I	19	NaturalKiller_Cell_Cytotoxicity
KIR2DL1	3802	killer cell iı	CD158A|KIR-K64|KII	19	NaturalKiller_Cell_Cytotoxicity
KIR2DL2	3803	killer cell iı	CD158B1|CD158b|NK/	19	NaturalKiller_Cell_Cytotoxicity
KIR2DL3	3804	killer cell iı	CD158B2|CD158b|GL:	19	NaturalKiller_Cell_Cytotoxicity
KIR2DL4	3805	killer cell iı	CD158D|G9P|KIR-10:	19	NaturalKiller_Cell_Cytotoxicity
KIR2DL5A	57292	killer cell iı	CD158F|KIR2DL5|KII	19	NaturalKiller_Cell_Cytotoxicity
KLRC1	3821	killer cell l(	CD159A|NKG2|NKG2A	12	NaturalKiller_Cell_Cytotoxicity
KLRC2	3822	killer cell l(	CD159c|NKG2-C|NKG:	12	NaturalKiller_Cell_Cytotoxicity
KLRC3	3823	killer cell l(	NKG2-E|NKG2E	12	NaturalKiller_Cell_Cytotoxicity

KLRD1	3824	killer cell l	CD94	12	NaturalKiller_Cell_Cytotoxicity
PTPN6	5777	protein tyros	HCP|HCPH|HPTP1C|P	12	NaturalKiller_Cell_Cytotoxicity
PTPN11	5781	protein tyros	BPTP3|CFC|JMML|ME	12	NaturalKiller_Cell_Cytotoxicity
ICAM1	3383	intercellular	BB2|CD54|P3.58	19	NaturalKiller_Cell_Cytotoxicity
ICAM2	3384	intercellular	CD102	17	NaturalKiller_Cell_Cytotoxicity
ITGAL	3683	integrin subu	CD11A|LFA-1|LFA1A	16	NaturalKiller_Cell_Cytotoxicity
ITGB2	3689	integrin subu	CD18|LAD|LCAMB|LF	21	NaturalKiller_Cell_Cytotoxicity
PTK2B	2185	protein tyros	CADTK|CAKB|FADK2|	8	NaturalKiller_Cell_Cytotoxicity
VAV3	10451	vav guanine n	-	1	NaturalKiller_Cell_Cytotoxicity
VAV1	7409	vav guanine n	VAV	19	NaturalKiller_Cell_Cytotoxicity
VAV2	7410	vav guanine n	VAV-2	9	NaturalKiller_Cell_Cytotoxicity
RAC1	5879	Rac family sm	MIG5|MRD48|Rac-1|	7	NaturalKiller_Cell_Cytotoxicity
RAC2	5880	Rac family sm	EN-7|Gx|HSPC022|p	22	NaturalKiller_Cell_Cytotoxicity
RAC3	5881	Rac family sm	-	17	NaturalKiller_Cell_Cytotoxicity
PAK1	5058	p21 (RAC1) ac	IDDMSSD|PAKalpha|	11	NaturalKiller_Cell_Cytotoxicity
MAP2K1	5604	mitogen-activ	CFC3|MAPKK1|MEK1|	15	NaturalKiller_Cell_Cytotoxicity
MAP2K2	5605	mitogen-activ	CFC4|MAPKK2|MEK2|	19	NaturalKiller_Cell_Cytotoxicity
MAPK1	5594	mitogen-activ	ERK|ERK-2|ERK2|ER	22	NaturalKiller_Cell_Cytotoxicity
MAPK3	5595	mitogen-activ	ERK-1|ERK1|ERT2|H	16	NaturalKiller_Cell_Cytotoxicity
TNF	7124	tumor necrosi	DIF|TNF-alpha|TNF	6	NaturalKiller_Cell_Cytotoxicity
CSF2	1437	colony stimul	CSF|GMCSF	5	NaturalKiller_Cell_Cytotoxicity
IFNG	3458	interferon ga	IFG|IFI	12	NaturalKiller_Cell_Cytotoxicity
KIR2DS1	3806	killer cell i	CD158H|CD158a|p50.	19	NaturalKiller_Cell_Cytotoxicity
KIR2DS3	3808	killer cell i	NKAT7	19	NaturalKiller_Cell_Cytotoxicity
KIR2DS4	3809	killer cell i	CD158I|KIR-2DS4|K	19	NaturalKiller_Cell_Cytotoxicity
KIR2DS5	3810	killer cell i	CD158G|NKAT9	19	NaturalKiller_Cell_Cytotoxicity
NCR2	9436	natural cytot	CD336|LY95|NK-p44	6	NaturalKiller_Cell_Cytotoxicity
TYROBP	7305	transmembrane	DAP12|KARAP|PLOSL	19	NaturalKiller_Cell_Cytotoxicity
LCK	3932	LCK proto-onc	IMD22|LSK|YT16|p5	1	NaturalKiller_Cell_Cytotoxicity
FCGR3A	2214	Fc fragment o	CD16|CD16A|FCG3|F	1	NaturalKiller_Cell_Cytotoxicity
FCGR3B	2215	Fc fragment o	CD16|CD16A|CD16b|	1	NaturalKiller_Cell_Cytotoxicity
NCR1	9437	natural cytot	CD335|LY94|NK-p46	19	NaturalKiller_Cell_Cytotoxicity
NCR3	3E+05	natural cytot	1C7|CD337|LY117|M	6	NaturalKiller_Cell_Cytotoxicity
FCER1G	2207	Fc fragment o	FCRG	1	NaturalKiller_Cell_Cytotoxicity
CD247	919	CD247 molecul	CD3-ZETA|CD3H|CD3	1	NaturalKiller_Cell_Cytotoxicity
ZAP70	7535	zeta chain of	ADMIO2|IMD48|SRK|	2	NaturalKiller_Cell_Cytotoxicity
SYK	6850	spleen associ	p72-Syk	9	NaturalKiller_Cell_Cytotoxicity
LCP2	3937	lymphocyte cy	SLP-76|SLP76	5	NaturalKiller_Cell_Cytotoxicity
LAT	27040	linker for ac	IMD52|LAT1|pp36	16	NaturalKiller_Cell_Cytotoxicity
PLCG1	5335	phospholipase	NCKAP3|PLC-II|PLC	20	NaturalKiller_Cell_Cytotoxicity
PLCG2	5336	phospholipase	APLAID|FCAS3|PLC-	16	NaturalKiller_Cell_Cytotoxicity
SH3BP2	6452	SH3 domain bi	3BP-2|3BP2|CRBM|C	4	NaturalKiller_Cell_Cytotoxicity
PIK3CA	5290	phosphatidyli	CLAPO|CLOVE|CWS5|	3	NaturalKiller_Cell_Cytotoxicity
PIK3CB	5291	phosphatidyli	P110BETA|PI3K|PI3	3	NaturalKiller_Cell_Cytotoxicity
PIK3CD	5293	phosphatidyli	APDS|IMD14|P110DE	1	NaturalKiller_Cell_Cytotoxicity
PIK3CG	5294	phosphatidyli	PI3CG|PI3K|PI3Kga	7	NaturalKiller_Cell_Cytotoxicity
PIK3R5	23533	phosphoinosit	F730038I15Rik|FOA	17	NaturalKiller_Cell_Cytotoxicity
PIK3R1	5295	phosphoinosit	AGM7|GRB1|IMD36|p	5	NaturalKiller_Cell_Cytotoxicity
PIK3R2	5296	phosphoinosit	MPPH|MPPH1|P85B|p	19	NaturalKiller_Cell_Cytotoxicity
PIK3R3	8503	phosphoinosit	p55|p55-GAMMA|p55	1	NaturalKiller_Cell_Cytotoxicity
FYN	2534	FYN proto-onc	SLK|SYN|p59-FYN	6	NaturalKiller_Cell_Cytotoxicity
SHC2	25759	SHC adaptor p	SCK|SHCB|SLI	19	NaturalKiller_Cell_Cytotoxicity
SHC4	4E+05	SHC adaptor p	RaLP|SHCD	15	NaturalKiller_Cell_Cytotoxicity
SHC3	53358	SHC adaptor p	N-Shc|NSHC|RAI|SH	9	NaturalKiller_Cell_Cytotoxicity

SHC1	6464	SHC adaptor p	SHC|SHCA	1	NaturalKiller_Cell_Cytotoxicity
GRB2	2885	growth factor	ASH|EGFRBP-GRB2|G	17	NaturalKiller_Cell_Cytotoxicity
SOS1	6654	SOS Ras/Rac g	GF1|GGF1|GINGF|HG	2	NaturalKiller_Cell_Cytotoxicity
SOS2	6655	SOS Ras/Rho g	NS9|SOS-2	14	NaturalKiller_Cell_Cytotoxicity
HRAS	3265	HRas proto-on	C-BAS/HAS|C-H-RAS	11	NaturalKiller_Cell_Cytotoxicity
KRAS	3845	KRAS proto-on	'C-K-RAS|C-K-RAS|	12	NaturalKiller_Cell_Cytotoxicity
NRAS	4893	NRAS proto-on	ALPS4|CMNS|N-ras|	1	NaturalKiller_Cell_Cytotoxicity
ARAF	369	A-Raf proto-o	A-RAF|ARAF1|PKS2|	X	NaturalKiller_Cell_Cytotoxicity
BRAF	673	B-Raf proto-o	B-RAF1|B-raf|BRAF	7	NaturalKiller_Cell_Cytotoxicity
RAF1	5894	Raf-1 proto-o	CMD1NN|CRAF|NS5|R	3	NaturalKiller_Cell_Cytotoxicity
MICA	1E+08	MHC class I p	MIC-A|PERB11.1	6	NaturalKiller_Cell_Cytotoxicity
MICB	4277	MHC class I p	PERB11.2	6	NaturalKiller_Cell_Cytotoxicity
ULBP3	79465	UL16 binding	N2DL-3|NKG2DL3|RA	6	NaturalKiller_Cell_Cytotoxicity
ULBP2	80328	UL16 binding	ALCAN-alpha|N2DL2	6	NaturalKiller_Cell_Cytotoxicity
ULBP1	80329	UL16 binding	N2DL-1|NKG2DL1|RA	6	NaturalKiller_Cell_Cytotoxicity
KLRK1	22914	killer cell l	CD314|D12S2489E|K	12	NaturalKiller_Cell_Cytotoxicity
HCST	10870	hematopoietic	DAP10|KAP10|PIK3A	19	NaturalKiller_Cell_Cytotoxicity
CD48	962	CD48 molecule	BCM1|BLAST|BLAST1	1	NaturalKiller_Cell_Cytotoxicity
CD244	51744	CD244 molecul	2B4|NAIL|NKR2B4|N	1	NaturalKiller_Cell_Cytotoxicity
PPP3CA	5530	protein phosp	ACCIID|CALN|CALNA	4	NaturalKiller_Cell_Cytotoxicity
PPP3CB	5532	protein phosp	CALNA2|CALNB|CNA2	10	NaturalKiller_Cell_Cytotoxicity
PPP3CC	5533	protein phosp	CALNA3|CNA3|PP2Bg	8	NaturalKiller_Cell_Cytotoxicity
CHP1	11261	calcineurin l	CHP|SLC9A1BP|SPAX	15	NaturalKiller_Cell_Cytotoxicity
PPP3R1	5534	protein phosp	CALNB1|CNB|CNB1	2	NaturalKiller_Cell_Cytotoxicity
PPP3R2	5535	protein phosp	PPP3RL	9	NaturalKiller_Cell_Cytotoxicity
CHP2	63928	calcineurin l	-	16	NaturalKiller_Cell_Cytotoxicity
NFAT5	10725	nuclear facto	NF-AT5|NFATL1|NFA	16	NaturalKiller_Cell_Cytotoxicity
NFATC1	4772	nuclear facto	NF-ATC|NF-ATc1.2|	18	NaturalKiller_Cell_Cytotoxicity
NFATC2	4773	nuclear facto	NFAT1|NFATP	20	NaturalKiller_Cell_Cytotoxicity
NFATC3	4775	nuclear facto	NF-AT4c|NFAT4|NFA	16	NaturalKiller_Cell_Cytotoxicity
NFATC4	4776	nuclear facto	NF-AT3|NF-ATC4|NF	14	NaturalKiller_Cell_Cytotoxicity
PRKCA	5578	protein kinas	AAG6|PKC-alpha|PK	17	NaturalKiller_Cell_Cytotoxicity
PRKCB	5579	protein kinas	PKC-beta|PKCB|PKC	16	NaturalKiller_Cell_Cytotoxicity
PRKCG	5582	protein kinas	PKC-gamma|PKCC|PK	19	NaturalKiller_Cell_Cytotoxicity
SH2D1B	1E+05	SH2 domain co	EAT2	1	NaturalKiller_Cell_Cytotoxicity
SH2D1A	4068	SH2 domain co	DSHP|EBVS|IMD5|LY	X	NaturalKiller_Cell_Cytotoxicity
IFNGR1	3459	interferon ga	CD119|IFNGR|IMD27	6	NaturalKiller_Cell_Cytotoxicity
IFNGR2	3460	interferon ga	AF-1|IFGR2|IFNGT1	21	NaturalKiller_Cell_Cytotoxicity
IFNA1	3439	interferon al	IFL|IFN|IFN-ALPHA	9	NaturalKiller_Cell_Cytotoxicity
IFNA2	3440	interferon al	IFN-alpha-2|IFN-a	9	NaturalKiller_Cell_Cytotoxicity
IFNA4	3441	interferon al	IFN-alpha4a|INFA4	9	NaturalKiller_Cell_Cytotoxicity
IFNA5	3442	interferon al	IFN-alpha-5|IFN-a	9	NaturalKiller_Cell_Cytotoxicity
IFNA6	3443	interferon al	IFN-alphaK	9	NaturalKiller_Cell_Cytotoxicity
IFNA7	3444	interferon al	IFN-alphaJ|IFNA-J	9	NaturalKiller_Cell_Cytotoxicity
IFNA8	3445	interferon al	IFN-alphaB	9	NaturalKiller_Cell_Cytotoxicity
IFNA10	3446	interferon al	IFN-alphaC	9	NaturalKiller_Cell_Cytotoxicity
IFNA13	3447	interferon al	-	9	NaturalKiller_Cell_Cytotoxicity
IFNA14	3448	interferon al	IFN-alphaH|LEIF2H	9	NaturalKiller_Cell_Cytotoxicity
IFNA16	3449	interferon al	IFN-alpha-16|IFN-	9	NaturalKiller_Cell_Cytotoxicity
IFNA17	3451	interferon al	IFN-alphaI|IFNA|I	9	NaturalKiller_Cell_Cytotoxicity
IFNA21	3452	interferon al	IFN-alphaI|LeIF F	9	NaturalKiller_Cell_Cytotoxicity
IFNB1	3456	interferon be	IFB|IFF|IFN-beta|	9	NaturalKiller_Cell_Cytotoxicity
IFNAR1	3454	interferon al	AVP|IFN-alpha-REC	21	NaturalKiller_Cell_Cytotoxicity
IFNAR2	3455	interferon al	IFN-R|IFN-alpha-R	21	NaturalKiller_Cell_Cytotoxicity

TNFSF10	8743	TNF superfami	APO2L|Apo-2L|CD253	3	NaturalKiller_Cell_Cytotoxicity
TNFRSF10	8793	TNF receptor s	CD264|DCR2|TRAIL-I	8	NaturalKiller_Cell_Cytotoxicity
TNFRSF10	8794	TNF receptor s	CD263|DCR1|DCR1-TN	8	NaturalKiller_Cell_Cytotoxicity
TNFRSF10	8795	TNF receptor s	CD262|DR5|KILLER|I	8	NaturalKiller_Cell_Cytotoxicity
TNFRSF10	8797	TNF receptor s	APO2|CD261|DR4|TRA	8	NaturalKiller_Cell_Cytotoxicity
FASLG	356	Fas ligand	ALPS1B|APT1LG1|APT	1	NaturalKiller_Cell_Cytotoxicity
FAS	355	Fas cell surfa	ALPS1A|APO-1|APT1	10	NaturalKiller_Cell_Cytotoxicity
GZMB	3002	granzyme B	C11|CCPI|CGL-1|CGI	14	NaturalKiller_Cell_Cytotoxicity
PRF1	5551	perforin 1	HPLH2|P1|PFP	10	NaturalKiller_Cell_Cytotoxicity
CASP3	836	caspase 3	CPP32|CPP32B|SCA-1	4	NaturalKiller_Cell_Cytotoxicity
BID	637	BH3 interacti	FP497	22	NaturalKiller_Cell_Cytotoxicity
CD3D	915	CD3d molecule	CD3-DELTA|IMD19|T3	11	TCRsignalingPathway
CD3E	916	CD3e molecule	IMD18|T3E|TCRE	11	TCRsignalingPathway
CD3G	917	CD3g molecule	CD3-GAMMA|IMD17|T3	11	TCRsignalingPathway
CD247	919	CD247 molecul	CD3-ZETA|CD3H|CD3Q	1	TCRsignalingPathway
CD4	920	CD4 molecule	CD4mut	12	TCRsignalingPathway
CD8A	925	CD8a molecule	CD8|Leu2|p32	2	TCRsignalingPathway
CD8B	926	CD8b molecule	CD8B1|LEU2|LY3|LYT	2	TCRsignalingPathway
PTPRC	5788	protein tyros	B220|CD45|CD45R|GI	1	TCRsignalingPathway
LCK	3932	LCK proto-onc	IMD22|LSK|YT16|p56	1	TCRsignalingPathway
FYN	2534	FYN proto-onc	SLK|SYN|p59-FYN	6	TCRsignalingPathway
ZAP70	7535	zeta chain of	ADMIO2|IMD48|SRK|S	2	TCRsignalingPathway
LCP2	3937	lymphocyte cy	SLP-76|SLP76	5	TCRsignalingPathway
LAT	27040	linker for ac	IMD52|LAT1|pp36	16	TCRsignalingPathway
ITK	3702	IL2 inducible	EMT|LPFS1|LYK|PSCT	5	TCRsignalingPathway
TEC	7006	tec protein t	PSCTK4	4	TCRsignalingPathway
NCK1	4690	NCK adaptor p	NCK|NCKalpha|nck-1	3	TCRsignalingPathway
NCK2	8440	NCK adaptor p	GRB4|NCKbeta	2	TCRsignalingPathway
VAV3	10451	vav guanine n	-	1	TCRsignalingPathway
VAV1	7409	vav guanine n	VAV	19	TCRsignalingPathway
VAV2	7410	vav guanine n	VAV-2	9	TCRsignalingPathway
GRAP2	9402	GRB2 related a	GADS|GRAP-2|GRB2L	22	TCRsignalingPathway
GRB2	2885	growth factor	ASH|EGFRBP-GRB2|Gr	17	TCRsignalingPathway
PAK1	5058	p21 (RAC1) ac	IDDMSSD|PAKalpha|a	11	TCRsignalingPathway
PAK2	5062	p21 (RAC1) ac	PAK65|PAKgamma	3	TCRsignalingPathway
PAK3	5063	p21 (RAC1) ac	ARA|MRX30|MRX47|OI	X	TCRsignalingPathway
PAK4	10298	p21 (RAC1) ac	-	19	TCRsignalingPathway
PAK6	56924	p21 (RAC1) ac	PAK5	15	TCRsignalingPathway
PAK5	57144	p21 (RAC1) ac	PAK7	20	TCRsignalingPathway
RHOA	387	ras homolog fa	ARH12|ARHA|EDFAOB	3	TCRsignalingPathway
CDC42	998	cell division	CDC42Hs|G25K|TKS	1	TCRsignalingPathway
PPP3CA	5530	protein phosp	ACCIID|CALN|CALNA	4	TCRsignalingPathway
PPP3CB	5532	protein phosp	CALNA2|CALNB|CNA2	10	TCRsignalingPathway
PPP3CC	5533	protein phosp	CALNA3|CNA3|PP2Bga	8	TCRsignalingPathway
CHP1	11261	calcineurin l	CHP|SLC9A1BP|SPAX9	15	TCRsignalingPathway
PPP3R1	5534	protein phosp	CALNB1|CNB|CNB1	2	TCRsignalingPathway
PPP3R2	5535	protein phosp	PPP3RL	9	TCRsignalingPathway
CHP2	63928	calcineurin l	-	16	TCRsignalingPathway
NFAT5	10725	nuclear facto	NF-AT5|NFATL1|NFAT	16	TCRsignalingPathway
NFATC1	4772	nuclear facto	NF-ATC|NF-ATc1.2|N	18	TCRsignalingPathway
NFATC2	4773	nuclear facto	NFAT1|NFATP	20	TCRsignalingPathway
NFATC3	4775	nuclear facto	NF-AT4c|NFAT4|NFAT	16	TCRsignalingPathway
NFATC4	4776	nuclear facto	NF-AT3|NF-ATC4|NFA	14	TCRsignalingPathway
SOS1	6654	SOS Ras/Rac g	GF1|GGF1|GINGF|HGI	2	TCRsignalingPathway

SOS2	6655	SOS Ras/Rho gu	NS9|SOS-2	14	TCRsignalingPathway
HRAS	3265	HRas proto-onc	C-BAS/HAS|C-H-RAS	11	TCRsignalingPathway
KRAS	3845	KRAS proto-onc	'C-K-RAS|C-K-RAS|C	12	TCRsignalingPathway
NRAS	4893	NRAS proto-onc	ALPS4|CMNS|N-ras|N	1	TCRsignalingPathway
FOS	2353	Fos proto-onco	AP-1|C-FOS|p55	14	TCRsignalingPathway
JUN	3725	Jun proto-onco	AP-1|AP1|c-Jun|cJU	1	TCRsignalingPathway
CARD11	84433	caspase recru	BENTA|BIMP3|CARMA	7	TCRsignalingPathway
BCL10	8915	BCL10 immune s	CARMEN|CIPER|CLAP	1	TCRsignalingPathway
MALT1	10892	MALT1 paracasp	IMD12|MLT|MLT1|PCA	18	TCRsignalingPathway
CHUK	1147	component of	IKBKA|IKK-alpha|IK	10	TCRsignalingPathway
IKBKB	3551	inhibitor of n	IKK-beta|IKK2|IKKB	8	TCRsignalingPathway
IKBKG	8517	inhibitor of n	AMCBX1|EDAID1|FIP-X		TCRsignalingPathway
NFKB1	4790	nuclear facto	CVID12|EBP-1|KBF1	4	TCRsignalingPathway
RELA	5970	RELA proto-onc	CMCU|NFKB3|p65	11	TCRsignalingPathway
NFKBIA	4792	NFKB inhibito	EDAID2|IKBA|MAD-3	14	TCRsignalingPathway
NFKBIB	4793	NFKB inhibito	IKBB|TRIP9	19	TCRsignalingPathway
NFKBIE	4794	NFKB inhibito	IKBE	6	TCRsignalingPathway
CD28	940	CD28 molecule	Tp44	2	TCRsignalingPathway
ICOS	29851	inducible T ce	AILIM|CD278|CVID1	2	TCRsignalingPathway
CD40LG	959	CD40 ligand	CD154|CD40L|HIGM1 X		TCRsignalingPathway
PIK3R5	23533	phosphoinosit	F730038I15Rik|FOAI	17	TCRsignalingPathway
PIK3R1	5295	phosphoinosit	AGM7|GRB1|IMD36|p8	5	TCRsignalingPathway
PIK3R2	5296	phosphoinosit	MPPH|MPPH1|P85B|p8	19	TCRsignalingPathway
PIK3R3	8503	phosphoinosit	p55|p55-GAMMA|p55I	1	TCRsignalingPathway
PIK3CA	5290	phosphatidyli	CLAPO|CLOVE|CWS5|M	3	TCRsignalingPathway
PIK3CB	5291	phosphatidyli	P110BETA|PI3K|PI3I	3	TCRsignalingPathway
PIK3CD	5293	phosphatidyli	APDS|IMD14|P110DEI	1	TCRsignalingPathway
PIK3CG	5294	phosphatidyli	PI3CG|PI3K|PI3Kgan	7	TCRsignalingPathway
AKT3	10000	AKT serine/th	MPPH|MPPH2|PKB-GAM	1	TCRsignalingPathway
AKT1	207	AKT serine/th	AKT|CWS6|PKB|PKB-A	14	TCRsignalingPathway
AKT2	208	AKT serine/th	HIHGHH|PKBB|PKBBET	19	TCRsignalingPathway
MAP3K8	1326	mitogen-activa	AURA2|COT|EST|ESTI	10	TCRsignalingPathway
MAP3K14	9020	mitogen-activa	FTDCR1B|HS|HSNIK|N	17	TCRsignalingPathway
PDCD1	5133	programmed ce	CD279|PD-1|PD1|SLI	2	TCRsignalingPathway
CTLA4	1493	cytotoxic T-ly	ALPS5|CD|CD152|CEI	2	TCRsignalingPathway
PTPN6	5777	protein tyros	HCP|HCPH|HPTP1C|PT	12	TCRsignalingPathway
CBLC	23624	Cbl proto-onco	CBL-3|CBL-SL|RNF57	19	TCRsignalingPathway
CBL	867	Cbl proto-onco	C-CBL|CBL2|FRA11B	11	TCRsignalingPathway
CBLB	868	Cbl proto-onco	Cbl-b|Nbla00127|RN	3	TCRsignalingPathway
IL2	3558	interleukin 2	IL-2|TCGF|lymphok	4	TCRsignalingPathway
IL4	3565	interleukin 4	BCGF-1|BCGF1|BSF-1	5	TCRsignalingPathway
IL5	3567	interleukin 5	EDF|IL-5|TRF	5	TCRsignalingPathway
IL10	3586	interleukin 10	CSIF|GVHDS|IL-10|I	1	TCRsignalingPathway
IFNG	3458	interferon gan	IFG|IFI	12	TCRsignalingPathway
CSF2	1437	colony stimula	CSF|GMCSF	5	TCRsignalingPathway
TNF	7124	tumor necrosis	DIF|TNF-alpha|TNFA	6	TCRsignalingPathway
CDK4	1019	cyclin depende	CMM3|PSK-J3	12	TCRsignalingPathway
RASGRP1	10125	RAS guanyl re	CALDAG-GEFI|CALDAC	15	TCRsignalingPathway
PDK1	5163	pyruvate dehyd	-	2	TCRsignalingPathway
PLCG1	5335	phospholipase	NCKAP3|PLC-II|PLC1	20	TCRsignalingPathway
PRKCQ	5588	protein kinase	PRKCT|nPKC-theta	10	TCRsignalingPathway
TRAC	28755	T cell recepto	IMD7|TCRA|TRA|TRCA	14	TCRsignalingPathway
TRAJ1	28754	T cell recepto	-	14	TCRsignalingPathway
TRAJ2	28753	T cell recepto	-	14	TCRsignalingPathway

TRAJ3	28752	T cell recept‹	-	14	TCRsignalingPathway
TRAJ4	28751	T cell recept‹	-	14	TCRsignalingPathway
TRAJ5	28750	T cell recept‹	-	14	TCRsignalingPathway
TRAJ6	28749	T cell recept‹	-	14	TCRsignalingPathway
TRAJ7	28748	T cell recept‹	-	14	TCRsignalingPathway
TRAJ8	28747	T cell recept‹	-	14	TCRsignalingPathway
TRAJ9	28746	T cell recept‹	-	14	TCRsignalingPathway
TRAJ10	28745	T cell recept‹	-	14	TCRsignalingPathway
TRAJ11	28744	T cell recept‹	-	14	TCRsignalingPathway
TRAJ12	28743	T cell recept‹	-	14	TCRsignalingPathway
TRAJ13	28742	T cell recept‹	-	14	TCRsignalingPathway
TRAJ14	28741	T cell recept‹	-	14	TCRsignalingPathway
TRAJ15	28740	T cell recept‹	-	14	TCRsignalingPathway
TRAJ16	28739	T cell recept‹	-	14	TCRsignalingPathway
TRAJ17	28738	T cell recept‹	-	14	TCRsignalingPathway
TRAJ18	28737	T cell recept‹	-	14	TCRsignalingPathway
TRAJ19	28736	T cell recept‹	-	14	TCRsignalingPathway
TRAJ20	28735	T cell recept‹	-	14	TCRsignalingPathway
TRAJ21	28734	T cell recept‹	-	14	TCRsignalingPathway
TRAJ22	28733	T cell recept‹	-	14	TCRsignalingPathway
TRAJ23	28732	T cell recept‹	-	14	TCRsignalingPathway
TRAJ24	28731	T cell recept‹	-	14	TCRsignalingPathway
TRAJ25	28730	T cell recept‹	-	14	TCRsignalingPathway
TRAJ26	28729	T cell recept‹	-	14	TCRsignalingPathway
TRAJ27	28728	T cell recept‹	-	14	TCRsignalingPathway
TRAJ28	28727	T cell recept‹	-	14	TCRsignalingPathway
TRAJ29	28726	T cell recept‹	-	14	TCRsignalingPathway
TRAJ30	28725	T cell recept‹	-	14	TCRsignalingPathway
TRAJ31	28724	T cell recept‹	-	14	TCRsignalingPathway
TRAJ32	28723	T cell recept‹	-	14	TCRsignalingPathway
TRAJ33	28722	T cell recept‹	-	14	TCRsignalingPathway
TRAJ34	28721	T cell recept‹	-	14	TCRsignalingPathway
TRAJ35	28720	T cell recept‹	-	14	TCRsignalingPathway
TRAJ36	28719	T cell recept‹	-	14	TCRsignalingPathway
TRAJ37	28718	T cell recept‹	-	14	TCRsignalingPathway
TRAJ38	28717	T cell recept‹	-	14	TCRsignalingPathway
TRAJ39	28716	T cell recept‹	-	14	TCRsignalingPathway
TRAJ40	28715	T cell recept‹	-	14	TCRsignalingPathway
TRAJ41	28714	T cell recept‹	-	14	TCRsignalingPathway
TRAJ42	28713	T cell recept‹	-	14	TCRsignalingPathway
TRAJ43	28712	T cell recept‹	-	14	TCRsignalingPathway
TRAJ44	28711	T cell recept‹	-	14	TCRsignalingPathway
TRAJ45	28710	T cell recept‹	-	14	TCRsignalingPathway
TRAJ46	28709	T cell recept‹	-	14	TCRsignalingPathway
TRAJ47	28708	T cell recept‹	-	14	TCRsignalingPathway
TRAJ48	28707	T cell recept‹	-	14	TCRsignalingPathway
TRAJ49	28706	T cell recept‹	-	14	TCRsignalingPathway
TRAJ50	28705	T cell recept‹	-	14	TCRsignalingPathway
TRAJ52	28703	T cell recept‹	-	14	TCRsignalingPathway
TRAJ53	28702	T cell recept‹	-	14	TCRsignalingPathway
TRAJ54	28701	T cell recept‹	-	14	TCRsignalingPathway
TRAJ56	28699	T cell recept‹	-	14	TCRsignalingPathway
TRAJ57	28698	T cell recept‹	-	14	TCRsignalingPathway
TRAJ58	28697	T cell recept‹	-	14	TCRsignalingPathway

TRAJ59	28696	T cell recept	-	14	TCRsignalingPathway
TRAJ61	28694	T cell recept	-	14	TCRsignalingPathway
TRAV1-1	28693	T cell recept	TCRAV1S1|TCRAV7S1	14	TCRsignalingPathway
TRAV1-2	28692	T cell recept	TCRAV1S2|TCRAV7S2	14	TCRsignalingPathway
TRAV2	28691	T cell recept	TCRAV11S1|TCRAV2S	14	TCRsignalingPathway
TRAV3	28690	T cell recept	TCRAV16S1|TCRAV3S	14	TCRsignalingPathway
TRAV4	28689	T cell recept	TCRAV20S1|TCRAV4S	14	TCRsignalingPathway
TRAV5	28688	T cell recept	TCRAV15S1|TCRAV5S	14	TCRsignalingPathway
TRAV7	28686	T cell recept	TCRAV7S1	14	TCRsignalingPathway
TRAV8-1	28685	T cell recept	TCRAV1S1|TCRAV8S1	14	TCRsignalingPathway
TRAV8-2	28684	T cell recept	TCRAV1S5|TCRAV8S2	14	TCRsignalingPathway
TRAV8-3	28683	T cell recept	TCRAV1S4|TCRAV8S3	14	TCRsignalingPathway
TRAV8-4	28682	T cell recept	TCRAV1S2|TCRAV8S4	14	TCRsignalingPathway
TRAV8-6	28680	T cell recept	TCRAV1S3|TCRAV8S6	14	TCRsignalingPathway
TRAV8-7	28679	T cell recept	TCRAV8S7|TRAV87	14	TCRsignalingPathway
TRAV9-1	28678	T cell recept	TCRAV9S1|TRAV91	14	TCRsignalingPathway
TRAV9-2	28677	T cell recept	TCRAV22S1|TCRAV9S	14	TCRsignalingPathway
TRAV10	28676	T cell recept	TCRAV10S1|TCRAV24	14	TCRsignalingPathway
TRAV12-1	28674	T cell recept	TCRAV12S1|TCRAV2S	14	TCRsignalingPathway
TRAV12-2	28673	T cell recept	TCRAV12S2|TCRAV2S	14	TCRsignalingPathway
TRAV12-3	28672	T cell recept	TCRAV12S3|TCRAV2S	14	TCRsignalingPathway
TRAV13-1	28671	T cell recept	TCRAV13S1|TCRAV8S	14	TCRsignalingPathway
TRAV13-2	28670	T cell recept	TCRAV13S2|TCRAV8S	14	TCRsignalingPathway
TRAV14DV	28669	T cell recept	TCRAV6S1-hDV104S1	14	TCRsignalingPathway
TRAV16	28667	T cell recept	TCRAV16S1|TCRAV9S	14	TCRsignalingPathway
TRAV17	28666	T cell recept	TCRAV17S1|TCRAV3S	14	TCRsignalingPathway
TRAV18	28665	T cell recept	TCRAV18S1	14	TCRsignalingPathway
TRAV19	28664	T cell recept	TCRAV12S1|TCRAV19	14	TCRsignalingPathway
TRAV20	28663	T cell recept	TCRAV20S1|TCRAV30	14	TCRsignalingPathway
TRAV21	28662	T cell recept	TCRAV21S1|TCRAV23	14	TCRsignalingPathway
TRAV22	28661	T cell recept	TCRAV13S1|TCRAV22	14	TCRsignalingPathway
TRAV23DV	28660	T cell recept	TCRAV17S1|TRAV23/	14	TCRsignalingPathway
TRAV24	28659	T cell recept	TCRAV18S1|TCRAV24	14	TCRsignalingPathway
TRAV25	28658	T cell recept	TCRAV25S1|TCRAV32	14	TCRsignalingPathway
TRAV26-1	28657	T cell recept	TCRAV26S1|TCRAV4S	14	TCRsignalingPathway
TRAV26-2	28656	T cell recept	TCRAV26S2|TCRAV4S	14	TCRsignalingPathway
TRAV27	28655	T cell recept	TCRAV10S1|TCRAV27	14	TCRsignalingPathway
TRAV29DV	28653	T cell recept	TCRA|TCRAV21S1|TR	14	TCRsignalingPathway
TRAV30	28652	T cell recept	TCRAV29S1|TCRAV30	14	TCRsignalingPathway
TRAV34	28648	T cell recept	TCRAV26S1|TCRAV34	14	TCRsignalingPathway
TRAV35	28647	T cell recept	TCRAV25S1|TCRAV35	14	TCRsignalingPathway
TRAV36DV	28646	T cell recept	TCRAV28S1|TRAV36/	14	TCRsignalingPathway
TRAV38-1	28644	T cell recept	TCRAV14S2|TCRAV38	14	TCRsignalingPathway
TRAV38-2	28643	T cell recept	TCRAV14S1|TRAV382	14	TCRsignalingPathway
TRAV39	28642	T cell recept	TCRAV27S1|TCRAV39	14	TCRsignalingPathway
TRAV40	28641	T cell recept	TCRAV31S1|TCRAV40	14	TCRsignalingPathway
TRAV41	28640	T cell recept	TCRAV19S1|TCRAV41	14	TCRsignalingPathway
TRBC1	28639	T cell recept	BV05S1J2.2|TCRB|T	7	TCRsignalingPathway
TRBC2	28638	T cell recept	TCRBC2	7	TCRsignalingPathway
TRBD1	28637	T cell recept	TCRBD1	7	TCRsignalingPathway
TRBD2	28636	T cell recept	TCRBD2	7	TCRsignalingPathway
TRBJ1-1	28635	T cell recept	TCRBJ1S1|TRBJ11	7	TCRsignalingPathway
TRBJ1-2	28634	T cell recept	TCRBJ1S2|TRBJ12	7	TCRsignalingPathway
TRBJ1-3	28633	T cell recept	TCRBJ1S3|TRBJ13	7	TCRsignalingPathway

TRBJ1-4	28632	T cell recept	TCRBJ1S4|TRBJ14	7	TCRsignalingPathway
TRBJ1-5	28631	T cell recept	TCRBJ1S5|TRBJ15	7	TCRsignalingPathway
TRBJ1-6	28630	T cell recept	TCRBJ1S6|TRBJ16	7	TCRsignalingPathway
TRBJ2-1	28629	T cell recept	TCRBJ2S1|TRBJ21	7	TCRsignalingPathway
TRBJ2-2	28628	T cell recept	TCRBJ2S2|TRBJ22	7	TCRsignalingPathway
TRBJ2-3	28626	T cell recept	TCRBJ2S3|TRBJ23	7	TCRsignalingPathway
TRBJ2-4	28625	T cell recept	TCRBJ2S4|TRBJ24	7	TCRsignalingPathway
TRBJ2-5	28624	T cell recept	TCRBJ2S5|TRBJ25	7	TCRsignalingPathway
TRBJ2-6	28623	T cell recept	TCRBJ2S6|TRBJ26	7	TCRsignalingPathway
TRBJ2-7	28622	T cell recept	TCRBJ2S7|TRBJ27	7	TCRsignalingPathway
TRBV2	28620	T cell recept	TCRBV22S1A2N1T|TC	7	TCRsignalingPathway
TRBV3-1	28619	T cell recept	TCRBV3S1|TCRBV9S1	7	TCRsignalingPathway
TRBV4-1	28617	T cell recept	BV07S1J2.7|TCRBV4	7	TCRsignalingPathway
TRBV4-2	28616	T cell recept	TCRBV4S2|TCRBV7S3	7	TCRsignalingPathway
TRBV4-3	28615	T cell recept	TCRBV4S3|TCRBV7S2	7	TCRsignalingPathway
TRBV5-1	28614	T cell recept	TCRBV5S1|TCRBV5S1	7	TCRsignalingPathway
TRBV5-4	28611	T cell recept	TCRBV5S4|TCRBV5S6	7	TCRsignalingPathway
TRBV5-5	28610	T cell recept	TCRBV5S3A2T|TCRBV	7	TCRsignalingPathway
TRBV5-6	28609	T cell recept	TCRBV5S2|TCRBV5S6	7	TCRsignalingPathway
TRBV5-7	28608	T cell recept	TCRBV5S7|TCRBV5S7	7	TCRsignalingPathway
TRBV5-8	28607	T cell recept	TCRBV5S4A2T|TCRBV	7	TCRsignalingPathway
TRBV6-1	28606	T cell recept	TCRBV13S3|TCRBV6S	7	TCRsignalingPathway
TRBV6-2	28605	T cell recept	TCRBV13S2|TCRBV13	7	TCRsignalingPathway
TRBV6-3	28604	T cell recept	TCRBV13S9/13S2A1T	7	TCRsignalingPathway
TRBV6-4	28603	T cell recept	TCRBV13S5|TCRBV6S	7	TCRsignalingPathway
TRBV6-5	28602	T cell recept	TCRBV13S1|TCRBV6S	7	TCRsignalingPathway
TRBV6-6	28601	T cell recept	TCRBV13S6A2T|TCRB	7	TCRsignalingPathway
TRBV6-7	28600	T cell recept	TCRBV13S8P|TCRBV6	7	TCRsignalingPathway
TRBV6-8	28599	T cell recept	TCRBV13S7P|TCRBV6	7	TCRsignalingPathway
TRBV6-9	28598	T cell recept	TCRBV13S4|TCRBV6S	7	TCRsignalingPathway
TRBV7-2	28596	T cell recept	TCRBV6S5A1N1|TCRB	7	TCRsignalingPathway
TRBV7-3	28595	T cell recept	TCRBV6S1A1N1|TCRB	7	TCRsignalingPathway
TRBV7-4	28594	T cell recept	TCRBV6S8A2T|TCRBV	7	TCRsignalingPathway
TRBV7-6	28592	T cell recept	TCRBV6S3A1N1T|TCR	7	TCRsignalingPathway
TRBV7-7	28591	T cell recept	TCRBV6S6A2T|TCRBV	7	TCRsignalingPathway
TRBV7-8	28590	T cell recept	TCRBV6S2A1N1T|TCR	7	TCRsignalingPathway
TRBV7-9	28589	T cell recept	TCRB|TCRBV6S4A1|T	7	TCRsignalingPathway
TRBV9	28586	T cell recept	TCRBV1S1A1N1|TCRB	7	TCRsignalingPathway
TRBV10-1	28585	T cell recept	TCRBV10S1|TCRBV12	7	TCRsignalingPathway
TRBV10-2	28584	T cell recept	TCRBV10S2|TCRBV12	7	TCRsignalingPathway
TRBV10-3	28583	T cell recept	TCRBV10S3|TCRBV12	7	TCRsignalingPathway
TRBV11-1	28582	T cell recept	TCRBV11S1|TCRBV21	7	TCRsignalingPathway
TRBV11-2	28581	T cell recept	TCRBV11S2|TCRBV21	7	TCRsignalingPathway
TRBV11-3	28580	T cell recept	TCRBV11S3|TCRBV21	7	TCRsignalingPathway
TRBV12-3	28577	T cell recept	TCRBV12S3|TCRBV8S	7	TCRsignalingPathway
TRBV12-4	28576	T cell recept	TCRBV12S4|TCRBV8S	7	TCRsignalingPathway
TRBV12-5	28575	T cell recept	TCRBV12S5|TCRBV8S	7	TCRsignalingPathway
TRBV13	28574	T cell recept	TCRBV13S1|TCRBV23	7	TCRsignalingPathway
TRBV14	28573	T cell recept	TCRBV14S1|TCRBV16	7	TCRsignalingPathway
TRBV15	28572	T cell recept	TCRBV15S1|TCRBV24	7	TCRsignalingPathway
TRBV16	28571	T cell recept	BV25S1J1.2|TCRB|T	7	TCRsignalingPathway
TRBV17	28570	T cell recept	TCRBV17S1|TCRBV26	7	TCRsignalingPathway
TRBV18	28569	T cell recept	TCRBV18S1	7	TCRsignalingPathway
TRBV19	28568	T cell recept	TCRBV17S1A1T|TCRB	7	TCRsignalingPathway

TRBV20-1	28567	T cell recept	TCRBV20S1|TCRBV2S	7	TCRsignalingPathway
TRBV24-1	28563	T cell recept	TCRBV15S1|TCRBV24S	7	TCRsignalingPathway
TRBV25-1	28562	T cell recept	TCRBV11S1A1T|TCRB	7	TCRsignalingPathway
TRBV27	28560	T cell recept	TCRBV14S1|TCRBV27S	7	TCRsignalingPathway
TRBV28	28559	T cell recept	TCRBV28S1|TCRBV3S	7	TCRsignalingPathway
TRBV29-1	28558	T cell recept	TCRBV29S1|TCRBV4S	7	TCRsignalingPathway
TRBV30	28557	T cell recept	TCRBV20S1A1N2|TCR	7	TCRsignalingPathway
TRDC	28526	T cell recept	TCRD	14	TCRsignalingPathway
TRDD1	28525	T cell recept	-	14	TCRsignalingPathway
TRDD2	28524	T cell recept	-	14	TCRsignalingPathway
TRDD3	28523	T cell recept	TCRD	14	TCRsignalingPathway
TRDJ1	28522	T cell recept	TCRD	14	TCRsignalingPathway
TRDJ2	28521	T cell recept	-	14	TCRsignalingPathway
TRDJ3	28520	T cell recept	-	14	TCRsignalingPathway
TRDJ4	28519	T cell recept	-	14	TCRsignalingPathway
TRDV1	28518	T cell recept	hDV101S1	14	TCRsignalingPathway
TRDV2	28517	T cell recept	hDV102S1	14	TCRsignalingPathway
TRDV3	28516	T cell recept	hDV103S1	14	TCRsignalingPathway
TRGV9	6983	T cell recept	TCRGV9|TRGC1|V2	7	TCRsignalingPathway
TRGV8	6982	T cell recept	TCRGV8|V1S8	7	TCRsignalingPathway
TRGV5	6978	T cell recept	TCRGV5|V1S5	7	TCRsignalingPathway
TRGV4	6977	T cell recept	TCRGV4|V1S4	7	TCRsignalingPathway
TRGV3	6976	T cell recept	TCRGV3|V1S3	7	TCRsignalingPathway
TRGV2	6974	T cell recept	TCRGV2|VIS2	7	TCRsignalingPathway
TRGJP2	6972	T cell recept	JP2|TCRGJP2	7	TCRsignalingPathway
TRGJP1	6971	T cell recept	JP1|TCRGJP1	7	TCRsignalingPathway
TRGJP	6970	T cell recept	JP|TCRGJP	7	TCRsignalingPathway
TRGJ2	6969	T cell recept	J2|TCRGJ2	7	TCRsignalingPathway
TRGJ1	6968	T cell recept	J1|TCRGJ1	7	TCRsignalingPathway
TRGC2	6967	T cell recept	TCRGC2|TRGC2(2X)|	7	TCRsignalingPathway
TRGC1	6966	T cell recept	C1|TCRG|TCRGC1	7	TCRsignalingPathway
TRAV6	6956	T cell recept	TCRAV5S1|TCRAV6S1	14	TCRsignalingPathway
BMP1	649	bone morphoge	OI13|PCOLC|PCP|PC	8	TGFb_Family_Member
BMP10	27302	bone morphoge	-	2	TGFb_Family_Member
BMP15	9210	bone morphoge	GDF9B|ODG2|POF4	X	TGFb_Family_Member
BMP2	650	bone morphoge	BDA2|BMP2A|SSFSC	20	TGFb_Family_Member
BMP3	651	bone morphoge	BMP-3A	4	TGFb_Family_Member
BMP4	652	bone morphoge	BMP2B|BMP2B1|MCOP	14	TGFb_Family_Member
BMP5	653	bone morphoge	-	6	TGFb_Family_Member
BMP6	654	bone morphoge	VGR|VGR1	6	TGFb_Family_Member
BMP7	655	bone morphoge	OP-1	20	TGFb_Family_Member
BMP8A	4E+05	bone morphoge	OP-2	1	TGFb_Family_Member
BMP8B	656	bone morphoge	BMP8|OP2	1	TGFb_Family_Member
GDF1	2657	growth differ	CERS1|CHTD6|DORV|	19	TGFb_Family_Member
GDF10	2662	growth differ	BIP|BMP-3b|BMP3B	10	TGFb_Family_Member
GDF11	10220	growth differ	BMP-11|BMP11	12	TGFb_Family_Member
GDF15	9518	growth differ	GDF-15|MIC-1|MIC1	19	TGFb_Family_Member
GDF2	2658	growth differ	BMP-9|BMP9|HHT5	10	TGFb_Family_Member
GDF3	9573	growth differ	KFS3|MCOP7|MCOPCB	12	TGFb_Family_Member
GDF5	8200	growth differ	BDA1C|BMP-14|BMP1	20	TGFb_Family_Member
GDF6	4E+05	growth differ	BMP-13|BMP13|CDMP	8	TGFb_Family_Member
GDF7	2E+05	growth differ	BMP12	2	TGFb_Family_Member
GDF9	2661	growth differ	POF14	5	TGFb_Family_Member
GDNF	2668	glial cell de	ATF|ATF1|ATF2|HFB	5	TGFb_Family_Member

INHA	3623	inhibin subun	-	2	TGFb_Family_Member
INHBA	3624	inhibin subun	EDF|FRP	7	TGFb_Family_Member
INHBB	3625	inhibin subun	-	2	TGFb_Family_Member
INHBC	3626	inhibin subun	IHBC	12	TGFb_Family_Member
INHBE	83729	inhibin subun	-	12	TGFb_Family_Member
LEFTY1	10637	left-right de	LEFTB|LEFTYB	1	TGFb_Family_Member
LEFTY2	7044	left-right de	EBAF|LEFTA|LEFTYA	1	TGFb_Family_Member
NODAL	4838	nodal growth (	HTX5	10	TGFb_Family_Member
TGFB1	7040	transforming {	CED|DPD1|IBDIMDE|I	19	TGFb_Family_Member
TGFB2	7042	transforming {	G-TSF|LDS4|TGF-be	1	TGFb_Family_Member
TGFB3	7043	transforming {	ARVD|ARVD1|LDS5|RI	14	TGFb_Family_Member
ACVR1B	91	activin A rec	ACTRIB|ACVRLK4|ALI	12	TGFb_Family_Member_Receptor
ACVR1C	1E+05	activin A rec	ACVRLK7|ALK7	2	TGFb_Family_Member_Receptor
ACVR2A	92	activin A rec	ACTRII|ACVR2	2	TGFb_Family_Member_Receptor
ACVR2B	93	activin A rec	ACTRIIB|ActR-IIB|I	3	TGFb_Family_Member_Receptor
ACVRL1	94	activin A rec	ACVRLK1|ALK-1|ALK	12	TGFb_Family_Member_Receptor
AMHR2	269	anti-Mulleria	AMHR|MISR2|MISRII	12	TGFb_Family_Member_Receptor
BMPR1A	657	bone morphoge	10q23del|ACVRLK3|	10	TGFb_Family_Member_Receptor
BMPR1B	658	bone morphoge	ALK-6|ALK6|AMDD|BI	4	TGFb_Family_Member_Receptor
BMPR2	659	bone morphoge	BMPR-II|BMPR3|BMR	2	TGFb_Family_Member_Receptor
TGFBR1	7046	transforming {	AAT5|ACVRLK4|ALK-	9	TGFb_Family_Member_Receptor
TGFBR2	7048	transforming {	AAT3|FAA3|LDS1B|LI	3	TGFb_Family_Member_Receptor
TGFBR3	7049	transforming {	BGCAN|betaglycan	1	TGFb_Family_Member_Receptor
TNFRSF11	4982	TNF receptor :	OCIF|OPG|PDB5|TR1	8	TNF_Family_Members
TNFSF10	8743	TNF superfami	APO2L|Apo-2L|CD25	3	TNF_Family_Members
TNFSF11	8600	TNF superfami	CD254|ODF|OPGL|OP	13	TNF_Family_Members
TNFSF12	8742	TNF superfami	APO3L|DR3LG|TNLG4	17	TNF_Family_Members
TNFSF13	8741	TNF superfami	APRIL|CD256|TALL-	17	TNF_Family_Members
TNFSF13B	10673	TNF superfami	BAFF|BLYS|CD257|D	13	TNF_Family_Members
TNFSF14	8740	TNF superfami	CD258|HVEML|LIGHT	19	TNF_Family_Members
TNFSF15	9966	TNF superfami	TL1|TL1A|TNLG1B|VI	9	TNF_Family_Members
TNFSF18	8995	TNF superfami	AITRL|GITRL|TL6|TI	1	TNF_Family_Members
TNFSF4	7292	TNF superfami	CD134L|CD252|GP34	1	TNF_Family_Members
TNFSF8	944	TNF superfami	CD153|CD30L|CD30L(	9	TNF_Family_Members
TNFSF9	8744	TNF superfami	4-1BB-L|CD137L|TNI	19	TNF_Family_Members
TNFRSF10	8795	TNF receptor :	CD262|DR5|KILLER|I	8	TNF_Family_Members_Receptors
TNFRSF10	8794	TNF receptor :	CD263|DCR1|DCR1-TI	8	TNF_Family_Members_Receptors
TNFRSF10	8793	TNF receptor :	CD264|DCR2|TRAIL-I	8	TNF_Family_Members_Receptors
TNFRSF11	8792	TNF receptor :	CD265|FEO|LOH18CR	18	TNF_Family_Members_Receptors
TNFRSF12	51330	TNF receptor :	CD266|FN14|TWEAKR	16	TNF_Family_Members_Receptors
TNFRSF13	23495	TNF receptor :	CD267|CVID|CVID2|	17	TNF_Family_Members_Receptors
TNFRSF13	1E+05	TNF receptor :	BAFF-R|BAFFR|BROM	22	TNF_Family_Members_Receptors
TNFRSF14	8764	TNF receptor :	ATAR|CD270|HVEA|H	1	TNF_Family_Members_Receptors
TNFRSF17	608	TNF receptor :	BCM|BCMA|CD269|TNI	16	TNF_Family_Members_Receptors
TNFRSF18	8784	TNF receptor :	AITR|CD357|ENERGEI	1	TNF_Family_Members_Receptors
TNFRSF19	55504	TNF receptor :	TAJ|TAJ-alpha|TRAI	13	TNF_Family_Members_Receptors
TNFRSF1A	7132	TNF receptor :	CD120a|FPF|TBP1|TI	12	TNF_Family_Members_Receptors
TNFRSF1B	7133	TNF receptor :	CD120b|TBPII|TNF-I	1	TNF_Family_Members_Receptors
TNFRSF21	27242	TNF receptor :	BM-018|CD358|DR6	6	TNF_Family_Members_Receptors
TNFRSF25	8718	TNF receptor :	APO-3|DDR3|DR3|GEI	1	TNF_Family_Members_Receptors
TNFRSF4	7293	TNF receptor :	ACT35|CD134|IMD16	1	TNF_Family_Members_Receptors
TNFRSF6B	8771	TNF receptor :	DCR3|DJ583P15.1.1	20	TNF_Family_Members_Receptors
TNFRSF8	943	TNF receptor :	CD30|D1S166E|Ki-1	1	TNF_Family_Members_Receptors
TNFRSF9	3604	TNF receptor :	4-1BB|CD137|CDw13	1	TNF_Family_Members_Receptors
