# Supplementary figures and images for: Development of a Risk Predictive Model for Evaluating Immune Infiltration Status in Invasive Thyroid Carcinoma
Source: Evid Based Complement Alternat Med. 2022 Jun 3;2022:5803077. doi: 10.1155/2022/5803077 (PMC9187459; doi:10.1155/2022/5803077)

A

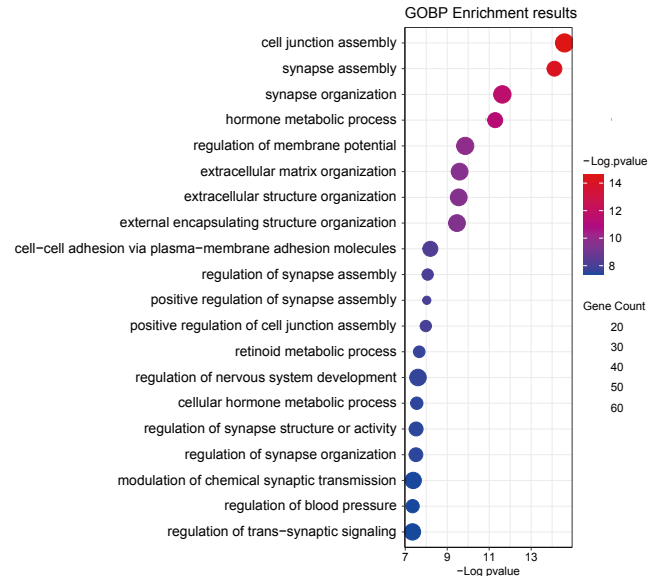

B

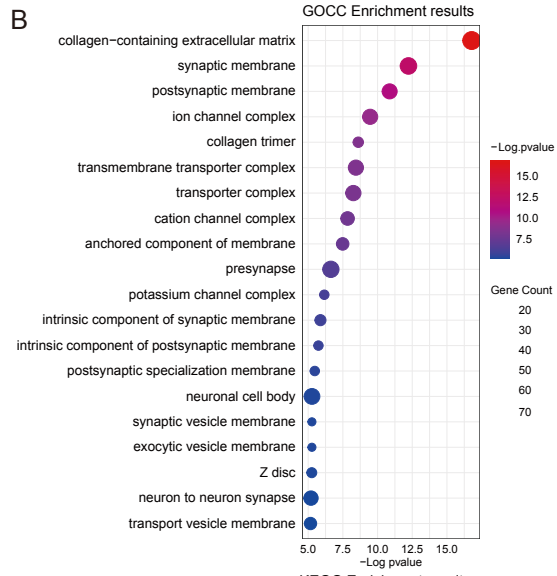

C

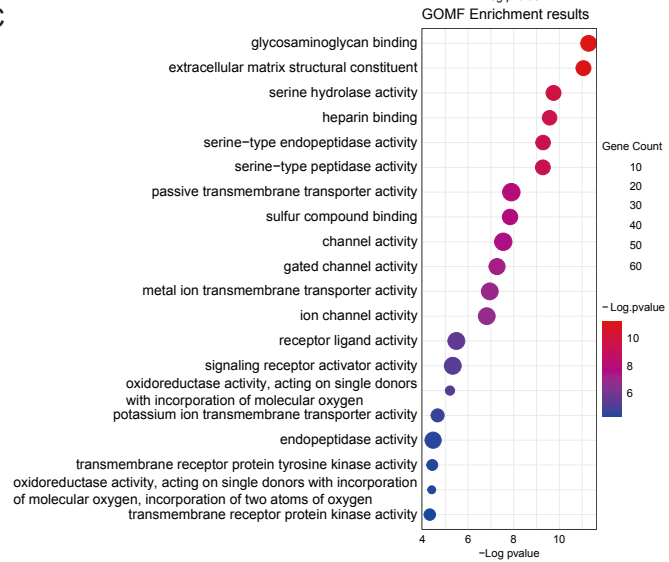

D

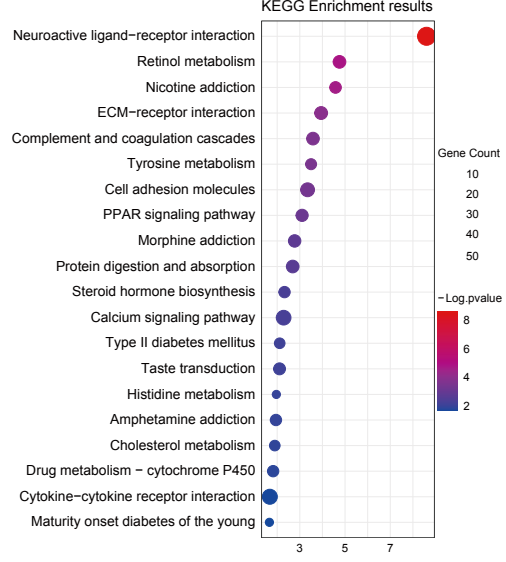

Supplement: Supplementary Materials — Figure S1: functional enrichment analysis for DEG between tumor and normal control. The top 20 enriched biological processes (A), cellular component (B), and molecular function (C) terms in gene ontology annotation; (D) the top 20 enriched KEGG pathways. Figure S2: GSEA for DEGs between immune-activated and immunosuppressive groups. A, the bubble diagram shows the top 20 activated and suppressed pathways in GSEA analysis; B, the top 10 pathways in GSEA ranked by NES value. Table S1: the 29 immune related gene sets. Table S2: immune-related genes obtained from the ImmPort database. [file 5803077.f1.zip › 5803077.f1/Figure S1 (1).pdf]

A

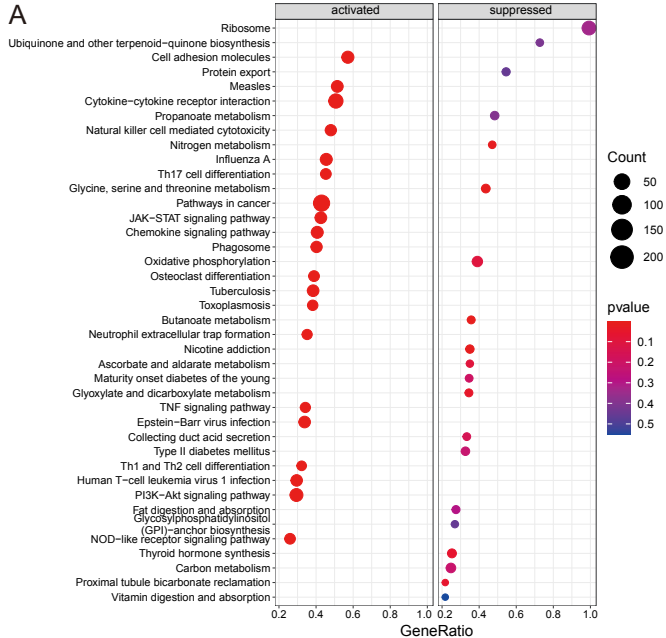

B

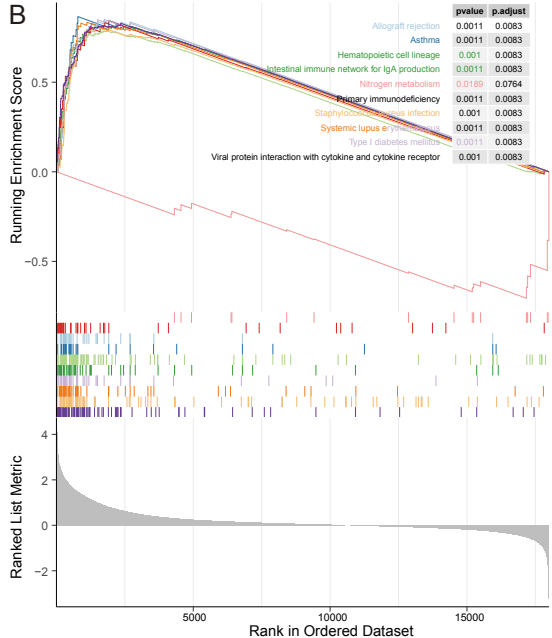

Supplement: Supplementary Materials — Figure S1: functional enrichment analysis for DEG between tumor and normal control. The top 20 enriched biological processes (A), cellular component (B), and molecular function (C) terms in gene ontology annotation; (D) the top 20 enriched KEGG pathways. Figure S2: GSEA for DEGs between immune-activated and immunosuppressive groups. A, the bubble diagram shows the top 20 activated and suppressed pathways in GSEA analysis; B, the top 10 pathways in GSEA ranked by NES value. Table S1: the 29 immune related gene sets. Table S2: immune-related genes obtained from the ImmPort database. [file 5803077.f1.zip › 5803077.f1/Figure S2 (1).pdf]
